# Species-specific Loss of Genetic Diversity and Inbreeding Following Agricultural Intensification

**DOI:** 10.1093/gbe/evag235

**Published:** 2026-09-24

**Authors:** Zachary J Nolen, Patrycja Jamelska, Ana Sofia Torres Lara, Niklas Wahlberg, Anna Runemark

**Affiliations:** Department of Biology, Lund University, Lund 22362, Sweden; Department of Biology, Lund University, Lund 22362, Sweden; Department of Biology, Lund University, Lund 22362, Sweden; Faculty 2 Biology/Chemistry, University of Bremen, Bremen 28359, Germany; Department of Biology, Lund University, Lund 22362, Sweden; Department of Biology, Lund University, Lund 22362, Sweden

**Keywords:** conservation, agricultural intensification, inbreeding, genetic load, genomic erosion

## Abstract

Land-use change driven by agricultural intensification is a major contributor to insect declines globally. As a result of population decline and subdivision, species lose genetic diversity, reducing adaptive potential and increasing genetic load. We examine how genetic diversity has changed over the past century in three focal and five additional Polyommatinae butterfly species in Sweden by sequencing specimens from museum collections. Based on 59 historical and 90 contemporary genomes, we document a 6% decline in heterozygosity in the grassland specialist *Cyaniris semiargus*, driven by division into small, isolated populations. In contrast, generalist *Polyommatus icarus* and heathland specialist *Plebejus argus* have maintained genetic diversity and connectivity. Reduced heterozygosity in *Cy. semiargus* reflects increases in runs of homozygosity, increasing the homozygosity of derived mutations at missense and conserved positions. While genomic erosion is currently relatively mild, it is expected to lag demographic change. We infer that demographic decline in *Cy. semiargus* is considerably larger than declines in genetic diversity to date. We use simulations to demonstrate that only mild losses of genetic diversity are expected at the early stages of demographic decline, leaving populations with substantial genetic extinction debt. We detect similar reductions in genetic diversity in two of five additional species, underscoring the need for restoring habitat connectivity and gene flow to limit further genomic erosion in specialist grassland insects. This study illustrates how incorporation of genetic monitoring into conservation planning can identify where demographic decline has left populations with a genetic extinction debt even after they stabilize.

Significance statement Insects are declining globally, largely due to land use change. To which extent genetic diversity decline threatens insect populations is unclear, as studies of genetic diversity decline largely focus on vertebrates. Using historical and modern genomes from three grassland butterfly species, we uncover increased population isolation and rates of inbreeding in specialist *Cyaniris semiargus* following agricultural intensification, though its conservation status is “Least Concern.” This contrasts with two less specialized species with no changes in genetic diversity. The genetic diversity of *Cy. semiargus* is expected to continue to decline even if populations stabilize as it lags much larger demographic declines. These results highlight that the stronger declines seen in specialist grassland insect abundance have been sufficient to result in genetic diversity loss, and raises concerns that restoration of suitable habitat is needed to prevent further losses which threaten long-term persistence.

## Introduction

Insect decline has received increased attention in recent years as reports uncover alarming declines in insect assemblages and biomass (Wagner et al. 2021b). Efforts to quantify this decline estimate that terrestrial insects are declining at an average rate of 11% per decade globally, with this trend being primarily driven by even stronger regional declines in Europe and North America (van Klink et al. 2020). This stark decline is the result of a variety of anthropogenic pressures, primarily climate change and land use changes such as agricultural intensification and urbanization (Dirzo et al. 2014; Seibold et al. 2019; Boyes et al. 2021; Halsch et al. 2021; Raven and Wagner 2021; Wagner et al. 2021b). In Europe, formerly biodiverse semi-natural grasslands have been abandoned or converted into intensively cultivated land (Shipley et al. 2024), placing a strong pressure on grassland insect species (Seibold et al. 2019).

While insect declines have alarming implications for the natural communities and ecosystem services they play key roles in, declines also lead to genomic erosion within insect populations themselves. As populations decline, the relative strength of drift increases (Lacy 1987), eroding genetic diversity, thereby reducing adaptive potential (Lande 1995; Lande and Shannon 1996). Additionally, in small, isolated populations, deleterious mutations are more likely to accumulate through drift and become unmasked through inbreeding (Lande 1994, 1998; Lynch et al. 1995; Wang et al. 1999; van Oosterhout 2020; Bertorelle et al. 2022; Dussex et al. 2023), further reinforcing population decline (Gilpin and Soule 1986; Höglund 2009). Mitigation of genomic erosion that threatens population persistence has been made a part of Goal A of the Kunming-Montréal Global Biodiversity Framework, which stipulates maintenance of genetic diversity to preserve the adaptive potential of wild populations (Convention on Biological Diversity 2022).

Understanding early signals of genomic erosion is key for intervention before substantial diversity is irreversibly lost and monitoring programs to assess genomic erosion are still under development. While plans to monitor genetic diversity in the wild have been implemented in several countries (Díez-del-Molino et al. 2018; Andersson et al. 2022; O’Brien et al. 2022; Theissinger et al. 2023), interpreting measures of genetic diversity remains a challenge. First, genomic erosion lags disturbance events, and current estimates of genetic diversity will therefore not capture the genetic extinction debt of populations (Epps and Keyghobadi 2015; Mualim et al. 2026; Pinto et al. 2024; Fontsere et al. 2025; Gargiulo et al. 2025; Liu et al. 2025). Therefore, identifying measures that enable early detection of genomic erosion is a priority. Second, absolute levels of genetic diversity are influenced by a multitude of factors not related to threat level and vary considerably across taxa and populations (Hewitt 2000; Romiguier et al. 2014; Ellegren and Galtier 2016; Mackintosh et al. 2019). To control for variation in baseline levels of genetic diversity, programs in several countries supplement estimates of contemporary effective population sizes with measures of genetic diversity metrics across multiple time points (Schwartz et al. 2007; O’Brien et al. 2022).

Temporal estimates of genetic diversity enable direct quantification of genomic erosion but are limited to detecting changes after the first sampled time point. To overcome this, genomic resources from historic samples preserved in natural history collections provide an opportunity to establish baseline estimates of genetic diversity from before major anthropogenically driven insect declines (Díez-del-Molino et al. 2018; Webster et al. 2023). By combining museum specimens with contemporary sampling from the same populations it is possible to directly identify ongoing genomic erosion, minimizing time to detection of populations at risk. Several studies have sequenced whole genomes from museum specimens to identify reductions in heterozygosity and changes in genetic load in species that experienced large bottlenecks (e.g. van der Valk et al. 2019; Dussex et al. 2021; Femerling et al. 2023; Clark et al. 2025; Fontsere et al. 2025). However, only a few such studies have applied these methods to examine responses to insect decline (e.g. Gauthier et al. 2020; Parejo et al. 2020).

Here, we use museomics to assess to what extent genetic diversity and connectivity has been lost in grassland associated butterflies during the second wave of agricultural modernization in Sweden (Ihse 1995; Lindborg and Eriksson 2004). Starting in the 1940s, small farms were abandoned, and their associated semi-natural grasslands began to decline considerably (Lindborg and Eriksson 2004). We examine three focal butterfly species with Swedish Red List statuses of “Least Concern” (SLU Artdatabanken 2020) in this landscape. These species vary in habitat specialization, ranging from grassland generalist to specialist, enabling us to examine if patterns of genetic diversity decline are stronger in more specialized species, in line with expectations from greater demographic declines in specialist insect abundance compared to generalists (Habel et al. 2016; Warren et al. 2021; Wagner et al. 2021a). We assess genetic change by comparing museum and contemporary estimates of genetic diversity and use simulations to assess if the lag in loss of diversity observed is expected given the scale of demographic change and to what extent it lags this change. We further explore the prevalence of changes in genetic diversity in grassland butterfly species in the region with preliminary individual-based estimates of five additional related butterfly species with Swedish Red List statuses ranging from least concern to endangered (SLU Artdatabanken 2020). In the face of ongoing mass extinction, identifying species and populations at risk at an early stage of decline is paramount. Here, by combining a large set of historical and contemporary specimens, we uncover a case of ongoing genomic erosion in response to recent anthropogenic change and highlight metrics that respond quickly to population decline, providing insights into genomic erosion at its early stages when intervention is most effective.

## Results

We analyzed changes in genetic diversity, differentiation, and genomic proxies for genetic load in three focal Polyommatinae (Lepidoptera: Lycaenidae) butterfly species: *Polyommatus icarus,* a grassland generalist, *Plebejus argus*, a heathland specialist, and *Cyaniris semiargus*, a grassland specialist (Fig. 1a). We generated whole genome resequencing data from pinned museum specimens from the entomological collections of the Biological Museum at Lund University (Fägerström 2024) collected from 1917 to 1956 (Table S1; *N* = 28—*Po. icarus*; 12—*Pl. argus*; 14—*Cy. semiargus*) in southern Sweden. These individuals were sampled as populations by grouping specimens generally collected at the same locality during the same year and compared with WGS data from samples collected at corresponding localities in 2020 to 2023 by Nolen et al. (2024) (Fig. 1b; Table S1; *N* = 27—*Po. icarus*; 29—*Pl. argus*; 29—*Cy. semiargus*). As DNA extracted from historical specimens is fragmented and subject to post-mortem DNA damage (Stiller et al. 2006; Gilbert et al. 2007) (Fig. S1), we removed transitions from all analyses. As the shorter fragment size of historical samples can result in poor mapping of reads containing non-reference alleles, we constructed variation graphs for each reference genome containing high quality variants called in the modern samples. Mapping both sample types to these variation graphs reduced reference bias compared to aligning to the linear reference genome, as well as reduced the disparity in reference bias between historical and modern samples, especially when calling genotypes (Fig. S2 and S3; Supplementary Methods). The reduction in reference bias and its influence on downstream analyses is clearly visible in population structure analyses, where using the linear reference alignments resulted in samples clustering primarily by sample type (historical or modern), while using the variation graph alignments resulted in samples clustering primarily by sampling locality (Fig. S4). While the variation graph alignment method reduced reference bias in historical samples, it was not eliminated, and results should be considered with this in mind, especially for *Po. icarus* which exhibits the largest residual bias (Fig. S2 and S4). Median sequencing depth was 6.4 × (range = 2.2 to 16.7×) for historical samples and 19.3 × (range = 7.0 to 56.7×; Table S1) for modern samples. To account for variation in sequencing depth, all analyses were performed with data subsampled to between 6 and 8 × sequencing depth.

**Fig. 1.**
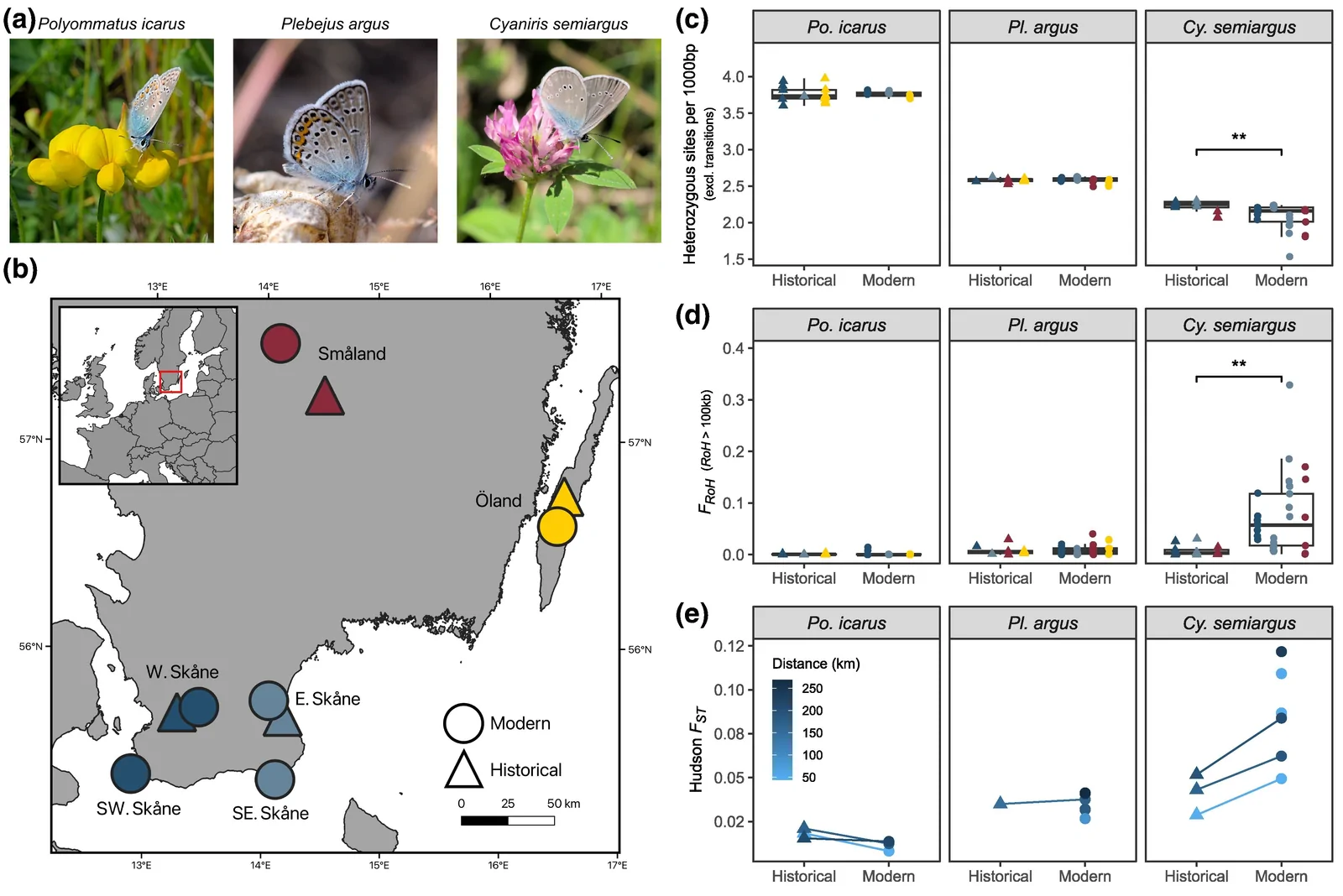
Sampling design and changes in genetic diversity, differentiation, and inbreeding in the focal species. a) Images of the three focal species. b) Map of the sampling area in southern Sweden, sampling locations are approximate. Circles represent areas with modern samples in at least one of the species and triangles represent areas with historical samples, see Table S1 for complete sampling information. c) Change in individual heterozygosity over time, measured as heterozygous sites per 1000 bp, excluding transitions. Each point represents an individual sample, grouped into historical and modern samples per species. Coloring refers to sampling region and corresponds to the map in panel B. d) Change in inbreeding coefficients over time, estimated using runs of homozygosity larger than 200 kb (*F_RoH_*). e) Change in pairwise population genetic differentiation, *F*  _ST_ over time between equivalent comparisons in each time period. Color of the points and line refer to the geographic distance between populations. Populations with no connecting line refer to pairs of modern sample populations that have no comparable historic pair. In C & D, results of linear mixed models assessing the effect of time period on the response are shown using the following scale, where panels without * are not significant (*P* > 0.05): *P≤ 0.05, **P≤ 0.01, ***P≤ 0.001.

### Reduced Genetic Diversity and Effective Population Size in Cy. Semiargus

To assess if genetic diversity has been lost over the period characterized by declines in semi-natural grasslands driven by agricultural intensification, we compared individual genome-wide heterozygosity of historical and modern samples within each species. Individual heterozygosity is significantly reduced in the specialist *Cy. semiargus* (percent difference: −6%; LMM: *P* = 0.008; Fig. 1c; Table S2 and S3), but not in *Po. icarus* or *Pl. argus*. Population specific changes in genetic diversity estimated both from mean individual heterozygosity and nucleotide diversity (Table S4) suggest that genetic diversity has mostly been reduced in populations in Skåne, where arable land cover is greatest, with only a 1% change in nucleotide diversity in Småland (Tables S5 and S6).

To understand the recent demographic patterns contributing to contemporary genetic diversity, we assessed changes in contemporary effective population size (*N_e_*) over the past century using the modern genomes with a linkage disequilibrium (LD) based approach. We find stable and very high effective population sizes in *Po. icarus* and high, but fluctuating, sizes in *Pl. argus*, in line with their maintenance of genetic diversity (Fig. S5; Table S6). In *Cy. semiargus*, we find that all populations in Skåne have gone through population declines within the past 80 years, with current effective population sizes of 88 (SE Skåne), 447 (W Skåne), and 740 (E Skåne) (Fig. S5; Table S6).

### Reduced Connectivity May Drive Loss of Genetic Diversity

While genetic diversity decline is an expected product of demographic decline, it can also be a product of reductions in gene flow (Lacy 1987). Modern *Cy. semiargus* populations exhibit stronger population structure than historical ones, based on both genetic differentiation and clustering approaches. For population pairs represented in both modern and historical samplings, modern estimates of genetic differentiation (*F*_ST_) in *Cy. semiargus* have increased from an average of 0.041 to 0.065, with the increase being largest between W Skåne and Småland (Fig. 1e; Table S7). Including population pairs in the modern sampling that are not represented in the historical, but that include similarly geographically separated populations, increases the average *F*_ST_ of modern *Cy. semiargus* pairs to 0.086. For *Po. icarus* and *Pl. argus*, average *F*_ST_ between pairs represented in both time periods has changed from 0.018 to 0.011 and 0.034 to 0.038, respectively (Fig. 1e; Table S7). When including additional modern pairs of *Pl. argus*, the modern average is slightly lower, 0.035, though this includes pairs at short distances that are not represented in historical pairs. Historical population pairs are sampled at different time points, with pairs including populations sampled up to 20 years apart (Table S7), which could influence historical *F*_ST_ estimates.

Principal component analyses (PCAs) and admixture analyses also reveal stronger population structure in modern *Cy. semiargus* samples than historical samples (Fig. 2a). Each modern population forms a distinct cluster in the specialist *Cy. semiargus*, with modern Småland samples clustering with their historical counterparts (Fig. 2b). Historical samples from Western and Eastern Skåne cluster together in a separate cluster from the corresponding modern samples, highlighting that the stronger genetic structure found in modern samples is largely driven by isolation of populations in the agricultural landscapes within Sweden's southernmost province, Skåne (Fig. 1d; Fig. S2a and b; Fig. S6). Populations of both *Po. icarus* and *Pl. argus* from Skåne separate from those sampled elsewhere in southern Sweden along the major axis of variation, with historical samples clustering with geographically proximal modern samples. Admixture analyses support only one genetic cluster in each of these species (Table S8), but the correlation of residuals suggests additional genetic clusters in *Pl. argus* (Fig. S7), consistent with the PCA. This highlights that while genetic structure likely exists, the variation is too low for reliable clustering at these sample sizes. We additionally find that historical and modern samples separate out on PC2 in *Po. icarus*, potentially an artifact of residual reference bias in historical samples of this species (Fig. S2). The general pattern of historical populations clustering in the PCA with at least one geographically proximal modern population suggests that historical samples are representative of populations that are ancestral to the modern ones.

**Fig. 2.**
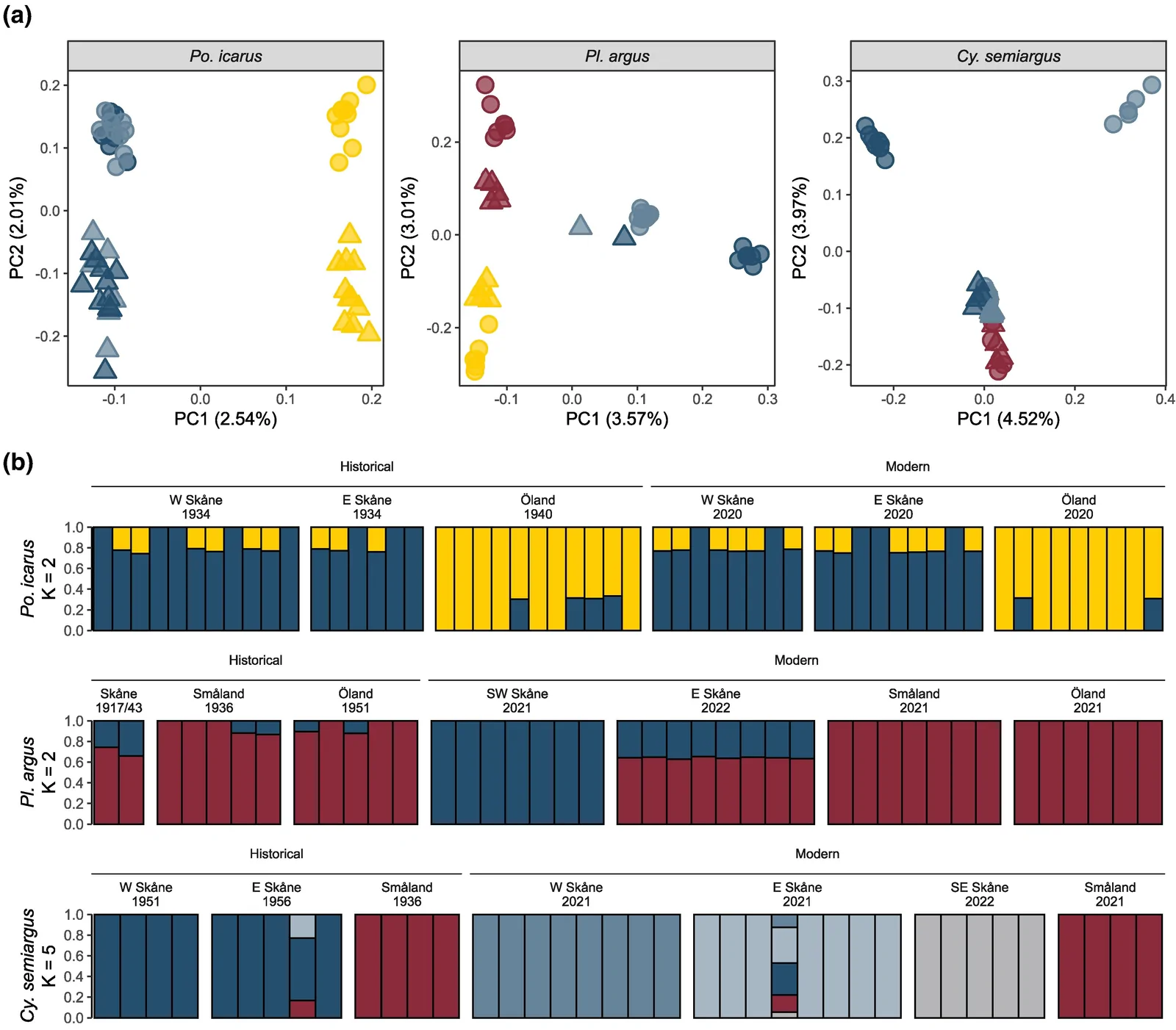
Population structure of the focal species. a) Principal Component Analysis (PCA) of the three focal species. Shapes refer to sampling period, with each circle representing a modern and each triangle a historical individual, with colors corresponding to the sampling locations in Fig. 1b. Percentages on each axis refer to the proportion of total variance explained by the component. b) Admixture analyses for the three focal species. Each color represents one of K genetic clusters, with bars representing individuals, colored by their probability of belonging to a given cluster. For *Po. icarus* and *Pl. argus*, the best fit was at K = 1, but K = 2 is shown for illustrative purposes. For *Cy. semiargus*, the best fit K = 5 is shown.

### Increased Inbreeding in Specialist Cy. Semiargus

To investigate changes in rates of inbreeding, we estimated the individual inbreeding coefficient *F_RoH_*, which is the proportion of the autosomal genome in runs of homozygosity (*RoH*) greater than a certain length (McQuillan et al. 2008). We quantified *F_RoH_* for *RoH* longer than 200 kb, corresponding roughly to *RoH* with shared ancestry over the past 180 generations (Thompson 2013). Higher estimates of *F_RoH_* here then represent a higher probability that chromosomal segments coalesce within 180 generations and can arise from genetic drift even in randomly mating populations (Kardos et al. 2016; Parreira et al. 2025). We found a significant increase in *F_RoH_* from historical to modern *Cy. semiargus* samples (difference: 0.065, LMM: *P* = 0.003; Fig. 1d; Table S9). Across species, the highest individual inbreeding coefficient for a historical sample is 0.031, with historical species averages all below 0.01 (Tables S2 and S9). In *Cy. semiargus*, modern samples have a maximum coefficient of 0.329 and an average coefficient of 0.072, an order of magnitude higher than the mean for historical samples. Though individual inbreeding coefficients included all *RoH* with an average coalescent time within 180 generations, coefficients were largely determined by *RoH* ≥ 1.5 Mb in length (Fig. 3a), corresponding coalescent times within ∼25 generations, in line with estimates of recently low effective population sizes in our demographic analyses. In *Po. icarus* and *Pl. argus*, both modern and historical estimates of *F_RoH_* are low, with averages < 0.01 and no evidence of significant increases (Table S9).

**Fig. 3.**
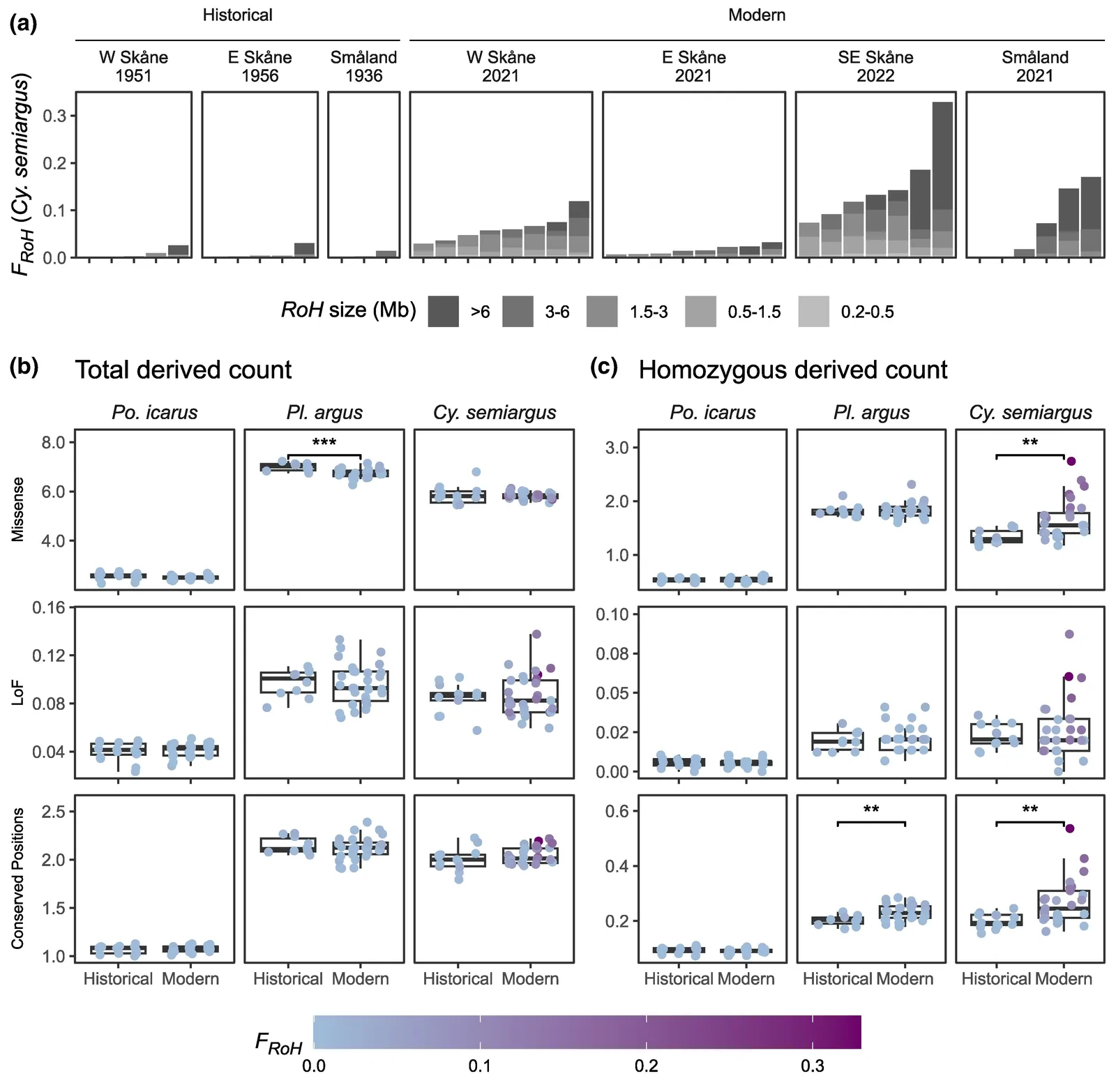
Temporal changes in derived count of putatively deleterious mutations in the focal species. (a) Individual inbreeding coefficients (*F_RoH_*) for *Cy. semiargus* samples partitioned by *RoH* size class. Longer *RoH* indicate more recent common ancestry of the identical haplotypes. b) Total count of derived alleles per 1,000 called genotypes for individuals of the three focal species at positions classifying three categories of putatively deleterious mutations: i) missense (i.e. presumed weakly deleterious; upper), ii) loss of function (LoF, i.e. presumed strongly deleterious; middle), and iii) conserved positions (presumed strongly deleterious; lower). c) Count of derived alleles in homozygous state per 1,000 called genotypes for the same mutation categories as in B. In B & C, results of linear mixed models with derived count as a response to time period are shown in each panel, where panels without * are not significant (*P* > 0.05): * *P* ≤ 0.05, ** *P* ≤ 0.01, *** *P* ≤ 0.001.

To assess if reductions in individual heterozygosity largely explained by increases in *F_RoH_* match theoretical expectations, we performed coalescent simulations parameterized on the recent demographic histories inferred from the modern genomic data, estimating individual heterozygosity and *F_RoH_* for individuals sampled at time points corresponding to our modern and historical samplings. In all simulations, reductions in genome-wide heterozygosity are primarily driven by increases in runs of homozygosity longer than the 200 kb threshold, rather than an even reduction in heterozygosity across the genome (Table S10). To assess if we observed this same pattern in the empirical genomic data, we adjusted estimates of individual heterozygosity by *F_RoH_* per individual, which approximates individual heterozygosity across the genome when *RoH*s are excluded. Individual heterozygosity excluding *RoH* is not different between time periods (Fig. S8), agreeing with the simulation predictions. For the two simulated *Cy. semiargus* populations in Skåne, the estimated ratios of modern/historical individual heterozygosity from the genomic data fall close to the median prediction of the simulations, as did average estimates of *F_RoH_* (Fig. S9).

To investigate the potential for continued loss of genetic diversity even in the absence of further demographic declines, our simulations included a neutral model-based projection of a period 100 years after the modern sampling year during which the population maintained its most recent size. In the two simulated *Cy. semiargus* populations in Skåne, the simulations projected a continued decline in heterozygosity along with increases in *F_RoH_* (Fig. S9). After the 100-year projection, median heterozygosity levels were 92.1% of historic levels in E Skåne and 84.7% in W Skåne (Fig. S9). In the case of Småland *Cy. semiargus*, we estimated a reduction in individual heterozygosity and increase in *F_RoH_* from the genomic data, but no change was projected by the simulations. As the *RoH* estimated from the modern samples suggest a recently reduced effective population size in Småland that is not captured in the inferred demographic history, this may reflect a limitation of the inferred history for this population used to parameterize the simulations.

### Increased Homozygosity of Mutations at Missense and Conserved Positions in Cy. Semiargus

Population decline and subdivision can increase the exposure of partially recessive, deleterious mutations to selection due to increased homozygosity, facilitating purging of strongly deleterious mutations. However, as populations decline and subdivide, selection weakens relative to drift, allowing weakly deleterious mutations to drift to higher frequencies and even fix, increasing genetic load (Lynch and Gabriel 1990; Lynch et al. 1995; Charlesworth 2018). Here, in the absence of fitness data, we quantified derived counts of putatively deleterious mutations as a genomic proxy for genetic load (as in e.g. van der Valk et al. 2019; Kleinman-Ruiz et al. 2022; Femerling et al. 2023; Humble et al. 2023; Dehasque et al. 2024). Using the reference genome annotations and sequence conservation scores, we considered derived alleles to be putatively deleterious if they occurred at positions where derived alleles are expected to confer some level of deleteriousness in outbred populations: (I) missense, i.e. putatively weakly deleterious; (II) loss of function (LoF), i.e. putatively strongly deleterious; and (III) conserved, i.e. with high sequence conservation across Lycaenidae, presumed to be strongly deleterious. We partitioned these counts into total derived counts and homozygous derived counts.

We find no evidence of changes in the total derived count for any of the three mutation categories in *Po. icarus* or *Cy. semiargus*. However, in *Pl. argus*, total derived count at missense positions is reduced (LMM: *P* < 0.001; Fig. 3b; Table S10), but there is no significant change in any other mutation category or at either intergenic or synonymous genic positions (Table S11). As we do not see the theoretically predicted (Wang et al. 1999; Glémin 2003) decrease in derived count at the putatively more deleterious loss of function positions in *Pl. argus*, this small reduction is likely an artifact. Homozygous derived count has increased at missense and conserved positions in *Cy. semiargus* (LMM: *P* = 0.002 and 0.006; Fig. 3c; Table S10), and at conserved positions in *Pl. argus* (LMM: *P* = 0.006; Fig. 3b; Table S10), but not changed over time in *Po. icarus.* Modern *Cyaniris semiargus* individuals with higher individual inbreeding coefficients also had higher homozygous derived counts across all categories of putatively deleterious mutations (LM: *R^2^* = 0.85, 0.36, 0.79 for missense, LoF, and conserved positions, respectively; Fig. S10). This pattern held true both at downsampled and full sequencing depth including transition variants (Fig. S10). There is also a weaker increase of total derived count with individual inbreeding coefficient at LoF and conserved positions (LM: *R^2^* = 0.12, 0.14, respectively), though these associations may be spurious, as they are not recovered at full sequencing depth (Fig. S10). There is no change in homozygous derived count at LoF positions over time in any species.

### Genetic Diversity Indicators

We utilized the genetic diversity indicator thresholds outlined by Andersson et al. (2022) to assess how detected levels of genomic erosion in the three focal species are categorized under a conservation framework. The thresholds suggest categorizations of “Acceptable”, “Warning”, and “Alarm” rates of genetic erosion, reflecting ≥95%, 75% to 94%, and <75% retention of genetic diversity over 100 years, respectively. Based on changes in mean individual heterozygosity and nucleotide diversity in each population, populations of all three species largely fall in the “Acceptable” category, except for *Cy. semiargus* in Southeast and Western Skåne, which fall into “Warning” category (Fig. 4; Tables S5 and S6). The same indicator framework in Sweden categorizes contemporary effective population sizes ≥ 500 as “Acceptable”, 51—499 as “Warning”, and ≤ 50 as “Alarm” (Johannesson and Laikre 2020; Andersson et al. 2022). Under this indicator, all *Po. icarus* and *Pl. argus* populations along with East Skåne and Småland *Cy. semiargus* would be classed as “Acceptable”. The low contemporary effective population sizes (< 500) in Southeast and West Skåne for *Cy. semiargus* would place these populations into the “Warning”, with Southeastern Skåne nearing “Alarm” (Table S6).

**Fig. 4.**
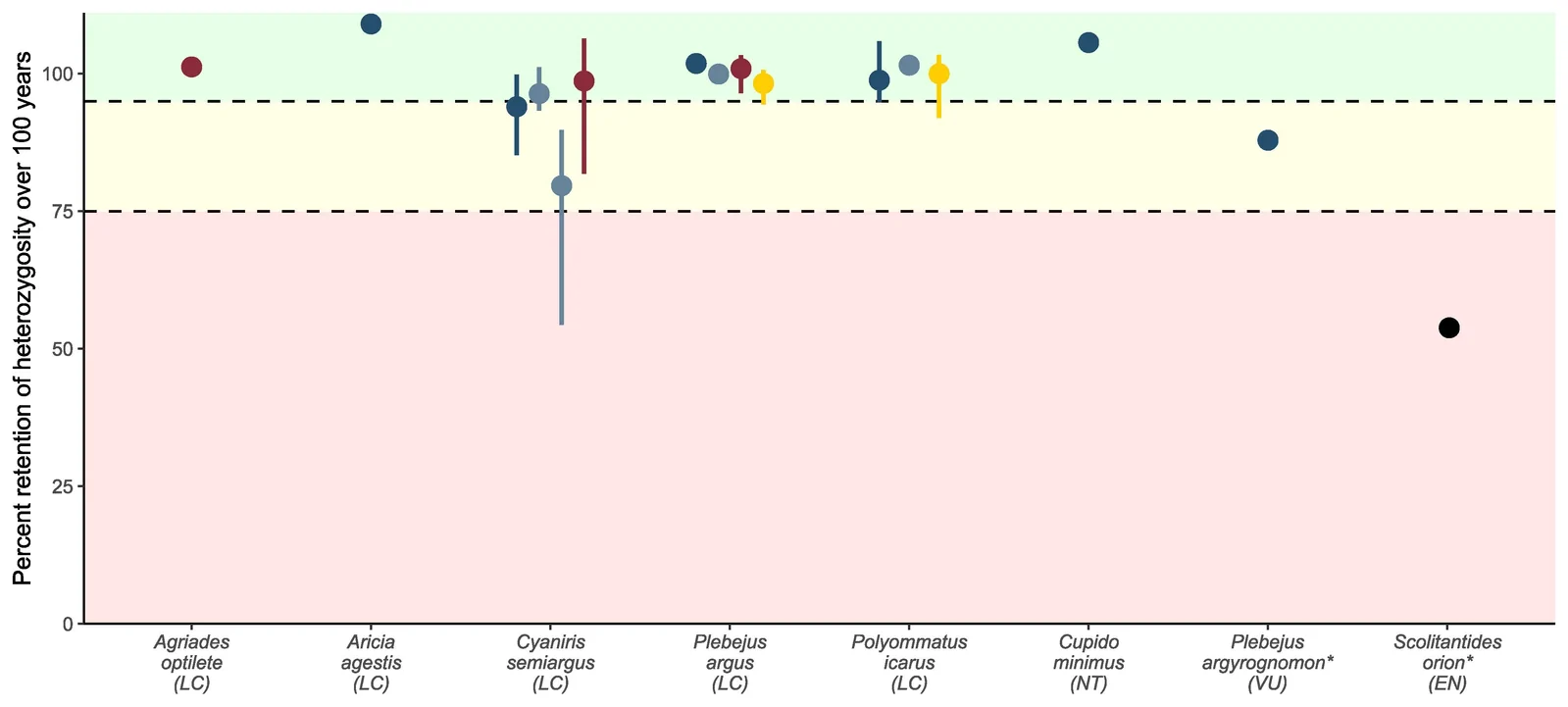
Changes in heterozygosity projected over 100 years based on the rate of change estimated from modern and historical samples for each of 8 species. Species are grouped by Swedish Red List status at the time of modern sampling, which follows the species name on the x-axis. Background coloration refers to the three genetic decline thresholds outlined by Andersson et al (2022), where a retention of ≥95% over 100 year is considered “acceptable” (green), a retention of 75% to 94% as “warning” (yellow), and a retention of <75% as “alarm” (red). Points represent median estimates for change in heterozygosity across all historical/modern individual pairs for a species. Species with bars have more than one possible sample pair, with error bars illustrating the spread of estimates across all sample pairs. *Sc. orion and *Pl. argyrognomon* include pinned museum specimens for both the modern and historical individuals. The modern individuals for *Pl. argyrognomon* and *Sc. orion* were sampled in 2003 and 2016, respectively, and the conservation statuses shown here reflect the statuses at those times. *Plebejus argyrognomon* is additionally the only species aligned to a reference genome for a different species (Table S12).

We also explored if the conservation statuses of the five additional species, ranging from “Least Concern” to “Endangered” (SLU Artdatabanken 2020) (Table S12) were associated with declines in individual heterozygosity. As a single pair of individuals were sampled from each additional species, we reassessed the three focal species, comparing all possible combinations of historical and modern individuals for each population. We find that “Least Concern” species largely show “Acceptable” changes in genetic diversity, except for Southeastern and Western Skåne *Cy. semiargus*, which fall into the “Warning” category. Of the Red Listed species, *Cupido minimus* (“Near Threatened”) falls into the “Acceptable” category, *Plebejus argyrognomon* (“Vulnerable” in 2003 when the modern individual in this study was collected; “Critically Endangered” and presumed extinct in Sweden presently (SLU Artdatabanken 2026)) into “Warning”, and *Scolitantides orion* (‘Endangered’) into “Alarm” (Fig. 4; Table S6). These declines in individual heterozygosity were associated with increases in *F_RoH_* (Table S2). We note that when using historical-modern pairs of single specimens of *Cy. semiargus*, the inferred heterozygosity decline can vary widely around the population mean, from complete maintenance to an almost 50% reduction over 100 years (Fig. 4). This illustrates that individual-based estimates may identify reductions in genetic diversity, but broader sampling is needed to confidently determine the level of genomic erosion.

## Discussion

Our analyses of 59 historical and 90 modern genomes uncover a decline in genetic diversity in the specialist grassland butterfly *Cyaniris semiargus* over the past century. The previously connected populations of this species in southern Sweden have become isolated into distinct genetic clusters. Increased rates of inbreeding have begun to increase homozygosity of putative weakly deleterious mutations in *Cy. semiargus* and inferred effective population sizes have decreased dramatically in some populations. Model-based projections forecast that genetic diversity will continue to decline even if *Cy. semiargus* populations stabilize at their current sizes, consistent with expectations that genomic erosion lags demographic change (Mualim et al. 2026; Pinto et al. 2024; Gargiulo et al. 2025). As the three focal species share a status of “Least Concern” in Sweden, our findings illustrate a case where genetic erosion assessments can identify genetic extinction debts not captured by census-based conservation assessments.

In contrast to the apparent isolation of modern *Cy. semiargus* populations, low genetic differentiation and absence of significant increases in population subdivision in *Po. icarus* and *Pl. argus* suggest gene flow maintains cohesive metapopulations in these less specialized species. While increases in genetic differentiation in *Cy. semiargus* may be driven by population decline without reductions in gene flow (Charlesworth 1998; Cruickshank and Hahn 2014), three lines of evidence suggest that currently subdivided *Cy. semiargus* populations in Skåne are subsets of a historically well-connected metapopulation. Historically lower levels of genetic differentiation and weaker clustering across southern Sweden combined with effective population size trajectories converging to similar historical sizes from distinct contemporary ones in Western and Eastern Skåne are consistent with recent isolation. The observed concurrent abrupt declines in effective population sizes, shifting estimates from those of the metapopulation to individual ones for each subpopulation, are consistent with theoretical expectations of high genetic diversity being maintained by even low levels of gene flow (Lande 1992; Nei and Takahata 1993), as documented in another Lycaenid butterfly (Gompert et al. 2021). Hence, concerns about genetic rescue (Hedrick et al. 2011) wiping out locally adapted ancestry (Hedrick and Garcia-Dorado 2016; Bell et al. 2019) are not likely to be relevant for *Cy. semiargus* and other grassland associated insects in regions with historically abundant habitat permitting dispersal between subpopulations. In general, estimates of historical population structure may prove valuable for distinguishing whether contemporary population structure reflects species ecology or is a consequence of anthropogenic habitat isolation.

The reduction of heterozygosity in *Cy. semiargus* is driven by increases in the frequency of long runs of homozygosity, demonstrating *F_RoH_* as a powerful metric to detect genomic erosion even from only contemporary samples (Kyriazis et al. 2025). Additionally, LD-based trajectories of recent effective population sizes have the potential to uncover very recent demographic declines before changes in genetic diversity is reduced. For instance, temporal changes in effective population size suggest that *Pl. argus* has experienced some very recent declines but have not experienced significant changes in individual heterozygosity or *F_RoH_*. Ultimately, while the observed reductions in genetic diversity in these species are relatively mild, our simulation analyses illustrate that such mild changes represent only a partial progression of genomic erosion, which is projected to continue even if population sizes stabilize.

Understanding to what extent genetic diversity decline reduces fitness remains a challenge, as fitness estimates require extensive sampling often not suitable for populations in decline. As we have no direct fitness estimates, we quantified counts of putatively deleterious mutations as a proxy for genetic load (Simons and Sella 2016). We found little evidence for either purging or accumulation of putatively deleterious mutations in any of the three species. This contrasts to evidence for purging and accumulation inferred using similar temporal sampling and genome annotation-based inference of putatively deleterious mutations in heavily bottlenecked species (van der Valk et al. 2019; Dussex et al. 2023; Femerling et al. 2023; Fontsere et al. 2025). This difference may be due to the populations studied here experiencing relatively more recent or lower magnitude bottlenecks, where deleterious mutations have not increased to appreciable frequencies to begin reaching fixation or be effectively purged.

However, increased rates of inbreeding in *Cy. semiargus* have increased the homozygous derived count at missense and conserved positions. Mutations at these positions are expected to include at least some partially recessive deleterious variation, where increased homozygosity would increase the realized load, reducing fitness in more inbred populations (Bertorelle et al. 2022). While not utilizing historical sampling, a similar pattern of increased homozygosity of missense mutations without changes in the total abundance of deleterious mutations after a bottleneck has been identified in unmanaged populations of scimitar-horned oryx (Humble et al. 2023). It is unclear how much small increases in the homozygosity of missense mutations might reduce fitness in cases like these, where fitness data is not available to verify the assumptions of genomic proxies for load. For example, while derived mutations at conserved positions are expected to have relatively strong deleterious effects (Cooper et al. 2005; Bertorelle et al. 2022), similar to the loss of function category, the increased homozygous derived count at conserved positions in *Cy. semiargus* more closely mirrors patterns at missense positions. Potentially, this reflects limited ability of sequence conservation scores to distinguish between weakly and strongly deleterious mutations (Huber et al. 2020; Kyriazis et al. 2023). The metric we utilize to assess sequence conservation, Genomic Evolutionary Rate Profiling (GERP) scores (Cooper et al. 2005), is based on a time tree in our study (similar to van der Valk et al. 2019; Dussex et al. 2021; von Seth et al. 2021) and hence differ from scores estimated based on neutral rates (e.g. Bortoluzzi et al. 2023). Nevertheless, our identification of conserved sites selects for the most conserved variants in each dataset and should be robust for within-species comparisons.

Recent empirical and simulation studies (Jackson et al. 2022; Pinto et al. 2024; Fontsere et al. 2025; Liu et al. 2025) have highlighted a time lag between disturbance events and genomic erosion referred to as drift debt (Gilroy et al. 2017; Dussex et al. 2023) or genetic extinction debt (Gargiulo et al. 2025). While we measure only an average 6% reduction in individual heterozygosity across the study period, we infer greater than 10-fold declines in effective population sizes in recent decades. Using coalescent simulations, we verify that this disparity is expected for such recent declines, and project a continued loss of heterozygosity to “catch up” to the scale of demographic decline for at least the next hundred generations. Importantly, such a lag is also expected for the rate of inbreeding and accumulation of genetic load (Pinto et al. 2024). Given time, while exposure of deleterious mutations may enable a portion of the load to successfully be purged, a portion may also drift to fixation, reducing mean fitness of the population in the long term (Lynch et al. 1995; Lande 1998; Grossen et al. 2020; van Oosterhout 2020; Bertorelle et al. 2022; Dussex et al. 2023). Simultaneously, the loss of diversity to drift additionally reduces adaptive potential (García-Dorado and Caballero 2021), altogether reducing the prospects of long-term persistence. Increasing the levels of gene flow to increase effective population sizes is needed to reduce this debt.

We uncovered reduced heterozygosity in two additional grassland specialist species, *Pl. argyrognomon* and *Sc. orion*, which fall under the “Warning” and “Alarm” Swedish classifications of genetic diversity decline (Andersson et al. 2022). While these individual-level estimates may not give the complete picture, the observed changes in heterozygosity overlap with those from individual pairs of *Cy. semiargus*. Reductions in heterozygosity and increases in *F_RoH_* for contemporary *Cy. semiargus* are similar to those estimated from a *Pl. argyrognomon* specimen sampled in 2003. At this point in time, *Pl. argyrognomon* was classified as “Vulnerable”, but has since gone extinct in Sweden (SLU Artdatabanken 2026). This raises concern for the future for *Cy. semiargus* in southern Sweden.

None of the three focal species are currently demographically declining at a rate to qualify for the Swedish Red List based on census estimates (SLU Artdatabanken 2020; Pettersson and Arnberg 2024). *Cyaniris semiargus* has begun to lose genetic diversity, but most genomic erosion has yet to be realized. Genetic monitoring incorporating historical samples can facilitate early identification of signals of genetic erosion to inform conservation action (Díez-del-Molino et al. 2018; Gargiulo et al. 2025). We have previously shown that lower individual heterozygosity, reflecting more abundant long *RoH*, in *Cy. semiargus* populations is associated with lower grassland area in the surrounding landscape (5 to 20 km from the local patch, Nolen et al. 2024). Grassland habitat area has declined as a result of agricultural intensification in this region (Lindborg and Eriksson 2004), and while we do not directly prove genetic erosion is a consequence of agricultural intensification, these two studies jointly suggest that current grassland extent and configuration is insufficient to maintain genetic diversity in these *Cy. semiargus* populations.

In conclusion, we document increased isolation and the early stages of genomic erosion in a specialist grassland butterfly in southern Sweden, highlighting that even formerly common grassland insects may now carry a substantial genetic extinction debt. This genetic extinction debt in specialist grassland insects may form the basis for an extinction debt at the community level (Gargiulo et al. 2025). Prompt restoration of habitat permissive to gene flow is necessary to reduce the risk of extirpation and extinction, reinforcing the need for semi-natural grassland restoration in Europe, crucial to achieving the maintenance of genetic diversity in these populations as stipulated in Goal A of the Kunming-Montreal Global Biodiversity Framework (Convention on Biological Diversity 2022). Broadly, our findings demonstrate the value of genetic monitoring in conservation assessment of insects, as early detection of genomic erosion can identify populations that appear demographically stable, but carry a genetic extinction debt, enabling timely action.

## Materials and Methods

### Study System and Sampling

To assess changes in genetic diversity, differentiation, rates of inbreeding, and deleterious mutation burden, we compared modern and historical genomes from three focal blue butterfly species (Polyommatinae), *Polyommatus icarus*, *Plebejus argus*, and *Cyaniris semiargus* (Fig. 1a). These species vary in habitat specialization, being grassland generalist, heathland specialist, and grassland specialist, respectively. We used previously generated population-level genetic data (Nolen et al. 2024) for the modern genomes. For the historical genomes, we selected pinned specimens from the entomological collections of the Biological Museum, Lund University (Fägerström 2024) sampled between 1917 to 1956 in roughly the same geographic area as the modern samples. For each historical locality, we selected 4 to 11 specimens sampled in the same year to estimate population-level metrics. The historical and modern population-level pairs were sampled in four broad geographic areas: I) Western and II) Eastern Skåne, III) Småland, and IV) Öland (Fig. 1b), with historical localities ranging approximately 12 to 40 km from their modern counterparts. This resulted in three historical-modern population pairs for *Po. icarus* and two for *Pl. argus*. For *Cy. semiargus*, we included three pairs and an additional comparison of a modern Southeastern Skåne population paired with the historical Eastern Skåne population. For *Pl. argus*, only one historical specimen was available from Western and Eastern Skåne and these individuals were compared to the closest modern populations in individual-level analyses only.

To more broadly explore the variation in changes in genetic diversity in Polyommatinae of differing conservation statuses, we sampled single historical and modern individuals of five additional species: *Agriades optilete, Aricia agestis, Cupido minimus, Plebejus argyrognomon* and *Scolitantides orion.* Modern specimens of *Ag. optilete, Ar. agestis,* and *Cu. minimus* were collected across the same geographic regions as the focal species in 2021 (Table S1). A pinned specimen used as the modern sample of *Sc. orion* was collected in 2016 in Södermanland and donated to this study by Håkan Elmqvist. Legs from a pinned specimen of *Pl. argyrognomon*, collected in 2003 in Western Skåne, were donated by Göran Engqvist and used as the modern sample for this species. Historical samples for these five species were selected to be proximal to the modern specimen sampling locality with collection dates ranging from 1939 to 1967, all provided as pinned specimens from the entomological collections of the Biological Museum, Lund University (Fägerström 2024).

### DNA Extraction and Sequencing

DNA from fresh specimens was extracted and sequenced following Nolen et al. (2024). We extracted DNA and prepared sequencing libraries from pinned museum specimens in a facility dedicated to historical DNA processing at Lund University, following the protocol described by Twort et al. (2021). Libraries prepared for pinned specimens were pooled into two equimolar pools containing 37 to 38 libraries each, sequencing each pool on a single NovaSeq 6000 S4 lane. DNA was extracted from the abdomens or legs of male specimens without homogenization to preserve anatomical structures.

### Sequence Processing

Sequence processing and analyses were largely performed using Snakemake v8.25.5 (Mölder et al. 2021), incorporating and extending the PopGLen v0.4.2 (Nolen 2025) workflow. We trimmed raw sequencing reads using fastp v0.23.4 (Chen et al. 2018) with default settings, additionally collapsing overlapping read pairs in historical samples, discarding those with less than 30 bp overlap. As historical DNA is expected to be fragmented, mapping can be biased toward reads containing the reference allele. We investigated two potential alignment options to reduce this impact: (I) BWA aln (Li and Durbin 2009) with relaxed parameters, as is used in most historical DNA studies, and (II) alignment to a variation graph incorporating known alternative variants from the modern samples, which has been shown to reduce reference biases when mapping short fragments (Martiniano et al. 2020). As we found that mapping to the variation graph reduced reference bias in both the historical and modern samples and decreased the disparity between the two types (Fig. S2 and S3), we performed all analyses with both modern and historical samples aligned to the species-specific variation graphs. Detailed methods of the processing for this comparison and the final alignment method are available in the Supplementary Methods, with a brief outline of the final method here.

To construct variation graphs for each species, we first aligned the trimmed, paired-end modern reads to the respective reference genome (Table S12) (Hayward et al. 2022; Lohse et al. 2023a; Lohse et al. 2023b) using BWA mem v0.7.17 (Li 2013), removed duplicates using Picard v2.27 (Broad Institute 2019), and realigned around indels using GATK (Van der Auwera and O’Connor 2020). We called genotypes in these samples using the BCFtools multi-allelic caller v1.21 (Danecek et al. 2021) and constructed a variation graph from these variants and the linear reference genome using vg v1.63.1 (Garrison et al. 2018). To produce the final alignments, we mapped modern and historical samples to the variant graph using vg with default setutings, additionally setting -k 15 for historical samples as recommended by Martiniano et al. (2020). We removed duplicates with Picard and dedup v0.12.8 (Peltzer et al. 2016) for modern and historical samples, respectively, and clipped overlapping read pairs using BamUtil v1.0.15 (Jun et al. 2015). We estimated post-mortem DNA damage in historical samples using DamageProfiler v1.1 (Neukamm et al. 2021).

### Genotype Calling and Filtering

To account for variation in sequencing depth, we subsampled all individuals to a mean depth of 6 × using Samtools v1.20 (Danecek et al. 2021). We excluded individuals with a mean coverage of less than 5 × from genotype calling. We implemented a sites-based filtering scheme (see Supplementary Methods) and called genotypes for each species jointly across samples using the BCFtools multi-allelic caller at sites passing the species filter sets (Table S13), including reads with minimum mapping and base qualities of 30 and 20, respectively. We kept biallelic and invariant positions, excluding those within 5 bp of indels or a variant quality less than 30. We set genotypes with a depth less than 5 and heterozygous genotypes with one allele supported by less than 20% of the reads to missing. We removed transition positions across all samples and filtered out positions with more than 30% missing data in either the historical or modern sample subsets. We additionally called and filtered genotypes using the same approach for modern *Cy. semiargus* individuals at full depth, producing sets that included and excluded transitions, to investigate the impacts of depth downsampling and transition removal on estimates of heterozygosity, *F_RoH_* (Fig. S11), and derived counts of putatively deleterious mutations (Fig. S10).

### Individual Heterozygosity, Nucleotide Diversity, and Genetic Differentiation

We estimated individual genome-wide heterozygosity for all samples from the called genotypes by dividing the count of heterozygous calls by the total count of called genotypes, including invariant sites. We estimated nucleotide diversity using Pixy v2.0.0.beta2 (Korunes and Samuk 2021), which utilizes a sample-size weighted calculation. Nucleotide diversity estimates in these populations are robust to low (N = 4) sample sizes (Nolen et al. 2024), so we included estimates for all populations with multiple individuals. We estimated this summary statistic in 50 kb non-overlapping windows for populations grouped by sampling locality, estimating the genome-wide mean across windows, and confidence intervals from 10,000 bootstraps, resampling windows with replacement. We estimated genetic differentiation using pairwise population *F*_ST_ with Pixy on the same windows with the Hudson estimator (Hudson et al. 1992), which is robust to small and variable sample sizes (Bhatia et al. 2013). We estimated mean *F*_ST_ and confidence intervals with the same approach as nucleotide diversity.

### Relatedness

As analyses of population structure can be sensitive to the presence of close relatives in the dataset, we estimated pairwise relatedness between all samples within each species dataset. We utilized the IBSrelate SFS-based method (Waples et al. 2019), as implemented in NGSrelate (Hanghøj et al. 2019), to estimate R0, R1, and KING-robust kinship coefficients (Manichaikul et al. 2010) for all pairs of individuals in the dataset. For input, we generated a Beagle file for each focal species dataset, using all sampled individuals, but subsampling individuals with higher coverage to 8 × to reduce the influence of the large variation in coverage in our modern samples. We estimated genotype likelihoods in ANGSD v0.940 (Korneliussen et al. 2014) using the GATK model from reads with a mapping quality ≥ 30, base quality ≥ 20, at positions with a minimum depth of three that passed species-specific site filters. We called SNPs with a *P*-value < 1e-6, a minor allele frequency ≥ 0.05, and with data in at least 90% of the individuals.

### Population Structure

We explored population structure using principal component analysis (PCA) as implemented in PCAngsd v1.10 (Meisner and Albrechtsen 2018). To obtain independent sites for population structure analyses, we estimated LD in the Beagle file from the relatedness analyses using ngsLD v1.2.0 (Fox et al. 2019) and pruned SNPs using prune_graph (https://github.com/fgvieira/prune_graph; v0.3.2), assuming independence of SNPs >50 kb apart and with a coefficient of correlation ($r^{2}$) < 0.1. We removed one individual from each pair of third-degree or closer relatives in the dataset (expected KING-kinship coefficient ≥ 0.0625; Table S14) before running the PCA.

We formally tested for population structure using admixture analyses for 1 to 8 ancestral clusters (K) using the pruned, relative removed Beagle file in NGSadmix v33 (Skotte et al. 2013). We performed at least 20 replicate runs for each value of K, up to 100 total runs, stopping if the three highest likelihood replicates converged within 2 log-likelihood units of each other. The “best-fit” value for K was then determined to be the highest K for which replicates met this convergence criteria, following Pečnerová et al (2021). We further assessed the model fit for each value of K using evalAdmix v0.961 (Garcia-Erill and Albrechtsen 2020).

### Runs of Homozygosity

We assessed rates of inbreeding by estimating the individual inbreeding coefficient *F_RoH_*, the proportion of the autosomal genome existing in runs of homozygosity over a specified length, incorporating runs of homozygosity >200 kb and a Phred score ≥30 in our estimates of *F_RoH_*. Per individual runs of homozygosity were estimated using BCFtools/RoH v1.21 (Narasimhan et al. 2016). We set a constant species-specific recombination rate, assuming 25 cM per autosomal chromosome based on a male-specific genetic map length of 50 cM per chromosome and a lack of recombination in females (Mackintosh et al. 2019) (Table S12), and ran the analysis on genotype calls at variable sites, setting the PL of unseen genotypes to 30 as suggested by the documentation. To ensure *F_RoH_* estimates were inferred equivalently in species with population and individual-level sampling, we did not estimate allele frequencies, instead using a default minor allele frequency of 0.4 as suggested in the documentation and ignored homozygous reference positions, as including these can inflate estimates of *F_RoH_* based on our tests (Fig. S12). We calculated *F_RoH_* for each individual by dividing the cumulative length of runs of homozygosity by the total length of the autosomes. To estimate individual heterozygosity excluding *RoH* we adjusted heterozygosity estimates using the following equation: individual heterozygosity/(1 – *F_RoH_*).

### Recent Demography and Coalescent Simulations of Heterozygosity Loss

To infer timing and magnitude of demographic declines that may contribute to reduced genetic diversity, we inferred changes in effective population size over the past 100 years from the modern samples using the LD-based approach implemented in GONE (Santiago et al. 2020). We inferred these histories in modern samples by sampling locality, as this method performs best when input samples do not have additional genetic substructure (Novo et al. 2023). We performed this analysis on the called modern variants used to construct the variation graphs, which included transitions. As we observed a frequent sudden drop in the most recent four generations in most of the GONE analyses, we visualized trajectories both including and excluding these generations (Fig. S5).

To assess if our empirical estimates of heterozygosity decline and accumulation of runs of homozygosity in *Cy. semiargus* fell within expected ranges for the characterized demographic changes, we performed coalescent simulations using Slendr (Petr et al. 2023) with the msprime (Baumdicker et al. 2022) backend. For each modern locality with a paired historical locality, we constructed demographic models that followed size changes inferred for the most recent 100 generations in the GONE analyses. As most sampled populations showed a roughly constant historical size by this time point (Fig. S5), we set the ancestral size to the estimate for 100 generations in the past. Due to the sharp drop in effective population size in the four most recent generations (Fig. S5), we fixed these generations in the model to the estimate from generation five, a more conservative estimate of the demographic decline. We additionally included a 100-year period after the modern sampling event where the population maintains its size to assess continued genomic erosion if populations were to stabilize at their current sizes. For each sampled locality, we ran 100 replicate simulations of a single, 20 Mb chromosome with a recombination rate corresponding to a map length of 25 cM, as in the *RoH* and GONE analyses, and a mutation rate of 2.9e-9 (Keightley et al. 2015). At time points corresponding to the years of collection for the historical and modern samples at a given locality and 100 years post sampling, we sampled 30 individuals from each simulation replicate and calculated mean individual heterozygosity, *F_RoH_*, and individual heterozygosity excluding *RoH* to infer a range of expected values at these time points given the demographic scenario.

### Derived Count of Putatively Deleterious Mutations

As proxies for genetic load, we estimated the burden of putatively deleterious mutations per individual using the count of derived alleles at positions where changes are expected to be deleterious. We identified such positions using two complementary methods, one based on gene annotations to infer functional effects (McLaren et al. 2016) and the other identifying positions with high sequence conservation (Davydov et al. 2010). As variants were called across the combined historical and modern sample set for each species, alleles that are fixed within one time category, but variable or fixed for the opposing allele in the other, are included.

To identify which alleles were likely ancestral, we co-inferred ancestral states and sequence conservation scores using the GenErode pipeline v0.6.2 (Kutschera et al. 2022), which estimates GERP scores (Cooper et al. 2005) from a set of outgroup species assemblies. We utilized 24 Lycaenidae chromosome-level genomes from distinct genera (Table S15) (Lohse et al. 2021; Darwin Tree of Life Consortium et al. 2021a, 2021b, 2022; Hayward et al. 2022; The Darwin Tree of Life Project Consortium 2022; Lohse et al. 2023a, 2023b; Wright et al. 2025) as outgroups, modifying the default fragment size for GenErode's alignment step from 35 to 100 bp, and using divergence times estimated with TimeTree 5 (Kumar et al. 2022). We then subset the variable sites to include only those with GERP > 0 and where the ancestral allele matched the reference to avoid underestimating derived counts in historical samples.

We identified positions where mutations altered gene function using the genome annotations provided by the Darwin Tree of Life Project (https://projects.ensembl.org/darwin-tree-of-life/). We assigned impacts to each variant using Ensembl's Variant Effect Predictor (VEP) v112.0 (McLaren et al. 2016). VEP categorizes variants into four classes: “High”, “Moderate”, “Low”, or “Modifier”, which largely correspond to loss of function, missense, synonymous, and intergenic variants. We forced VEP to pick one effect per variant, using the default selection order (McLaren et al. 2016). To identify conserved positions, we selected the top 1% of GERP scores as the most conserved positions using the get_gerp_score_percentile.py script included with GenErode. We quantified putatively deleterious burden at positions identified as loss of function (‘High” impact), missense (‘Moderate” impact), or conserved based on GERP scores, using the derived allele count at these positions. We calculated the total derived count for each individual as the count of all derived alleles for a given variant category per 1,000 called genotypes. We calculated the homozygous derived count for each individual as the count of derived alleles in homozygous state for a given variant category per 1,000 called genotypes. We additionally estimated these counts at synonymous (‘Low” impact) and intergenic positions to assess biases in derived counts across sample types.

### Categorizing Rates of Genetic Decline

To assess the changes in genetic diversity in a framework developed for conservation focused genetic monitoring, we used the indicator thresholds suggested by Andersson et al (2022), which are based upon indicators applied for national use in Sweden (Johannesson and Laikre 2020). Using these thresholds, genetic erosion within populations is measured as the amount of genetic diversity retained over a period of 100 years, assuming a constant rate of change. Three threat categories are suggested: retention ≥95% is considered “Acceptable”, 75% to 94% as “Warning”, and <75% as “Alarm”. We estimated the predicted retention of genetic diversity over 100 years for each population using individual heterozygosity and nucleotide diversity. Genetic diversity retention per year was estimated using the following equation:


$$e^{\frac{ln(H_{mod}/H_{hist})}{Y_{mod}-Y_{hist}}}$$

where *H* is the estimate of individual heterozygosity or nucleotide diversity for a given sample/population and *Y* is the sampling year for that sample/population. We estimated this metric for nucleotide diversity in the focal species using population mean estimates. For individual heterozygosity, we estimated this metric using population means in the focal species and using all pairwise comparisons of historical and modern individuals in both focal and supplemental species. For the effective population size indicator, we utilized the estimate of effective population size at generation five in the GONE analyses.

## Supplementary Material

evag235_Supplementary_Data

## Data Availability

The genetic data that support these findings will be made available upon publication at NCBI in BioProjects PRJNA1068054 (focal species, modern) & PRJNA1168917 (focal species, historical; additional species). Bioinformatic workflow configurations, extensions, and resources specific to this study are available at https://github.com/zjnolen/polyommatini-temporal-genomics and archived at https://doi.org/10.5281/zenodo.13902816.

## References

[evag235-B1] Andersson A, Karlsson S, Ryman N, Laikre L. Monitoring genetic diversity with new indicators applied to an alpine freshwater top predator. Mol Ecol. 2022:31:6422–6439. 10.1111/mec.16710. PMC10091952 36170147

[evag235-B2] Baumdicker F, et al Efficient ancestry and mutation simulation with msprime 1.0. Genetics. 2022:220:iyab229. 10.1093/genetics/iyab229. PMC9176297 34897427

[evag235-B3] Bell DA, et al The exciting potential and remaining uncertainties of genetic rescue. Trends Ecol Evol. 2019:34:1070–1079. 10.1016/j.tree.2019.06.006. 31296345

[evag235-B4] Bertorelle G, et al Genetic load: genomic estimates and applications in non-model animals. Nat Rev Genet. 2022:23:492–503. 10.1038/s41576-022-00448-x. 35136196

[evag235-B5] Bhatia G, Patterson N, Sankararaman S, Price AL. Estimating and interpreting FST: the impact of rare variants. Genome Res. 2013:23:1514–1521. 10.1101/gr.154831.113. PMC3759727 23861382

[evag235-B6] Bortoluzzi C, et al Lepidoptera genomics based on 88 chromosomal reference sequences informs population genetic parameters for conservation [preprint]. biorXiv 536868. 2023. 10.1101/2023.04.14.536868

[evag235-B7] Boyes DH, et al Is light pollution driving moth population declines? A review of causal mechanisms across the life cycle. Insect Conserv Divers. 2021:14:167–187. 10.1111/icad.12447.

[evag235-B8] Broad Institute . 2019. Picard toolkit. Broad Institute, GitHub Repository. https://broadinstitute.github.io/picard/

[evag235-B9] Charlesworth B . Measures of divergence between populations and the effect of forces that reduce variability. Mol Biol Evol. 1998:15:538–543. 10.1093/oxfordjournals.molbev.a025953. 9580982

[evag235-B10] Charlesworth B . Mutational load, inbreeding depression and heterosis in subdivided populations. Mol Ecol. 2018:27:4991–5003. 10.1111/mec.14933. 30415505

[evag235-B11] Chen S, Zhou Y, Chen Y, Gu J. Fastp: an ultra-fast all-in-one FASTQ preprocessor. Bioinformatics. 2018:34:i884–i890. 10.1093/bioinformatics/bty560. PMC6129281 30423086

[evag235-B12] Clark RD, et al Anthropocene genetic diversity loss in the marine tropics. Proc Natl Acad Sci U S A. 2025:122:e2513012122. 10.1073/pnas.2513012122. PMC12646237 41231948

[evag235-B13] Convention on Biological Diversity . Decision adopted by the conference of the parties to the convention on biological diversity 15/4. Kunming-Montreal Global Biodiversity Framework; 2022. https://www.cbd.int/doc/decisions/cop-15/cop-15-dec-04-en.pdf.

[evag235-B14] Cooper GM, et al Distribution and intensity of constraint in mammalian genomic sequence. Genome Res. 2005:15:901–913. 10.1101/gr.3577405. PMC1172034 15965027

[evag235-B15] Cruickshank TE, Hahn MW. Reanalysis suggests that genomic islands of speciation are due to reduced diversity, not reduced gene flow. Mol Ecol. 2014:23:3133–3157. 10.1111/mec.12796. 24845075

[evag235-B16] Danecek P, et al Twelve years of SAMtools and BCFtools. GigaScience. 2021:10:giab008. 10.1093/gigascience/giab008. PMC7931819 33590861

[evag235-B17] Darwin Tree of Life Consortium, et al The genome sequence of the holly blue, *Celastrina argiolus* (linnaeus, 1758) [version 1; peer review: 2 approved]. Wellcome Open Res. 2021a:6:340. 10.12688/wellcomeopenres.17478.1. PMC8729184 35028429

[evag235-B18] Darwin Tree of Life Consortium, et al The genome sequence of the green-underside blue, *Glaucopsyche alexis* (Poda, 1761) [version 1; peer review: 2 approved]. Wellcome Open Res. 2021b:6: 274. 10.12688/wellcomeopenres.17264.1. PMC10349268 37457428

[evag235-B19] Darwin Tree of Life Consortium, et al The genome sequence of the Adonis blue, *Lysandra bellargus* (Rottemburg, 1775) [version 1; peer review: 2 approved, 2 approved with reservations]. Wellcome Open Res. 2022:7:255. 10.12688/wellcomeopenres.18330.1. PMC10090861 37065725

[evag235-B20] Davydov EV, et al Identifying a high fraction of the human genome to be under selective constraint using GERP++. PLoS Comput Biol. 2010:6:e1001025. 10.1371/journal.pcbi.1001025. PMC2996323 21152010

[evag235-B21] Dehasque M, et al Temporal dynamics of woolly mammoth genome erosion prior to extinction. Cell. 2024:187:3531–3540.e13. 10.1016/j.cell.2024.05.033. 38942016

[evag235-B22] Díez-del-Molino D, et al Quantifying temporal genomic erosion in endangered Species. Trends Ecol Evol. 2018:33:176–185. 10.1016/j.tree.2017.12.002. 29289355

[evag235-B23] Dirzo R, et al Defaunation in the anthropocene. Science. 2014:345:401–406. 10.1126/science.1251817. 25061202

[evag235-B24] Dussex N, et al Population genomics of the critically endangered kākāpō. Cell Genom. 2021:1:100002. 10.1016/j.xgen.2021.100002. PMC9903828 36777713

[evag235-B25] Dussex N, et al Purging and accumulation of genetic load in conservation. Trends Ecol Evol. 2023:38:961–969. 10.1016/j.tree.2023.05.008. 37344276

[evag235-B26] Ellegren H, Galtier N. Determinants of genetic diversity. Nat Rev Genet. 2016:17:422–433. 10.1038/nrg.2016.58. 27265362

[evag235-B27] Epps CW, Keyghobadi N. Landscape genetics in a changing world: disentangling historical and contemporary influences and inferring change. Mol Ecol. 2015:24:6021–6040. 10.1111/mec.13454. 26547281

[evag235-B28] Fägerström C. 2024. Lund University Biological Museum—Insect collections Inventory. https://www.gbif.org/dataset/2356f899-6244-4bbb-aa33-50e29132e86a.

[evag235-B29] Femerling G, et al Genetic load and adaptive potential of a recovered avian Species that narrowly avoided extinction. Mol Biol Evol. 2023:40:msad256. 10.1093/molbev/msad256. PMC10701096 37995319

[evag235-B30] Fontsere C, et al Persistent genomic erosion in whooping cranes despite demographic recovery. Mol Ecol. 2025:34:e70088. 10.1111/mec.70088. PMC12684310 40856093

[evag235-B31] Fox EA, Wright AE, Fumagalli M, Vieira FG. ngsLD: evaluating linkage disequilibrium using genotype likelihoods. Bioinformatics. 2019:35:3855–3856. 10.1093/bioinformatics/btz200. 30903149

[evag235-B32] García-Dorado A, Caballero A. Neutral genetic diversity as a useful tool for conservation biology. Conserv Genet. 2021:22:541–545. 10.1007/s10592-021-01384-9.

[evag235-B33] Garcia-Erill G, Albrechtsen A. Evaluation of model fit of inferred admixture proportions. Mol Ecol Resour. 2020:20:936–949. 10.1111/1755-0998.13171. 32323416

[evag235-B34] Gargiulo R, Budde KB, Heuertz M. Mind the lag: understanding genetic extinction debt for conservation. Trends Ecol Evol. 2025:40:228–237. 10.1016/j.tree.2024.10.008. 39572352

[evag235-B35] Garrison E, et al Variation graph toolkit improves read mapping by representing genetic variation in the reference. Nat Biotechnol. 2018:36:875–879. 10.1038/nbt.4227. PMC6126949 30125266

[evag235-B36] Gauthier J, et al Museomics identifies genetic erosion in two butterfly species across the 20th century in Finland. Mol Ecol Resour. 2020:20:1191–1205. 10.1111/1755-0998.13167. PMC7540272 32304133

[evag235-B37] Gilbert MTP, et al Recharacterization of ancient DNA miscoding lesions: insights in the era of sequencing-by-synthesis. Nucleic Acids Res. 2007:35:1–10. 10.1093/nar/gkl483. PMC1802572 16920744

[evag235-B38] Gilpin ME, Soule ME. Minimum viable populations: processes of species extinction. In: Conservation biology: the science of scarcity and diversity. Sinauer Associates; 1986. p. 19–34.

[evag235-B39] Gilroy DL, Phillips KP, Richardson DS, van Oosterhout C. Toll-like receptor variation in the bottlenecked population of the Seychelles warbler: computer simulations see the ‘ghost of selection past’ and quantify the ‘drift debt’. J Evol Biol. 2017:30:1276–1287. 10.1111/jeb.13077. 28370771

[evag235-B40] Glémin S . How are deleterious mutations purged? Drift versus nonrandom mating. Evolution. 2003:57:2678–2687. 10.1111/j.0014-3820.2003.tb01512.x. 14761049

[evag235-B41] Gompert Z, et al Genomic time-series data show that gene flow maintains high genetic diversity despite substantial genetic drift in a butterfly species. Mol Ecol. 2021:30:4991–5008. 10.1111/mec.16111. 34379852

[evag235-B42] Grossen C, Guillaume F, Keller LF, Croll D. Purging of highly deleterious mutations through severe bottlenecks in alpine ibex. Nat Commun. 2020:11:1001. 10.1038/s41467-020-14803-1. PMC7035315 32081890

[evag235-B43] Habel JC, et al Butterfly community shifts over two centuries. Conserv Biol. 2016:30:754–762. 10.1111/cobi.12656. 26743786

[evag235-B44] Halsch CA, et al Insects and recent climate change. Proc Natl Acad Sci U S A. 2021:118:e2002543117. 10.1073/pnas.2002543117. PMC7812774 33431560

[evag235-B45] Hanghøj K, et al Fast and accurate relatedness estimation from high-throughput sequencing data in the presence of inbreeding. GigaScience. 2019:8:giz034. 10.1093/gigascience/giz034. PMC6488770 31042285

[evag235-B46] Hayward A, et al The genome sequence of the silver-studded blue, *Plebejus argus* (linnaeus, 1758) [version 1; peer review: 2 approved]. Wellcome Open Res. 2022:7:315. 10.12688/wellcomeopenres.18607.1. PMC9975408 36874580

[evag235-B47] Hedrick PW, Adams JR, Vucetich JA. Reevaluating and broadening the definition of genetic rescue. Conserv Biol. 2011:25:1069–1070. 10.1111/j.1523-1739.2011.01751.x. 22070251

[evag235-B48] Hedrick PW, Garcia-Dorado A. Understanding inbreeding depression, purging, and genetic rescue. Trends Ecol Evol. 2016:31:940–952. 10.1016/j.tree.2016.09.005. 27743611

[evag235-B49] Hewitt G . The genetic legacy of the quaternary ice ages. Nature. 2000:405:907–913. 10.1038/35016000. 10879524

[evag235-B50] Höglund J . The extinction vortex, is genetic variation related to extinction? In: Höglund J, editors. Evolutionary conservation genetics. Oxford University Press; 2009. p. 1–17. 10.1093/acprof:oso/9780199214211.003.0001.

[evag235-B51] Huber CD, Kim BY, Lohmueller KE. Population genetic models of GERP scores suggest pervasive turnover of constrained sites across mammalian evolution. PLoS Genet. 2020:16:e1008827. 10.1371/journal.pgen.1008827. PMC7286533 32469868

[evag235-B52] Hudson RR, Slatkin M, Maddison WP. Estimation of levels of gene flow from DNA sequence data. Genetics. 1992:132:583–589. 10.1093/genetics/132.2.583. PMC1205159 1427045

[evag235-B53] Humble E, et al Conservation management strategy impacts inbreeding and mutation load in scimitar-horned oryx. Proc Natl Acad Sci U S A. 2023:120:e2210756120. 10.1073/pnas.2210756120. PMC10160979 37098062

[evag235-B54] Ihse M . Swedish agricultural landscapes—patterns and changes during the last 50 years, studied by aerial photos. Landsc Urban Plan. 1995:31:21–37. 10.1016/0169-2046(94)01033-5.

[evag235-B55] Jackson HA, et al Genomic erosion in a demographically recovered bird species during conservation rescue. Conserv Biol. 2022:36:e13918. 10.1111/cobi.13918. PMC9546124 35554972

[evag235-B56] Johannesson K, Laikre L. Monitoring of genetic diversity in environmental monitoring. Report to the Swedish Agency for Marine and Water Management (dnr HaV 3642-2018, 3643-2018). 2020.

[evag235-B57] Jun G, Wing MK, Abecasis GR, Kang HM. An efficient and scalable analysis framework for variant extraction and refinement from population-scale DNA sequence data. Genome Res. 2015:25:918–925. 10.1101/gr.176552.114. PMC4448687 25883319

[evag235-B58] Kardos M, et al Genomics advances the study of inbreeding depression in the wild. Evol Appl. 2016:9:1205–1218. 10.1111/eva.12414. PMC5108213 27877200

[evag235-B59] Keightley PD, et al Estimation of the spontaneous mutation rate in heliconius melpomene. Mol Biol Evol. 2015:32:239–243. 10.1093/molbev/msu302. PMC4271535 25371432

[evag235-B60] Kleinman-Ruiz D, et al Purging of deleterious burden in the endangered Iberian lynx. Proc Natl Acad Sci U S A. 2022:119:e2110614119. 10.1073/pnas.2110614119. PMC8931242 35238662

[evag235-B61] Korneliussen TS, Albrechtsen A, Nielsen R. ANGSD: analysis of next generation sequencing data. BMC Bioinformatics. 2014:15:1–13. 10.1186/s12859-014-0356-4. PMC4248462 25420514

[evag235-B62] Korunes KL, Samuk K. Pixy: unbiased estimation of nucleotide diversity and divergence in the presence of missing data. Mol Ecol Resour. 2021:21:1359–1368. 10.1111/1755-0998.13326. PMC8044049 33453139

[evag235-B63] Kumar S, et al TimeTree 5: an expanded resource for Species divergence times. Mol Biol Evol. 2022:39:msac174. 10.1093/molbev/msac174. PMC9400175 35932227

[evag235-B64] Kutschera VE, et al GenErode: a bioinformatics pipeline to investigate genome erosion in endangered and extinct species. BMC Bioinformatics. 2022:23:228. 10.1186/s12859-022-04757-0. PMC9195343 35698034

[evag235-B65] Kyriazis CC, Robinson JA, Lohmueller KE. Using computational simulations to model deleterious variation and genetic load in natural populations. Am Nat. 2023:202:737–752. 10.1086/726736. PMC10897732 38033186

[evag235-B66] Kyriazis CC, Robinson JA, Lohmueller KE. Long runs of homozygosity are reliable genomic markers of inbreeding depression. Trends Ecol Evol. 2025:40:874–884. 10.1016/j.tree.2025.06.013. PMC13238715 40752972

[evag235-B67] Lacy RC . Loss of genetic diversity from managed populations: interacting effects of drift, mutation, immigration, selection, and population subdivision. Conserv Biol. 1987:1:143–158. 10.1111/j.1523-1739.1987.tb00023.x.

[evag235-B68] Lande R . Neutral theory of quantitative genetic variance in an island model with local extinction and colonization. Evolution. 1992:46:381–389. 10.1111/j.1558-5646.1992.tb02046.x. 28564025

[evag235-B69] Lande R . Risk of population extinction from fixation of new deleterious mutations. Evolution. 1994:48:1460–1469. 10.1111/j.1558-5646.1994.tb02188.x. 28568413

[evag235-B70] Lande R . Mutation and conservation. Conserv Biol. 1995:9:782–791. 10.1046/j.1523-1739.1995.09040782.x.

[evag235-B71] Lande R . Risk of population extinction from fixation of deleterious and reverse mutations. Genetica. 1998:102:21–27. 10.1023/A:1017018405648. 9766960

[evag235-B72] Lande R, Shannon S. The role of genetic variation in adaptation and population persistence in a changing environment. Evolution. 1996:50:434–437. 10.1111/j.1558-5646.1996.tb04504.x. 28568879

[evag235-B73] Li H . Aligning sequence reads, clone sequences and assembly contigs with BWA-MEM [preprint]. arXiv. 2013:3997. 10.48550/arXiv.1303.3997.

[evag235-B74] Li H, Durbin R. Fast and accurate short read alignment with burrows–wheeler transform. Bioinformatics. 2009:25:1754–1760. 10.1093/bioinformatics/btp324. PMC2705234 19451168

[evag235-B75] Lindborg R, Eriksson O. Historical landscape connectivity affects present plant Species diversity. Ecology. 2004:85:1840–1845. 10.1890/04-0367.

[evag235-B76] Liu X, et al Time-lagged genomic erosion and future environmental risks in a bird on the brink of extinction. Proc R Soc Lond B Biol Sci. 2025:292:20242480. 10.1098/rspb.2024.2480. PMC11936686 40132633

[evag235-B77] Lohse K, et al The genome sequence of the small copper, *Lycaena phlaeas* (linnaeus, 1760) [version 1; peer review: 2 approved with reservations]. Wellcome Open Res. 2021:6:294. 10.12688/wellcomeopenres.17289.1.

[evag235-B78] Lohse K, et al The genome sequence of the mazarine blue, *Cyaniris semiargus* (rottemburg, 1775) [version 1; peer review: 2 approved]. Wellcome Open Res. 2023a:8:181. 10.12688/wellcomeopenres.19362.1. PMC11109581 38779052

[evag235-B79] Lohse K, et al The genome sequence of the common blue, *Polyommatus icarus* (rottemburg, 1775) [version 1; peer review: 2 approved, 1 approved with reservations]. Wellcome Open Res. 2023b:8:72. 10.12688/wellcomeopenres.18772.1. PMC11824579 39949385

[evag235-B80] Lynch M, Conery J, Burger R. Mutation accumulation and the extinction of small populations. Am Nat. 1995:146:489–518. 10.1086/285812.

[evag235-B81] Lynch M, Gabriel W. Mutation load and the survival of small populations. Evolution. 1990:44:1725–1737. 10.1111/j.1558-5646.1990.tb05244.x. 28567811

[evag235-B82] Mackintosh A, et al The determinants of genetic diversity in butterflies. Nat Commun. 2019:10:3466. 10.1038/s41467-019-11308-4. PMC6672018 31371715

[evag235-B83] Manichaikul A, et al Robust relationship inference in genome-wide association studies. Bioinformatics. 2010:26:2867–2873. 10.1093/bioinformatics/btq559. PMC3025716 20926424

[evag235-B84] Martiniano R, et al Removing reference bias and improving indel calling in ancient DNA data analysis by mapping to a sequence variation graph. Genome Biol. 2020:21:250. 10.1186/s13059-020-02160-7. PMC7499850 32943086

[evag235-B85] McLaren W, et al The ensembl variant effect predictor. Genome Biol. 2016:17:122. 10.1186/s13059-016-0974-4. PMC4893825 27268795

[evag235-B86] McQuillan R, et al Runs of homozygosity in European populations. Am J Hum Genet. 2008:83:359–372. 10.1016/j.ajhg.2008.08.007. PMC2556426 18760389

[evag235-B87] Meisner J, Albrechtsen A. Inferring population structure and admixture proportions in low-depth NGS data. Genetics. 2018:210:719–731. 10.1534/genetics.118.301336. PMC6216594 30131346

[evag235-B88] Mölder F, et al Sustainable data analysis with snakemake [version 2; peer review: 2 approved]. F1000Res. 2021:10:33. 10.12688/f1000research.29032.2. PMC8114187 34035898

[evag235-B89] Mualim KS, et al Large future genetic diversity losses are predicted from conservation indicators even with habitat protection. Proceedings of the National Academy of Sciences. 2026:123:e2514371123. 10.1073/pnas.2514371123. PMC13037886 41886371

[evag235-B90] Narasimhan V, et al BCFtools/RoH: a hidden markov model approach for detecting autozygosity from next-generation sequencing data. Bioinformatics. 2016:32:1749–1751. 10.1093/bioinformatics/btw044. PMC4892413 26826718

[evag235-B91] Nei M, Takahata N. Effective population size, genetic diversity, and coalescence time in subdivided populations. J Mol Evol. 1993:37:240–244. 10.1007/BF00175500. 8230248

[evag235-B92] Neukamm J, Peltzer A, Nieselt K. DamageProfiler: fast damage pattern calculation for ancient DNA. Bioinformatics. 2021:37:3652–3653. 10.1093/bioinformatics/btab190. 33890614

[evag235-B93] Nolen ZJ . PopGLen—a snakemake pipeline for performing population genomic analyses using genotype likelihood-based methods. Bioinformatics. 2025:41:btaf105. 10.1093/bioinformatics/btaf105. PMC11932725 40067089

[evag235-B94] Nolen ZJ, Rundlöf M, Runemark A. Species-specific erosion of genetic diversity in grassland butterflies depends on landscape land cover. Biol Conserv. 2024:296:110694. 10.1016/j.biocon.2024.110694.

[evag235-B95] Novo I, et al Impact of population structure in the estimation of recent historical effective population size by the software GONE. Genet Sel Evol. 2023:55:86. 10.1186/s12711-023-00859-2. PMC10694967 38049712

[evag235-B96] O’Brien D, et al Bringing together approaches to reporting on within species genetic diversity. Journal of Applied Ecology. 2022:59:2227–2233. 10.1111/1365-2664.14225.

[evag235-B97] Parejo M, et al Digging into the genomic past of Swiss honey bees by whole-genome sequencing museum specimens. Genome Biol Evol. 2020:12:2535–2551. 10.1093/gbe/evaa188. PMC7720081 32877519

[evag235-B98] Parreira BR, et al Q&A: inbreeding and its implications for conservation. BMC Biol. 2025:23:314. 10.1186/s12915-025-02386-6. PMC12538986 41121152

[evag235-B99] Pečnerová P, et al High genetic diversity and low differentiation reflect the ecological versatility of the African leopard. Curr Biol. 2021:31:1862–1871.e5. 10.1016/j.cub.2021.01.064. 33636121

[evag235-B100] Peltzer A, et al EAGER: efficient ancient genome reconstruction. Genome Biol. 2016:17:1–14. 10.1186/s13059-016-0918-z. PMC4815194 27036623

[evag235-B101] Petr M, Haller BC, Ralph PL, Racimo F. Slendr: a framework for spatio-temporal population genomic simulations on geographic landscapes. Peer Community J. 2023:3:e121. 10.24072/pcjournal.354. PMC11233137 38984034

[evag235-B102] Pettersson LB, Arnberg H. Svensk dagfjärilsövervakning, årsrapport för 2023. Biologiska institutionen, Lunds universitet; 2024. https://www.dagfjarilar.lu.se/sites/default/files/public/pdf/sebms_2023_korr.pdf.

[evag235-B103] Pinto AV, et al The impact of habitat loss and population fragmentation on genomic erosion. Conserv Genet. 2024:25:49–57. 10.1007/s10592-023-01548-9.

[evag235-B104] Raven PH, Wagner DL. Agricultural intensification and climate change are rapidly decreasing insect biodiversity. Proc Natl Acad Sci U S A. 2021:118:e2002548117. 10.1073/pnas.2002548117. PMC7812793 33431564

[evag235-B105] Romiguier J, et al Comparative population genomics in animals uncovers the determinants of genetic diversity. Nature. 2014:515:261–263. 10.1038/nature13685. 25141177

[evag235-B106] Santiago E, et al Recent demographic history inferred by high-resolution analysis of linkage disequilibrium. Mol Biol Evol. 2020:37:3642–3653. 10.1093/molbev/msaa169. 32642779

[evag235-B107] Schwartz MK, Luikart G, Waples RS. Genetic monitoring as a promising tool for conservation and management. Trends Ecol Evol. 2007:22:25–33. 10.1016/j.tree.2006.08.009. 16962204

[evag235-B108] Seibold S, et al Arthropod decline in grasslands and forests is associated with landscape-level drivers. Nature. 2019:574:671–674. 10.1038/s41586-019-1684-3. 31666721

[evag235-B109] Shipley JR, et al Agricultural practices and biodiversity: conservation policies for semi-natural grasslands in Europe. Curr Biol. 2024:34:R753–R761. 10.1016/j.cub.2024.06.062. 39163831

[evag235-B110] Simons YB, Sella G. The impact of recent population history on the deleterious mutation load in humans and close evolutionary relatives. Curr Opin Genet Dev. 2016:41:150–158. 10.1016/j.gde.2016.09.006. PMC5161708 27744216

[evag235-B111] Skotte L, Korneliussen TS, Albrechtsen A. Estimating individual admixture proportions from next generation sequencing data. Genetics. 2013:195:693–702. 10.1534/genetics.113.154138. PMC3813857 24026093

[evag235-B112] SLU Artdatabanken . Rödlistade arter i sverige 2020. SLU Artdatabanken; 2020.

[evag235-B113] SLU Artdatabanken . 2026. Artfakta: kronärtsblåvinge (*Plebejus argyrognomon*). https://artfakta.se/taxa/101598.

[evag235-B114] Stiller M, et al Patterns of nucleotide misincorporations during enzymatic amplification and direct large-scale sequencing of ancient DNA. Proc Natl Acad Sci U S A. 2006:103:13578–13584. 10.1073/pnas.0605327103. PMC1564221 16938852

[evag235-B115] The Darwin Tree of Life Project Consortium . Sequence locally, think globally: the darwin tree of life project. Proc Natl Acad Sci U S A. 2022:119:e2115642118. 10.1073/pnas.2115642118. PMC8797607 35042805

[evag235-B116] Theissinger K, et al How genomics can help biodiversity conservation. Trends Genet. 2023:39:545–559. 10.1016/j.tig.2023.01.005. 36801111

[evag235-B117] Thompson EA . Identity by descent: variation in meiosis, across genomes, and in populations. Genetics. 2013:194:301–326. 10.1534/genetics.112.148825. PMC3664843 23733848

[evag235-B118] Twort VG, Minet J, Wheat CW, Wahlberg N. Museomics of a rare taxon: placing whalleyanidae in the lepidoptera tree of life. Syst Entomol. 2021:46:926–937. 10.1111/syen.12503.

[evag235-B119] Van der Auwera GA, O’Connor BD. Genomics in the cloud: using docker, GATK, and WDL in terra. O’Reilly Media, Inc; 2020.

[evag235-B120] van der Valk T, et al Historical genomes reveal the genomic consequences of recent population decline in eastern gorillas. Curr Biol. 2019:29:165–170.e6. 10.1016/j.cub.2018.11.055. 30595519

[evag235-B121] van Klink R, et al Meta-analysis reveals declines in terrestrial but increases in freshwater insect abundances. Science. 2020:368:417–420. 10.1126/science.aax9931. 32327596

[evag235-B122] van Oosterhout C . Mutation load is the spectre of species conservation. Nat Ecol Evol. 2020:4:1004–1006. 10.1038/s41559-020-1204-8. 32367032

[evag235-B123] von Seth J, et al Genomic insights into the conservation status of the world's last remaining sumatran rhinoceros populations. Nat Commun. 2021:12:2393. 10.1038/s41467-021-22386-8. PMC8071806 33896938

[evag235-B124] Wagner DL, Fox R, Salcido DM, Dyer LA. A window to the world of global insect declines: moth biodiversity trends are complex and heterogeneous. Proc Natl Acad Sci U S A. 2021a:118:e2002549117. 10.1073/pnas.2002549117. PMC7812805 33431565

[evag235-B125] Wagner DL, Grames EM, Forister ML, Berenbaum MR, Stopak D. Insect decline in the anthropocene: death by a thousand cuts. Proc Natl Acad Sci U S A. 2021b:118:e2023989118. 10.1073/pnas.2023989118. PMC7812858 33431573

[evag235-B126] Wang J, Hill WG, Charlesworth D, Charlesworth B. Dynamics of inbreeding depression due to deleterious mutations in small populations: mutation parameters and inbreeding rate. Genet Res (Camb). 1999:74:165–178. 10.1017/S0016672399003900. 10584559

[evag235-B127] Waples RK, Albrechtsen A, Moltke I. Allele frequency-free inference of close familial relationships from genotypes or low-depth sequencing data. Mol Ecol. 2019:28:35–48. 10.1111/mec.14954. PMC6850436 30462358

[evag235-B128] Warren MS, et al The decline of butterflies in Europe: problems, significance, and possible solutions. Proc Natl Acad Sci U S A. 2021:118:e2002551117. 10.1073/pnas.2002551117. PMC7812787 33431566

[evag235-B129] Webster MT, Beaurepaire A, Neumann P, Stolle E. Population genomics for insect conservation. Annu Rev Anim Biosci. 2023:11:115–140. 10.1146/annurev-animal-122221-075025. 36375448

[evag235-B130] Wright CJ, et al Project psyche: reference genomes for all lepidoptera in Europe. Trends Ecol Evol. 2025:40:1234–1250. 10.1016/j.tree.2025.10.007. 41309387

