# Supplementary material for: Species-specific Loss of Genetic Diversity and Inbreeding Following Agricultural Intensification
Source: Genome Biol Evol. 2026 Sep 24;18(11):evag235. doi: 10.1093/gbe/evag235 (PMC13750294; doi:10.1093/gbe/evag235)
Supplement: Supplementary file 1 [file evag235_supplementary_data.pdf]

Supplementary Material for
Species-specific loss of genetic diversity and inbreeding following agricultural intensification

Zachary J. Nolen[1*], Patrycja Jamelska[1], Ana Sofia Torres Lara[1,2], Niklas Wahlberg[1], Anna Runemark[1]

[1] Department of Biology, Lund University, Lund, Sweden 22362
[2] Faculty 2 Biology/Chemistry, University of Bremen, Bremen, Germany 28359

*Corresponding author: Zachary J. Nolen

Email: zachary.nolen@biol.lu.se

Supplementary Methods

Sites based filtering scheme. We utilized a sites-based filtering scheme similar to that implemented by Pečnerová et al (2021) which intersects multiple independent sets of filtered sites to create a global list of filtered sites. We performed this filtering for each species separately. We excluded sex-linked chromosomes, mitochondria, and scaffolds less than 1 Mb in length. We excluded repeats based on the softmasked reference genomes provided by Ensembl and the Darwin Tree of Life Project (The Darwin Tree of Life Project Consortium 2022; https://projects.ensembl.org/darwin-tree-of-life/). For species with no softmasked genome available, we identified repeats using RepeatModeler (Flynn et al. 2020) and RepeatMasker (Smit et al. 2013). We estimated positions in the lower and upper 1% of global sequencing depth distributions separately across all samples, historical samples, and modern samples, excluding sites that appeared in any of the three sets. The number and proportion of sites passing each of these filters, as well as passing the final intersected filter set, are reported for each species in Table S13.

Investigating different alignment options to reduce reference bias. Higher reference bias is expected in historical DNA as shorter fragments lead to those containing reference alleles to be favored over those with alternates in the alignment process (Dolenz et al. 2024). These concerns are especially relevant to studies which aim to compare estimates between degraded historical samples and high-quality contemporary ones, where different average sequence length can lead to differences in reference bias that drive the different signals in the historical and contemporary samples. We investigated the amount of reference bias and the impact on downstream analyses under different alignment strategies. The first strategy uses bwa aln (Li and Durbin 2009) with relaxed edit distance and gap open parameters (Kircher 2012; Schubert et al. 2012), the most common technique in historical DNA studies to improve alternative allele mapping (hereafter referred to as 'bwa aln'). The second augments the reference genome into a variation graph with information on known alternative variants identified in modern individuals, improving the ability of shorter fragments to map alternative alleles (Martiniano et al. 2020) (hereafter referred to as 'vg').

To generate the dataset for alignment with bwa aln, we aligned each sample to the corresponding reference genome (Hayward et al. 2022; Lohse, Wellcome Sanger Institute Tree of Life programme, et al. 2023; Lohse, Hayward, et al. 2023) using the steps in the PopGLen pipeline v0.4.1. This first trims adapters and poly-G tails using fastp v0.23.4 (Chen et al. 2018), keeping reads as paired in modern samples and collapsing overlapping reads in historical samples with a 30bp overlap, discarding reads < 30 bp from both after trimming and collapsing. We then mapped modern samples to the references with bwa mem v0.7.18 (Li 2013) using default settings and collapsed reads from historical samples with bwa aln v0.7.18, disabling seeding (-l 16500) decreasing the fraction of missing alignments (-n) from the default of 0.04 to 0.01 and increasing the number of gap opens (-o) form 1 to 2. We additionally disabled seeding (-k 16500) due to the expectation that the first bases of reads may have DNA damage. After alignment, we removed duplicates with Picard v3.2.0 (Broad Institute 2019) in modern samples, setting the optical duplicate pixel distance to 2500, and using dedup v0.12.8 (Peltzer et al. 2016) in historical samples. We realigned around indels in both modern and historical samples using the GATK IndelRealigner v3.8 (Van der Auwera and O'Connor 2020). As ANGSD (Korneliussen et al. 2014) counts overlapping paired reads twice (Lou et al. 2021), we clipped overlapping reads in the modern samples with BamUtil v1.0.15 (Jun et al. 2015).

To generate the dataset for alignment with vg, we joint called genotypes across the modern samples of each species aligned with bwa mem using the BCFtools multi-allelic caller v1.21 (Danecek et al. 2021). We called genotypes with a mapping and base quality filter of 30, excluded positions in the upper percentile of the global depth distribution, and grouped samples by sampling locality for the built-in Hardy Weinberg equilibrium assumption (-G). We filtered out genotypes with a genotype quality < 30 or depth < 6 and filtered positions to biallelic SNPs, removing those within 5 bp of indels, a quality < 30, a minor allele frequency < 0.05, or missing data in > 40% of the samples after genotype filtering. We then constructed a variation graph for each species from the linear reference genome and the filtered variants VCF using vg v1.63.1 (Garrison et al. 2018). To generate the final modern sample alignments, we re-aligned the trimmed modern reads to variation graph using vg with default settings, re-ordered scaffolds to match the reference and removed duplicates with Picard and clipped overlapping reads with BamUtil.

To generate the final historical sample alignments, we aligned the collapsed reads using vg, setting a minimum mem length of 15 (-k 15) and set the band width for long reads to a size greater than the largest fragments included (-w 300) as recommended by Martiniano et al. (2020).

To estimate reference bias, we utilized the identity by state distance to the reference averaged across sites. We made this estimate using single read sampling at each position as well as from called genotypes. We estimated single read sampling IBS distance per sample using ANGSD using the settings implemented in PopGLen to randomly sample a single read (-doIBS 1) at each position passing the filtered sites set, removing transitions (-rmTrans 1), including only sites with at least three reads (-setMinDepth 3) and only using reads with a minimum base quality of 20 and mapping quality of 30. To estimate the identity by state distance for called genotypes, we called and filtered genotypes from the bwa aln and vg alignments separately, following the methods described in the main manuscript, to produce comparable BCFs. Identity by state similarity to the reference was estimated per individual as the count of called reference alleles divided by 2 times the count of called genotypes.

We found that using vg reduced reference bias for both modern and historical samples compared to bwa aln, though this effect was larger for historical samples (Figure S2). This effect was species-specific, with the largest improvement in *Po. icarus*, followed by *Pl. argus*, then by *Cy. semiargus*. This pattern follows patterns of heterozygosity in these three species (Nolen et al. 2024), reflecting that disparities in reference bias between historical and modern samples are likely more pronounced in species with higher heterozygosity. This has implications for other population genomic studies working with historical insect genomes, as higher levels of heterozygosity in insects compared to vertebrates (Webster et al. 2023) may mean that standard alignment methods do not sufficiently reduce reference bias in these organisms to produce reliable downstream results.

To understand better how the mismatch rates of the different aligners changed with fragment size, we plotted average mismatch rates per fragment size for a *Po. icarus* historical sample using AMBER (Dolenz et al. 2024), including only fragments with mapping qualities >= 30. We did this for alignments with bwa aln and vg with the settings described above. We found that under all three tested bwa aln settings, mismatch rates were generally lower than with vg, and that an even pattern of mismatches across the majority of fragment sizes was not achieved even with the most permissive bwa aln settings we tested (i.e. -n 0.01 -o 2; Figure S3). While the pattern of mismatch rates is more even across fragment sizes for vg, we still see a reduced mismatch rate for smaller fragments of sizes that make up a considerable portion of the fragment distribution (Figure S3), which likely explains why some reference bias in the historical samples still persists when using this aligner.

Additionally, to illustrate the impact of reference bias on downstream analyses, we performed principal component analyses (PCAs) for each of the three species when aligned with bwa aln and aligned with vg (Figure S4). We found that samples clustered primarily by sampling time period when aligned with bwa aln, likely due to reference biases shifting allele frequencies in the historical samples towards the reference allele. When aligned with vg, historical samples clustered instead with modern counterparts sampled in nearby localities, as is expected biologically. In *Po. icarus*, where all population genetic structure is captured in PC1, we still observe some clustering by sampling time period along PC2 when aligned with vg, potentially reflecting the greater residual reference bias in this species.

Given the more even mismatch rate across fragment sizes and generally lower disparity of mismatch rates when using vg compared to bwa aln, we chose to perform analyses with alignments made with vg for the main manuscript. As filtered genotype calls further showed reductions in reference bias compared to randomly sampled reads, we additionally chose to utilize genotype calls for the majority of analyses rather than genotype likelihoods.

Supplementary Figures and Tables

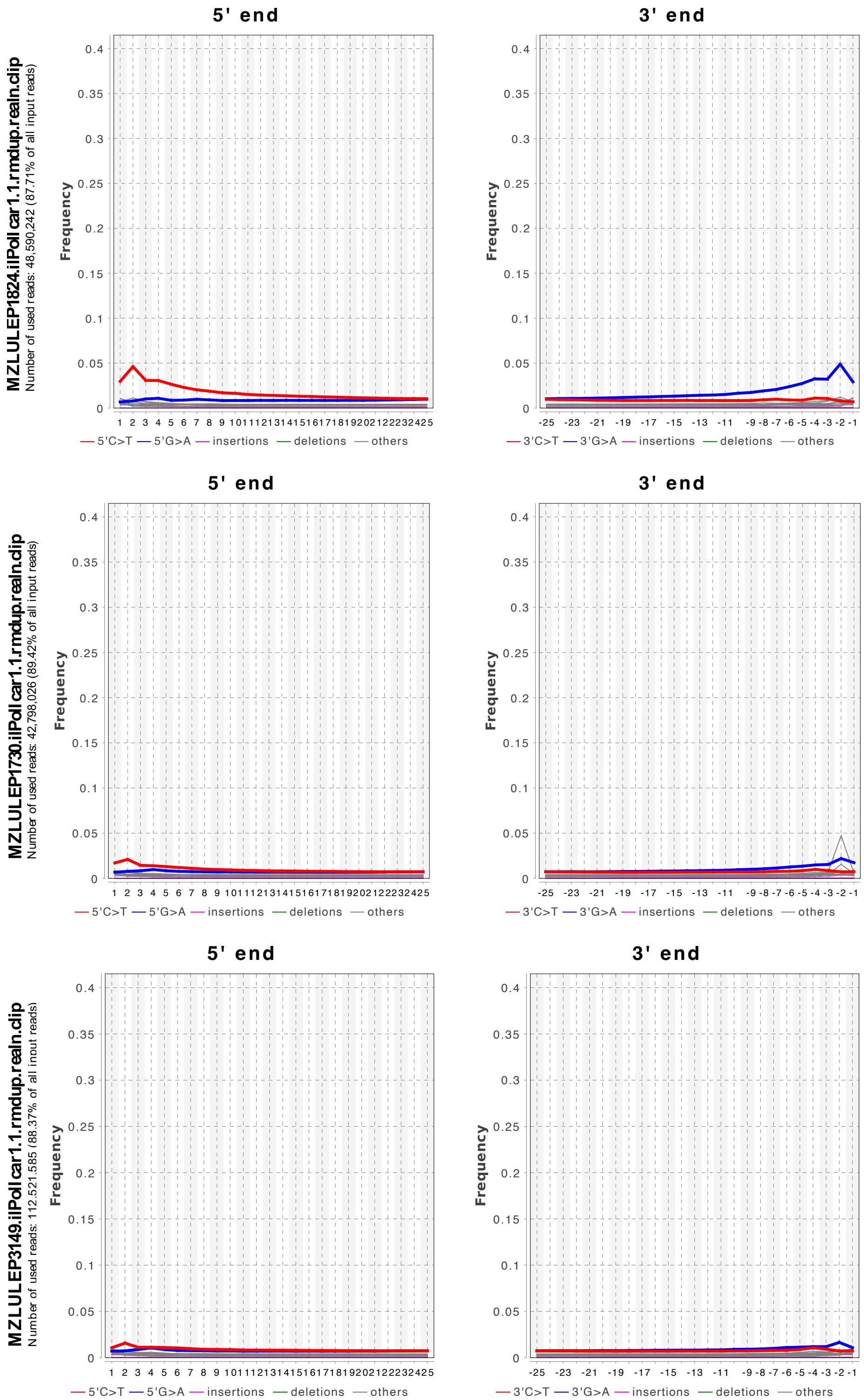

Figure S1. Examples of DNA damage profiles for historical samples. Damage profiles produced by DamageProfiler (Neukamm et al. 2021) for the samples with the greatest (upper), median (middle), and least (lower) DNA damage in the focal species dataset are shown. In all analyses, we accounted for these misincorporations by removing transition variants.

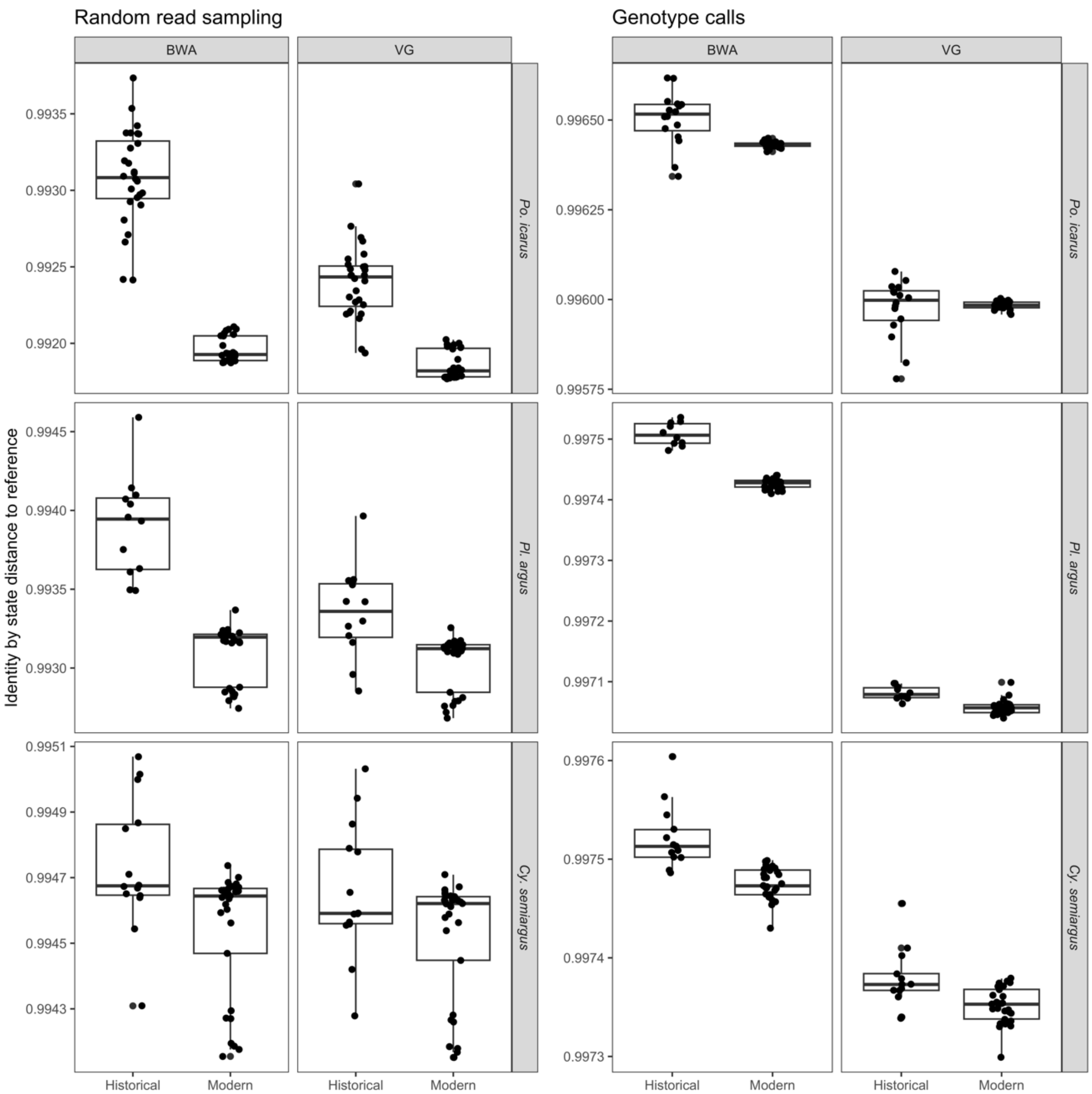

Figure S2. Examining effects of alignment method on reference bias. As historical samples are expected to experience reference biases due to shorter fragment size compared to modern samples, we assessed the impact of alignment method on reference bias, comparing alignment to a linear reference with bwa with relaxed parameters to alignment to a variation graph incorporating known variants from modern samples with vg. Reference bias of the two alignment methods is shown here for historical and modern samples of the three focal species, estimated both by sampling a random read at all positions with >= 3x depth (left panels) and from called genotypes (right panels). We find that the variation graph reduced the disparity in reference bias between historical and modern samples, selecting this alignment method to move forward with for downstream analyses.

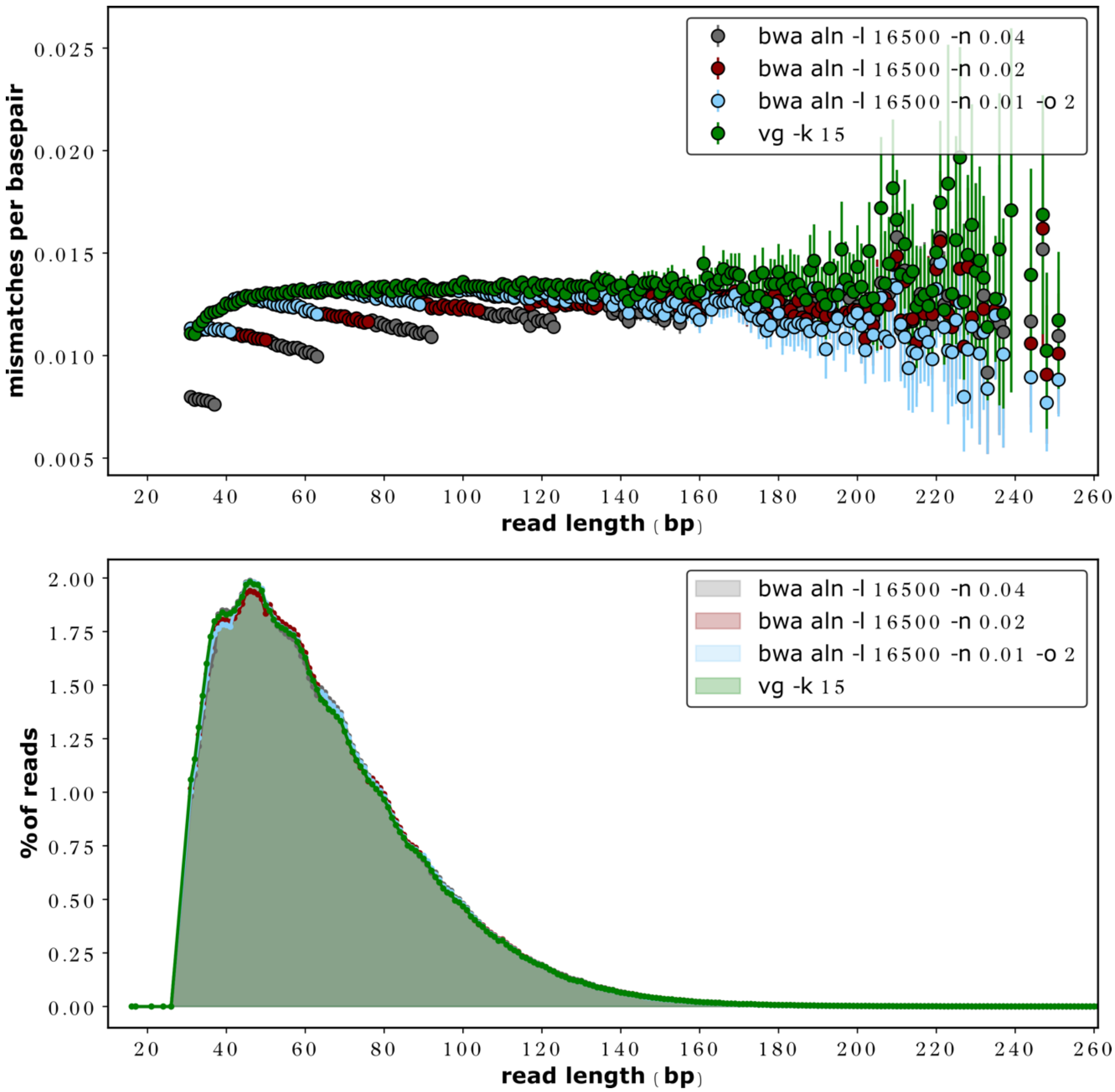

Figure S3. Mismatches per base pair by fragment size under various alignment settings. (Upper) Mismatch rates per base pair for different fragment sizes when aligned with different tools (vg vs. bwa aln) and settings in the case of bwa aln. Only reads with mapping quality >= 30 are included. (Lower) Distribution of mapped read lengths, reads below 30bp have been discarded. Figures created using AMBER (Dolenz et al. 2024).

Historical samples aligned to linear reference with bwa aln

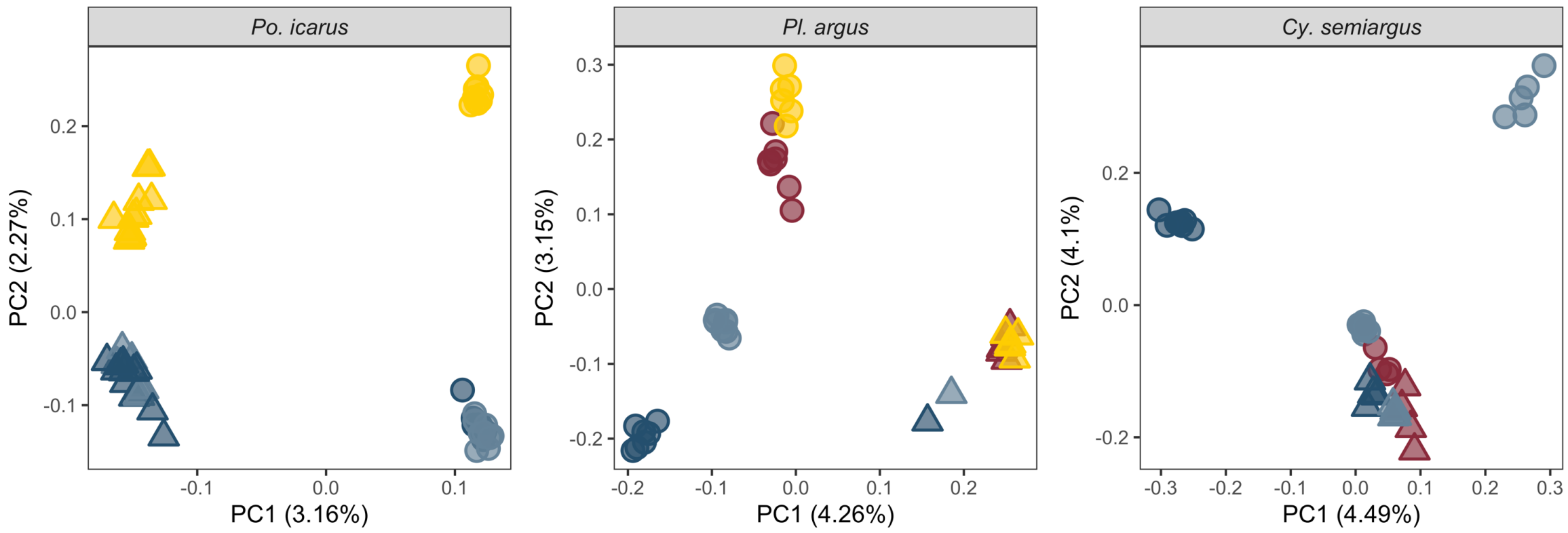

Historical samples aligned to variation graph with vg (subset from Figure 2)

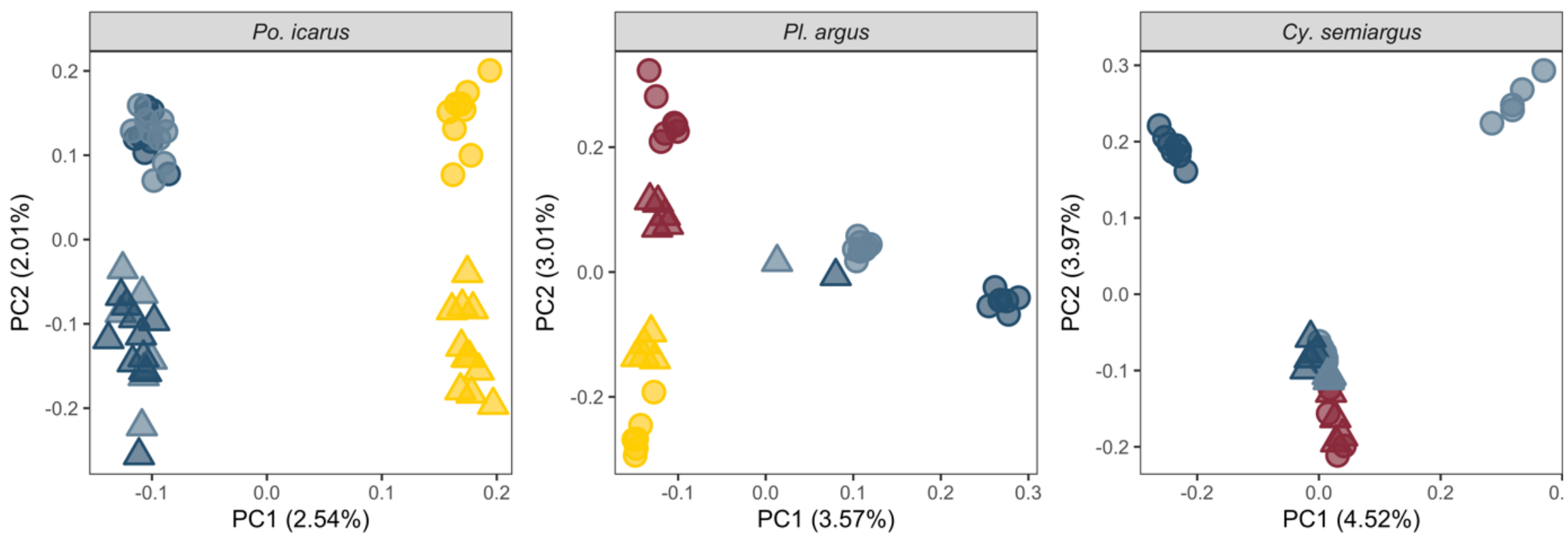

Figure S4. Comparison of downstream population structure analyses under different alignment methods. Principal component analyses (PCA) for the three focal species when historical samples are aligned to the linear reference genome with bwa aln (upper) and to the variation graph reference incorporating modern sample variants with vg (lower). Each circle represents a modern and each triangle a historical individual, with colors corresponding to the sampling locations in Figure 1B. Percentages on each axis refer to the proportion of total variance explained by the component. When aligned to a linear reference using bwa aln with relaxed parameters that reduce reference bias (-l 16500 -n 0.01 -o 2; Figure S3), we find that bias is still substantial enough that samples cluster primarily by sample type, as historical allele frequencies are artificially shifted towards the reference allele (upper panel; historical and modern samples separate on PC1 for *Po. icarus* and *Pl. argus* and on PC2 to some extent in *Cy. semiargus*). Utilizing a variation graph that incorporates common variants from the modern samples results in samples clustering by sampling locality (lower panel), as is expected, enabling more accurate comparison of the two different sample types. Clustering by sample type is still visible on PC2 for *Po. icarus* and may reflect the residual reference bias being greater in this species (Figure S2).

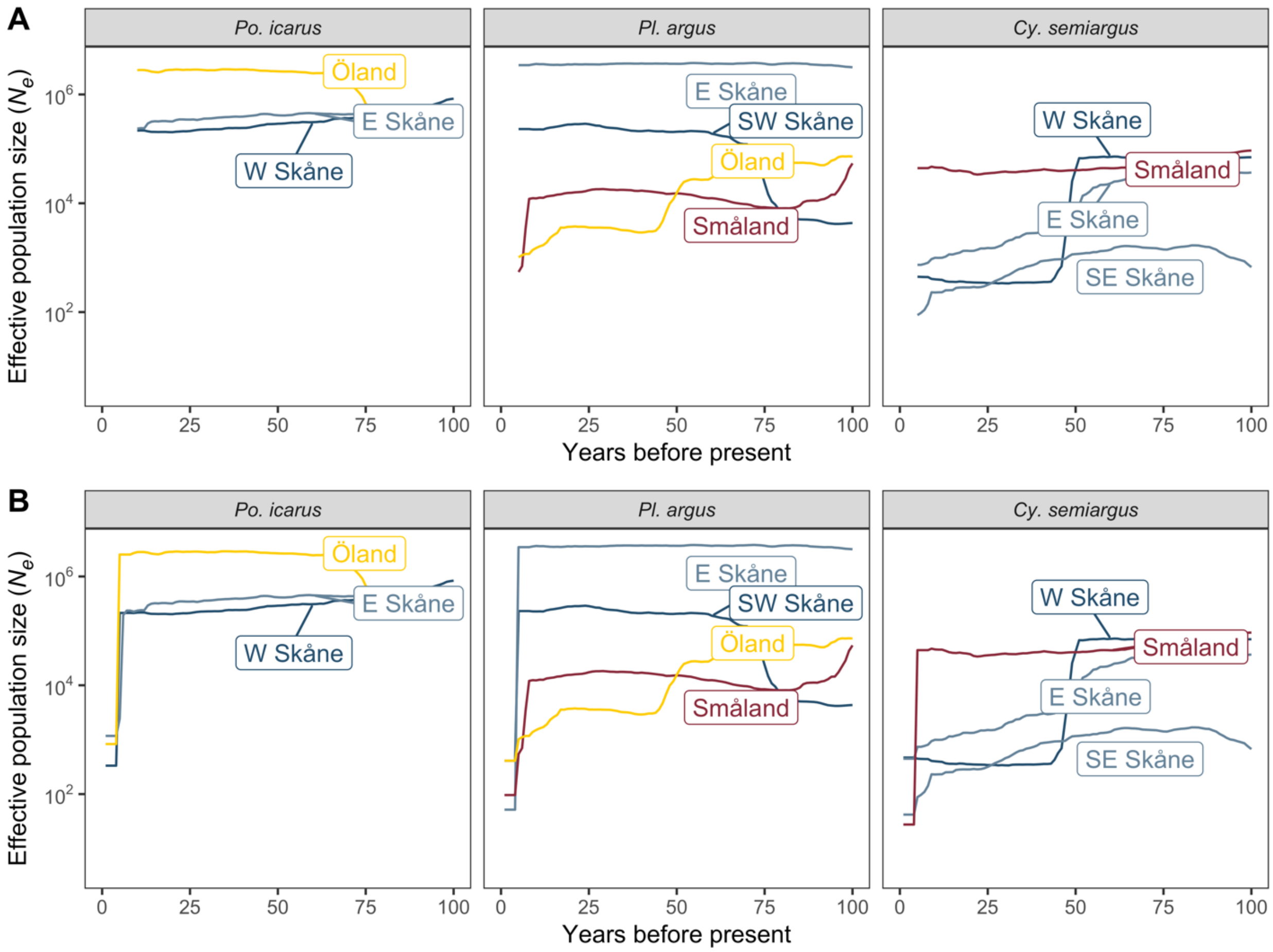

Figure S5. Effective population size trajectories for the past century estimated with GONE (Santiago et al. 2020). For each sampled modern population, we inferred effective population size changes over the past century using the linkage disequilibrium-based method implemented in GONE. Trajectories are presented here going backward in time and are presented on a log scale. Lines are colored and labeled by population for each species. As the most recent four generations often had a sharp decrease in effective population size, we present the trajectories here excluding (A) and including (B) these generations. As the change is almost always in the downward direction, we utilized the estimates at generation five for estimates of contemporary effective population size, as this provides a more conservative estimate. For parameterizing the coalescent simulations, we utilized the trajectories with the most recent four generations fixed to the effective population size of generation five (i.e. as depicted in A). Notably, the trajectory for SE Skåne *Cy. semiargus* is generally at a lower size than for the other populations of this species in Skåne. This is even more notable for sizes estimated for > 130 years before present (not depicted here), for which SE Skåne *Cy. semiargus* have an estimated effective size consistently below 10. Such exceptionally small historic sizes compared to the large ones (>> 10000) in other populations in Skåne may be a technical artifact resulting from the large bottleneck SE Skåne *Cy. semiargus* have experienced. Dramatic recent changes in N_e may erase signals of older demographic events (Santiago et al. 2020) potentially meaning the size estimates of older generations in the inferred trajectory for this population may be less accurate.

Po. icarus

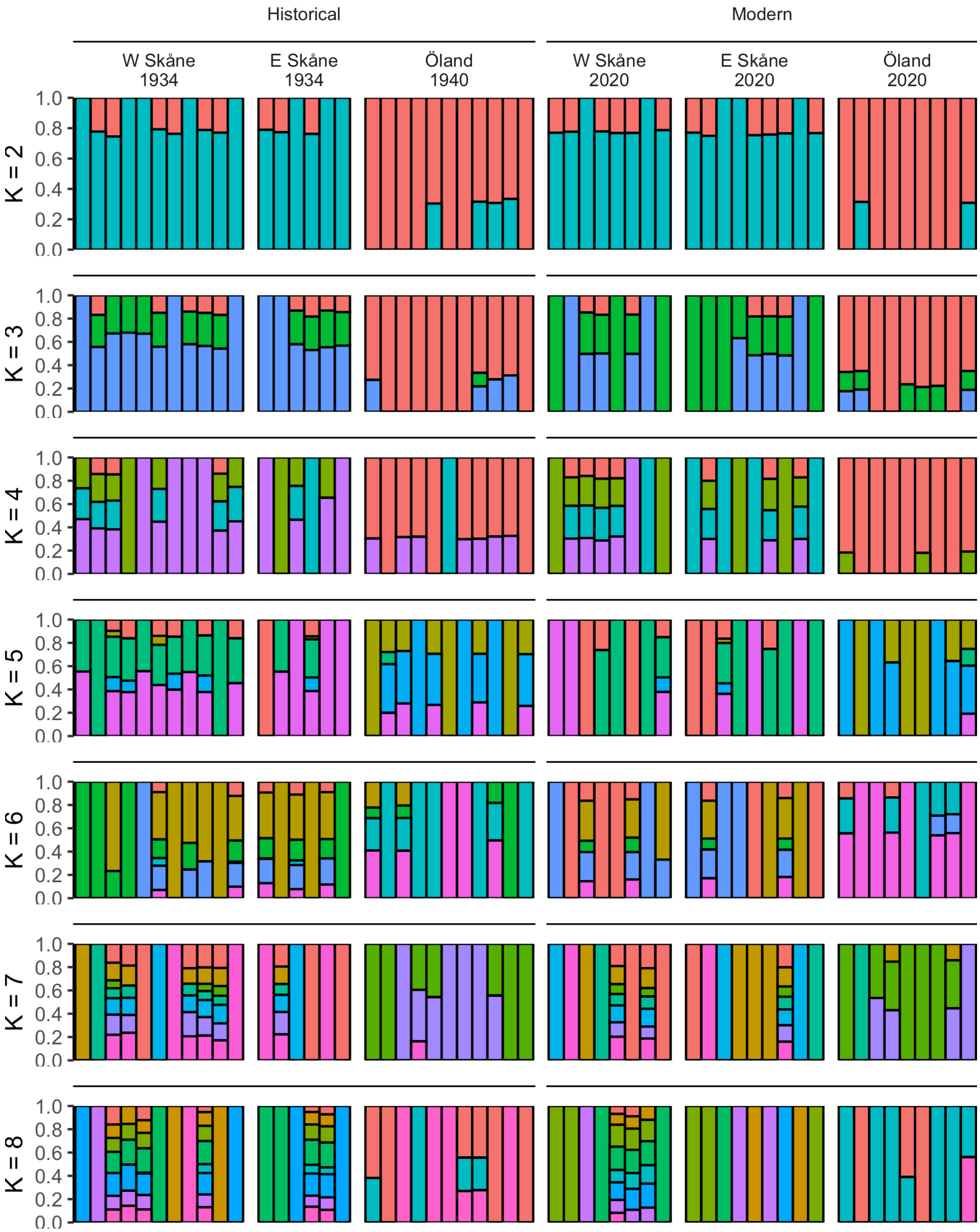

Historical
Modern
W Skåne 1934
E Skåne 1934
Öland 1940
W Skåne 2020
E Skåne 2020
Öland 2020
K = 2
K = 3
K = 4
K = 5
K = 6
K = 7
K = 8
1.0
0.8
0.6
0.4
0.2
0.0

Pl. argus

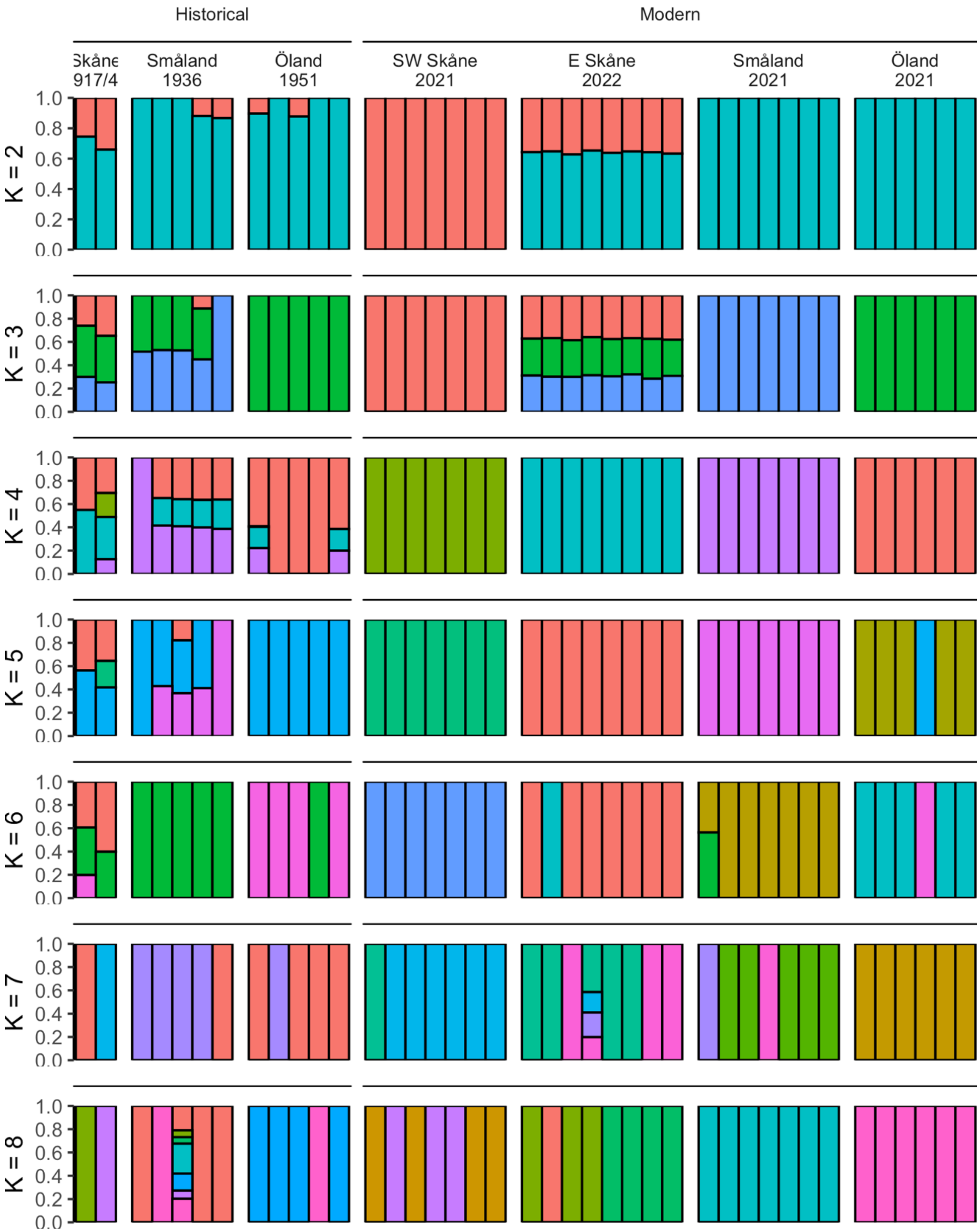

Historical
Modern
Skåne 1917/4
Småland 1936
Öland 1951
SW Skåne 2021
E Skåne 2022
Småland 2021
Öland 2021
K = 2
K = 3
K = 4
K = 5
K = 6
K = 7
K = 8
0.0
0.2
0.4
0.6
0.8
1.0

Cy. semiargus

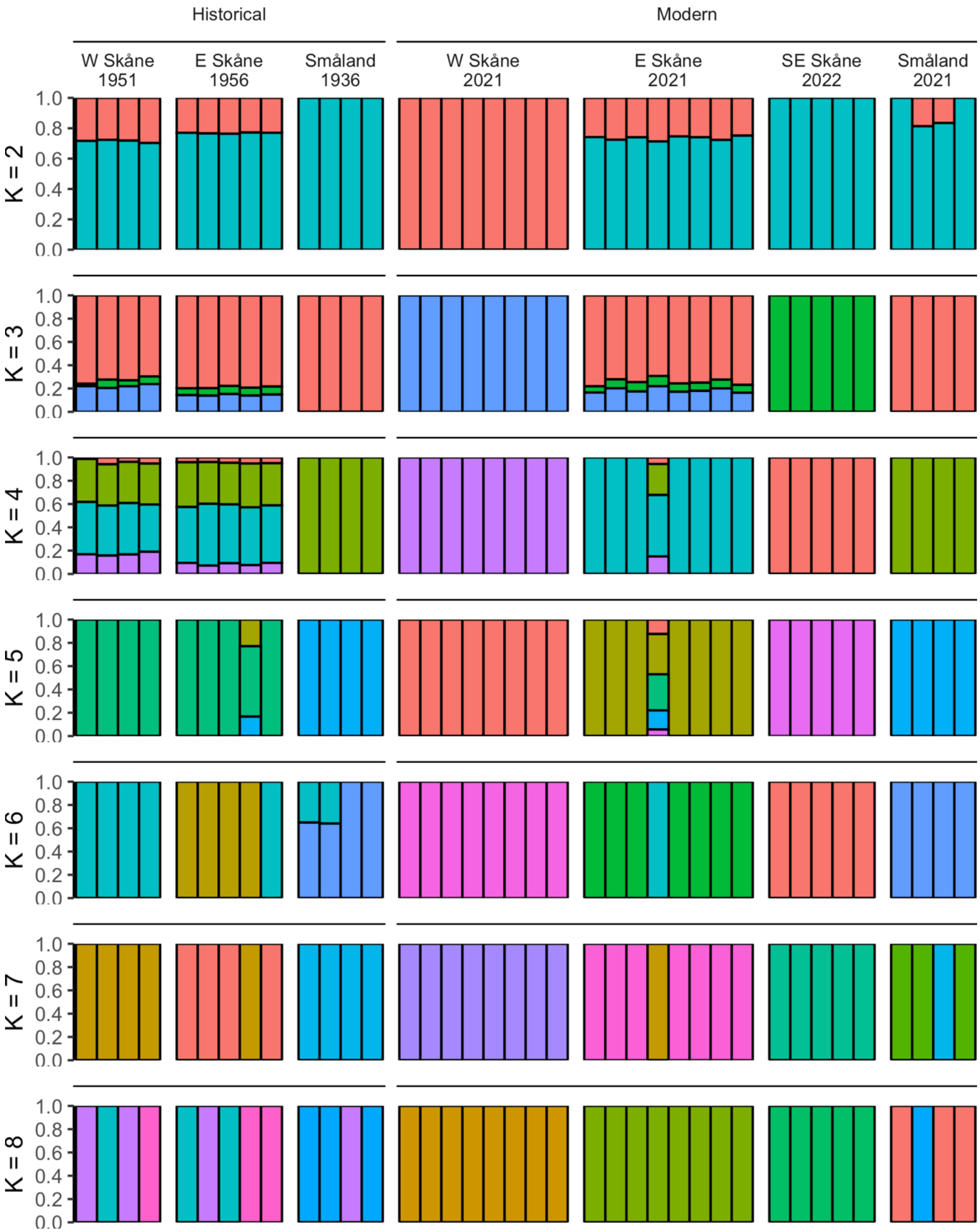

Figure S6. Admixture analysis results for K 2-8 for the three focal species. For each species, an admixture analysis was run for 2-8 clusters (K), with the optimized results for each value of K displayed in the figure above. We selected the best fitting K as the highest value of K that converged within 100 replicate runs of NGSadmix, following Pečnerová et al (Pečnerová et al. 2021). The best fit values of K are 1, 1, and 5 for *Po. icarus*, *Pl. argus*, and *Cy. semiargus*, respectively, with the best fit models depicted in Figure 2 in the main text for *Cy. semiargus* and K = 2 for *Po. icarus* and *Pl. argus*.

Po. icarus

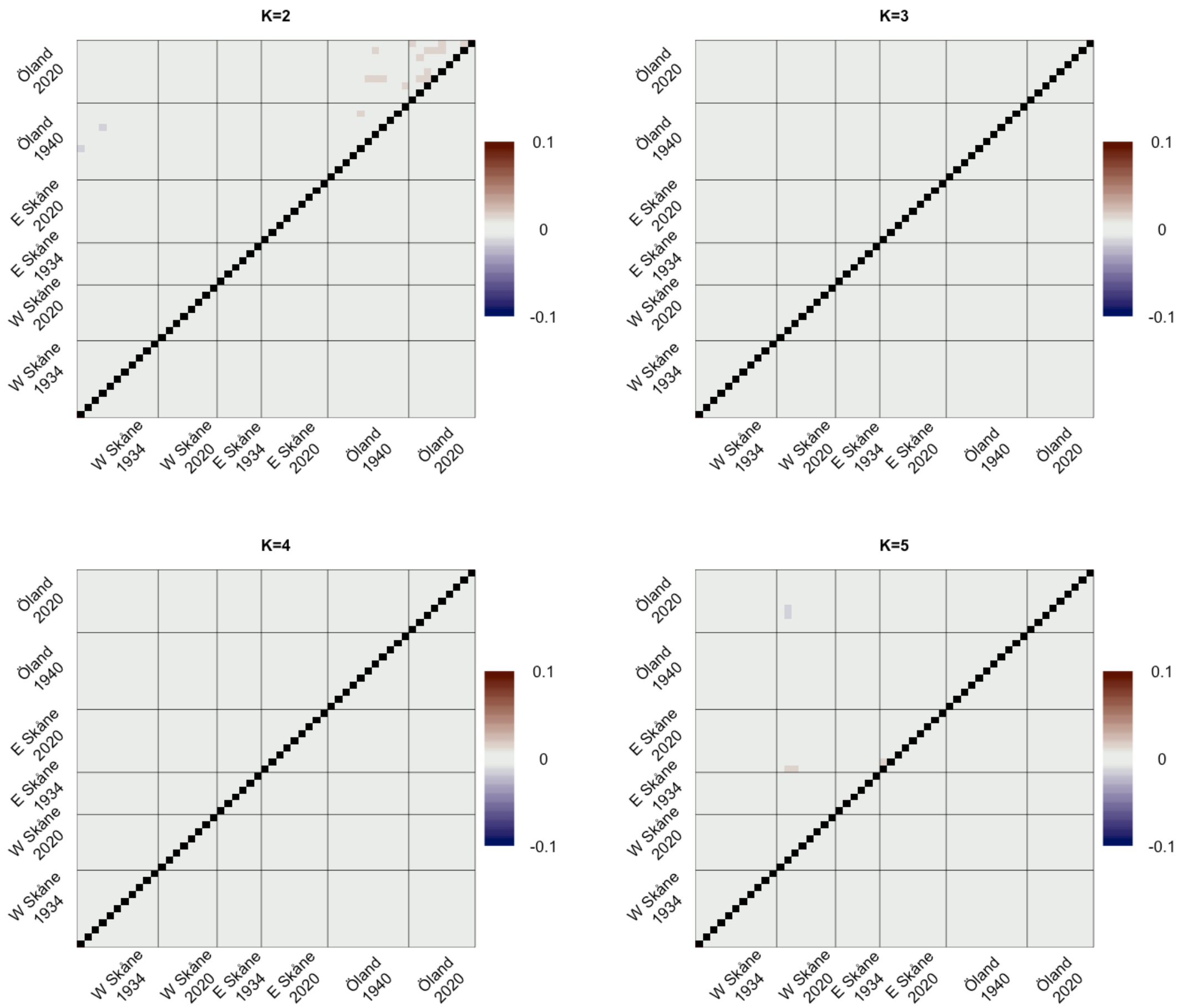
K=2
K=3
K=4
K=5
Öland 2020
Öland 1940
E Skåne 2020
E Skåne 1934
W Skåne 2020
W Skåne 1934
0.1
0
-0.1

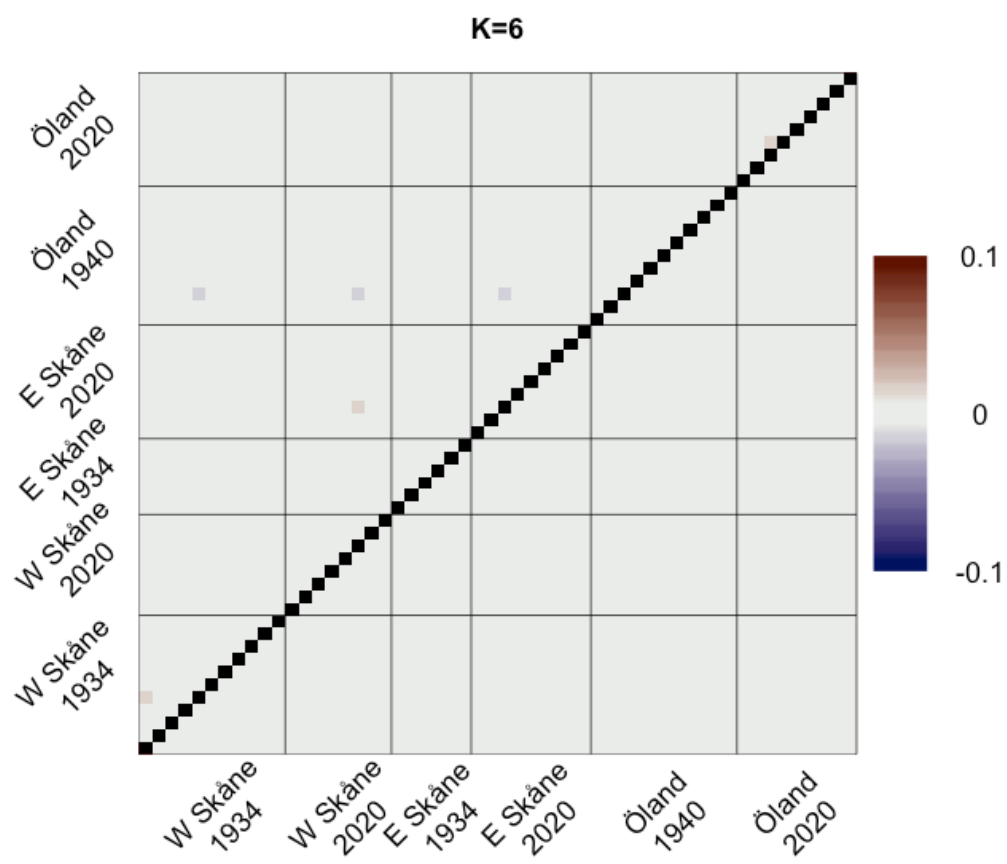
K=6
Öland 2020
Öland 1940
E Skåne 2020
E Skåne 1934
W Skåne 2020
W Skåne 1934
0.1
0
-0.1

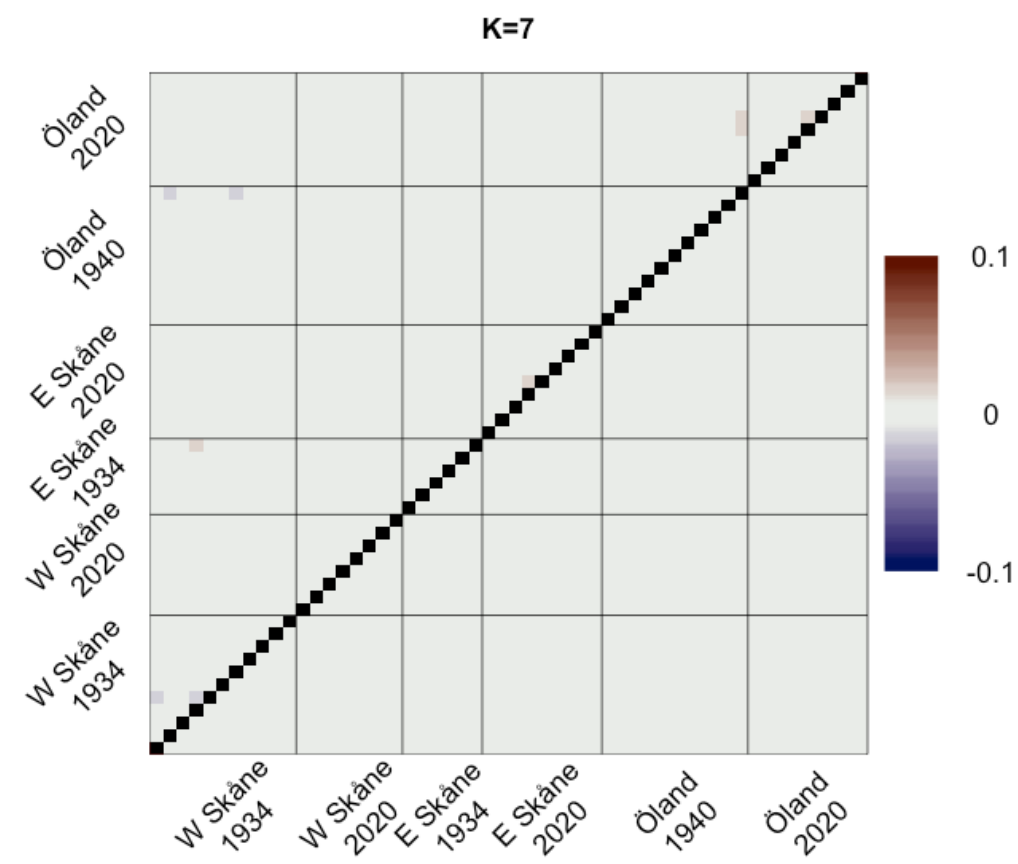
K=7
Öland 2020
Öland 1940
E Skåne 2020
E Skåne 1934
W Skåne 2020
W Skåne 1934
0.1
0
-0.1

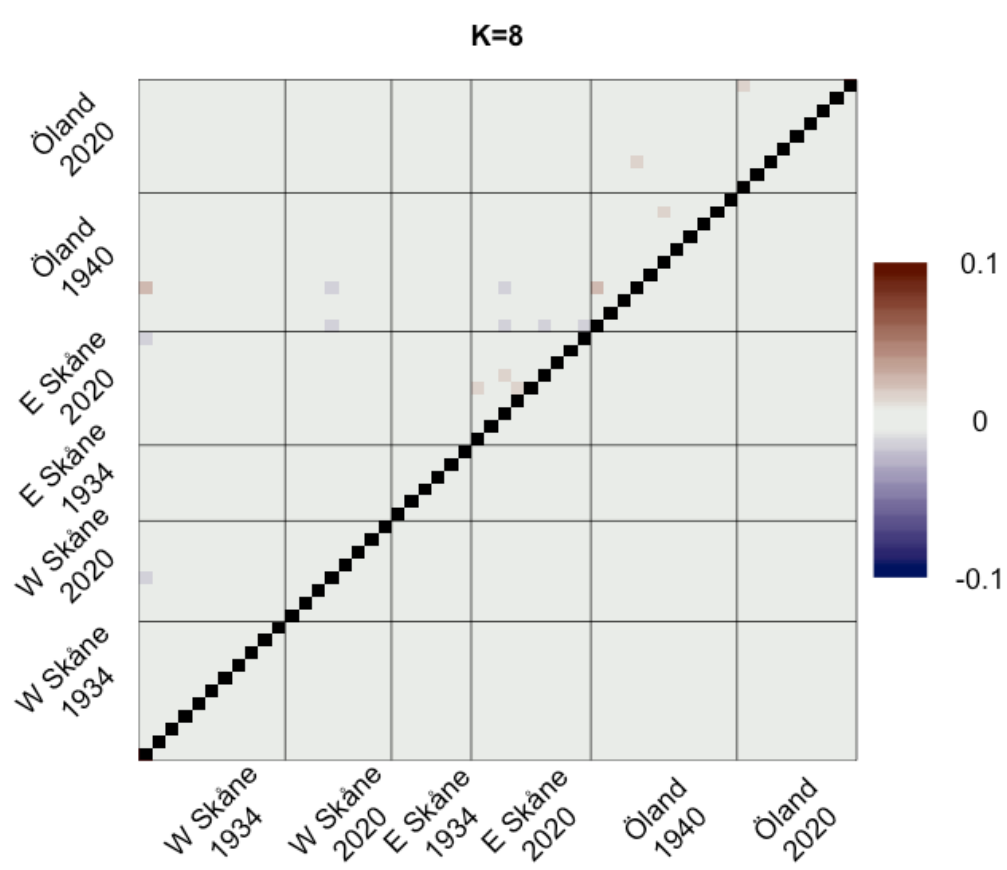
K=8
Öland 2020
Öland 1940
E Skåne 2020
E Skåne 1934
W Skåne 2020
W Skåne 1934
0.1
0
-0.1

Pl. argus

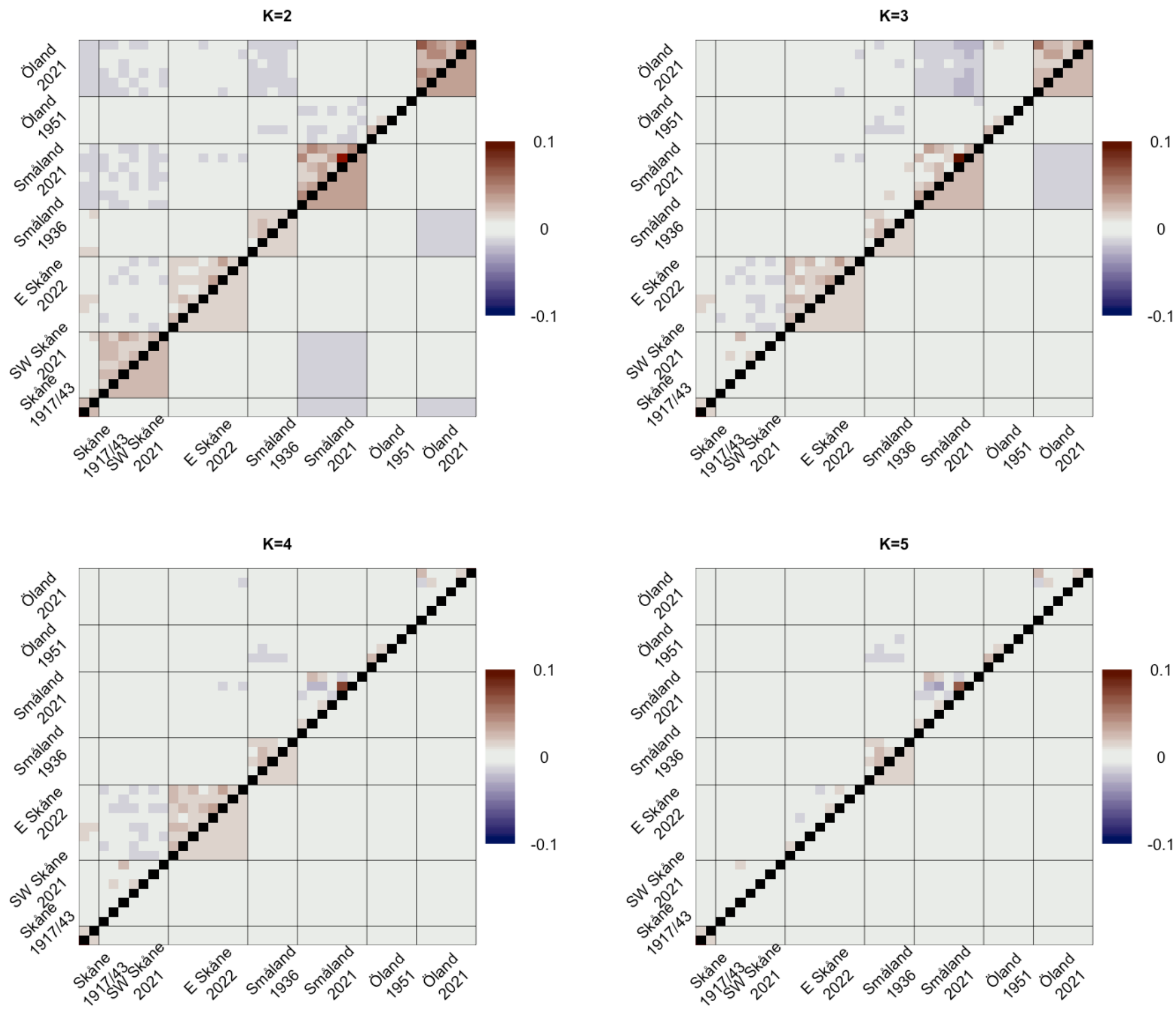
K=2
K=3
K=4
K=5
Öland 2021
Öland 1951
Småland 2021
Småland 1936
E Skåne 2022
SW Skåne 2021
Skåne 1917/43
0.1
0
-0.1

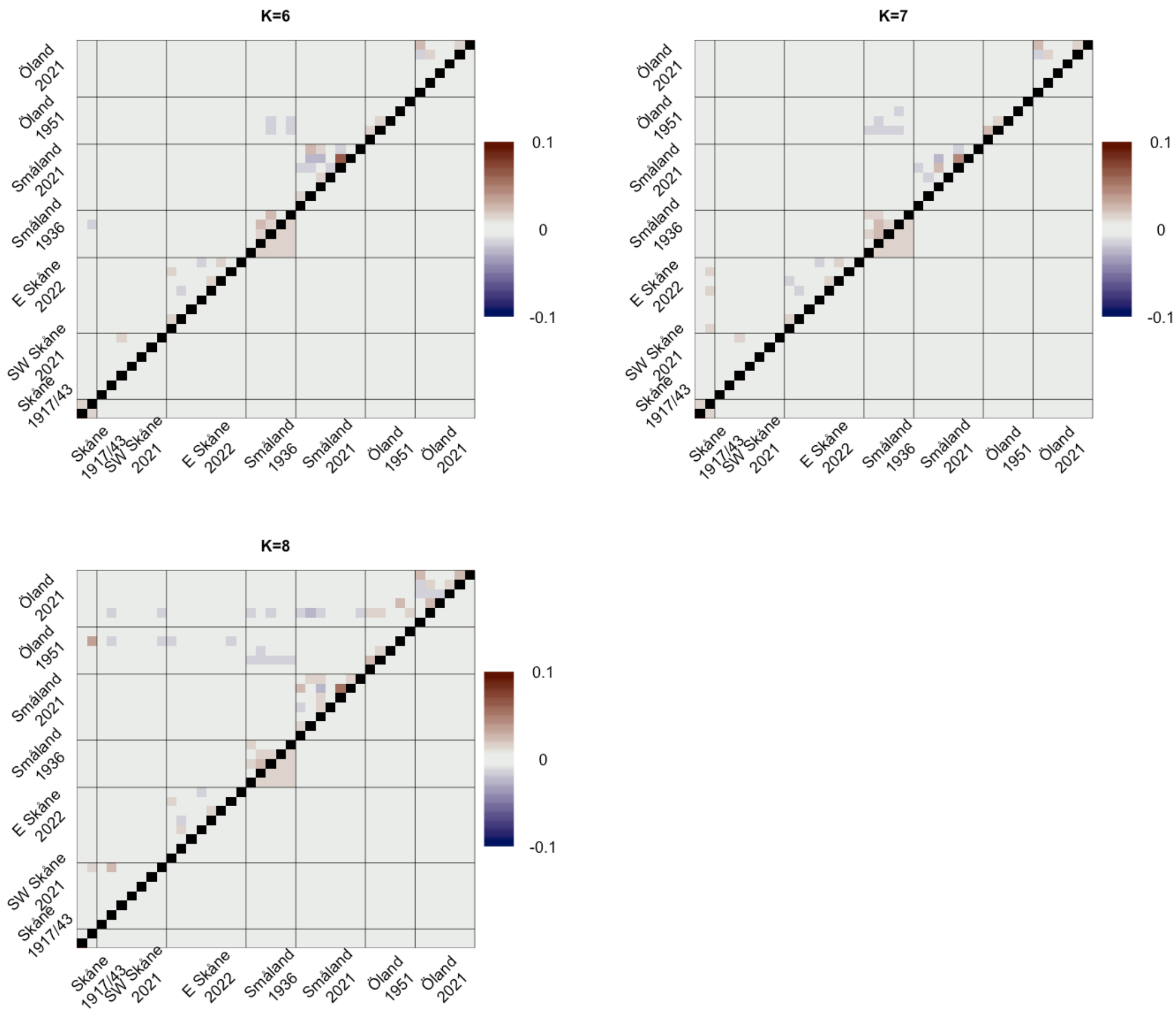
K=6
K=7
K=8
Öland 2021
Öland 1951
Småland 2021
Småland 1936
E Skåne 2022
SW Skåne 2021
Skåne 1917/43
0.1
0
-0.1

Cy. semiargus

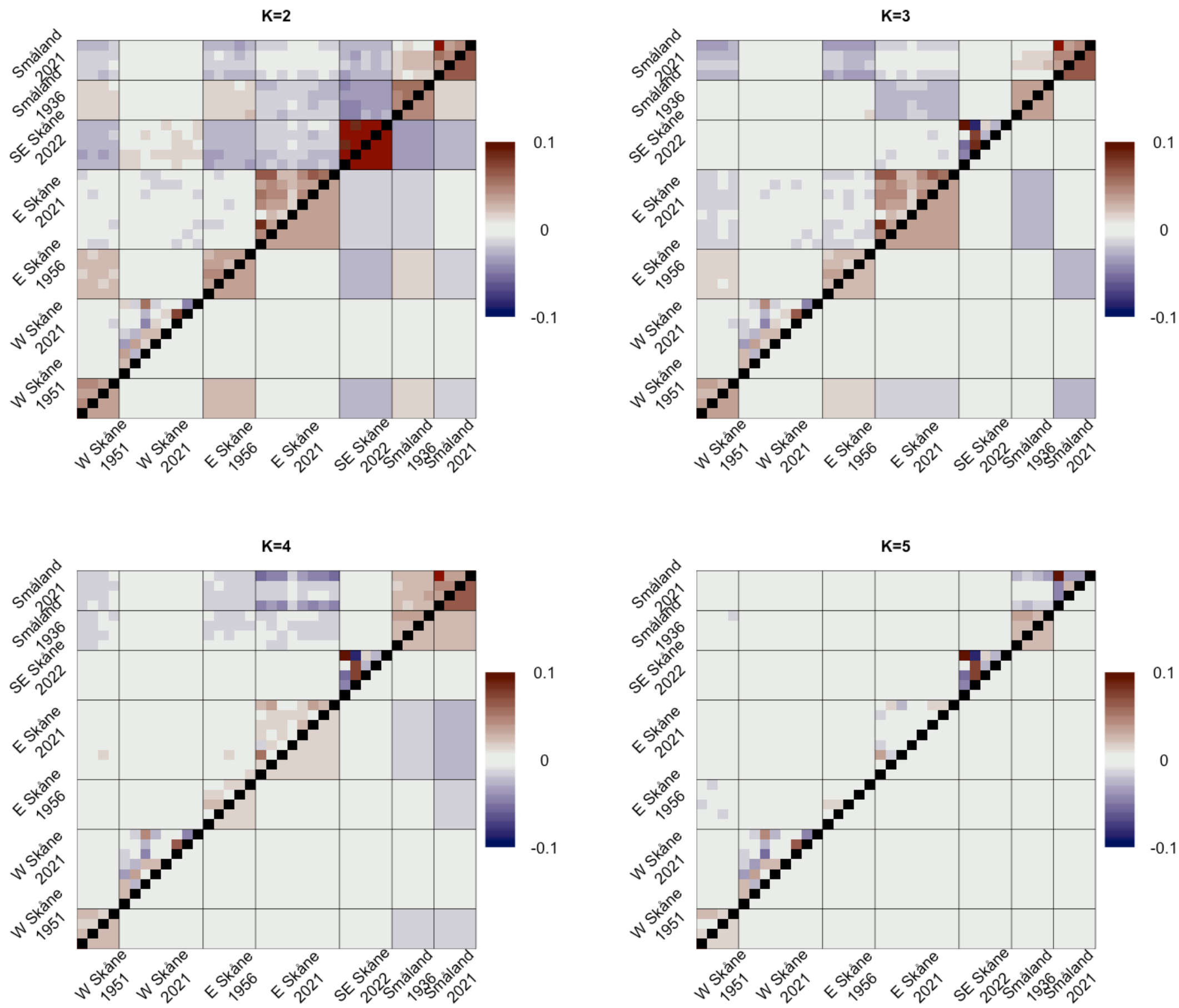

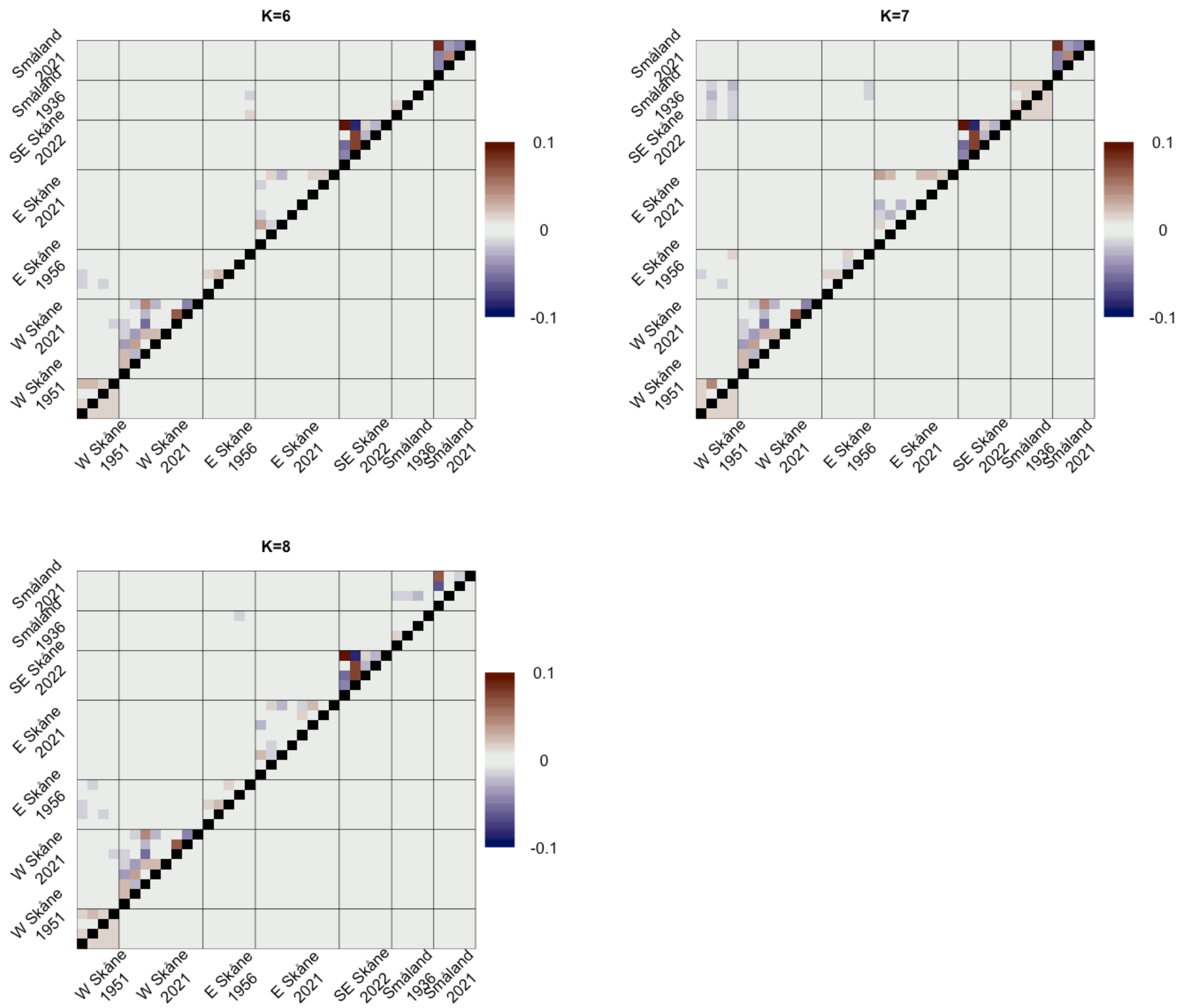

Figure S7. Correlation of residuals for the focal species' admixture analyses. For each value of K in the three focal species, we assessed the goodness of fit of our data to that K model of admixture using evalAdmix (Garcia-Erill and Albrechtsen 2020), visualized here with the pairwise correlation of residuals between individuals (upper left triangle of the matrix). When individuals are more similar than the model suggests, they have a positive pairwise correlation, when they are more different, they have a negative. Well-fitting sample pairs have a score of 0. The lower right triangle of the matrix shows the average scores of individuals in our *a priori* population groupings, illustrating when it is largely a population's fit that is in need of improvement. While we select K = 1 as our best fit based on our convergence criteria for *Po. icarus* and *Pl. argus*, we see that the data does not necessarily fit the model well in *Pl. argus*, illustrating that there is some genetic structure, as supported by the PCA in Figure 2. Our inability to achieve convergence for K-values above 2 in these species may relate to additional clusters being explained by a relatively small proportion of the genetic variance, requiring higher sample sizes to accurately assign admixture proportions at higher Ks. We do select a higher K, K = 5, for *Cy. semiargus*, which shows relatively good fit.

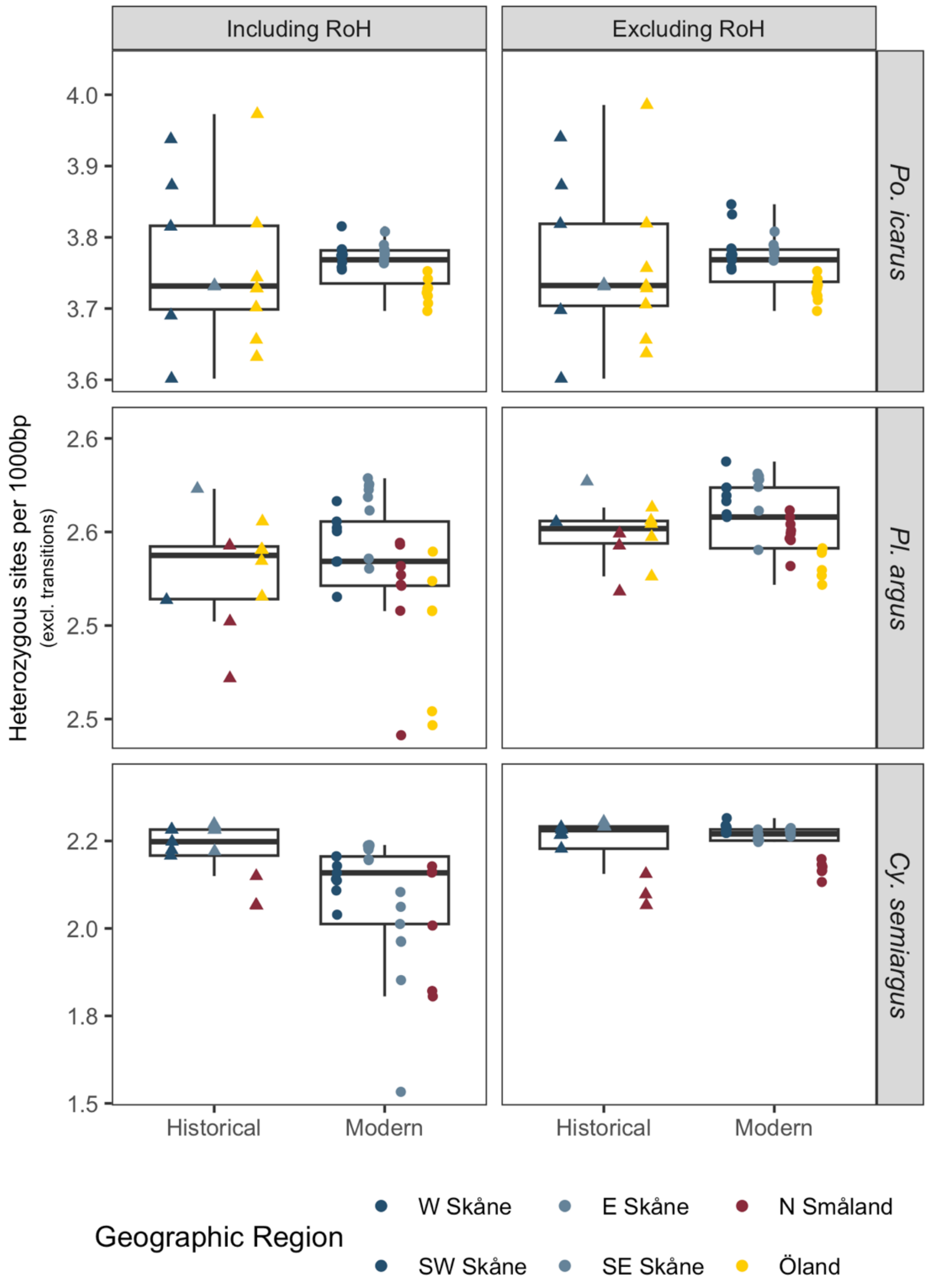

Figure S8. Heterozygosity estimates including and excluding runs of homozygosity. To assess the extent by which runs of homozygosity contributed to lower estimates of heterozygosity in modern samples, we calculated heterozygosity adjusted by F_{RoH} as: Heterozygosity / (1 - F_{RoH}). This adjusted estimate provides an approximation of the heterozygosity expected outside of, or excluding *RoH*. This figure depicts the whole genome estimates of heterozygosity on the left (including *RoH*) and the estimates adjusted by F_{RoH} on the right (excluding *RoH*). Points represent individuals, colored by sampling population, with boxplots encompassing all individuals for a given time period, as in Figure 1. Much of the difference in heterozygosity in *Cy. semiargus* visible from the whole genome estimates on the left is accounted for by runs of homozygosity, as the differences, even to the population level, are absent once heterozygosity is adjusted for *RoH* on the right.

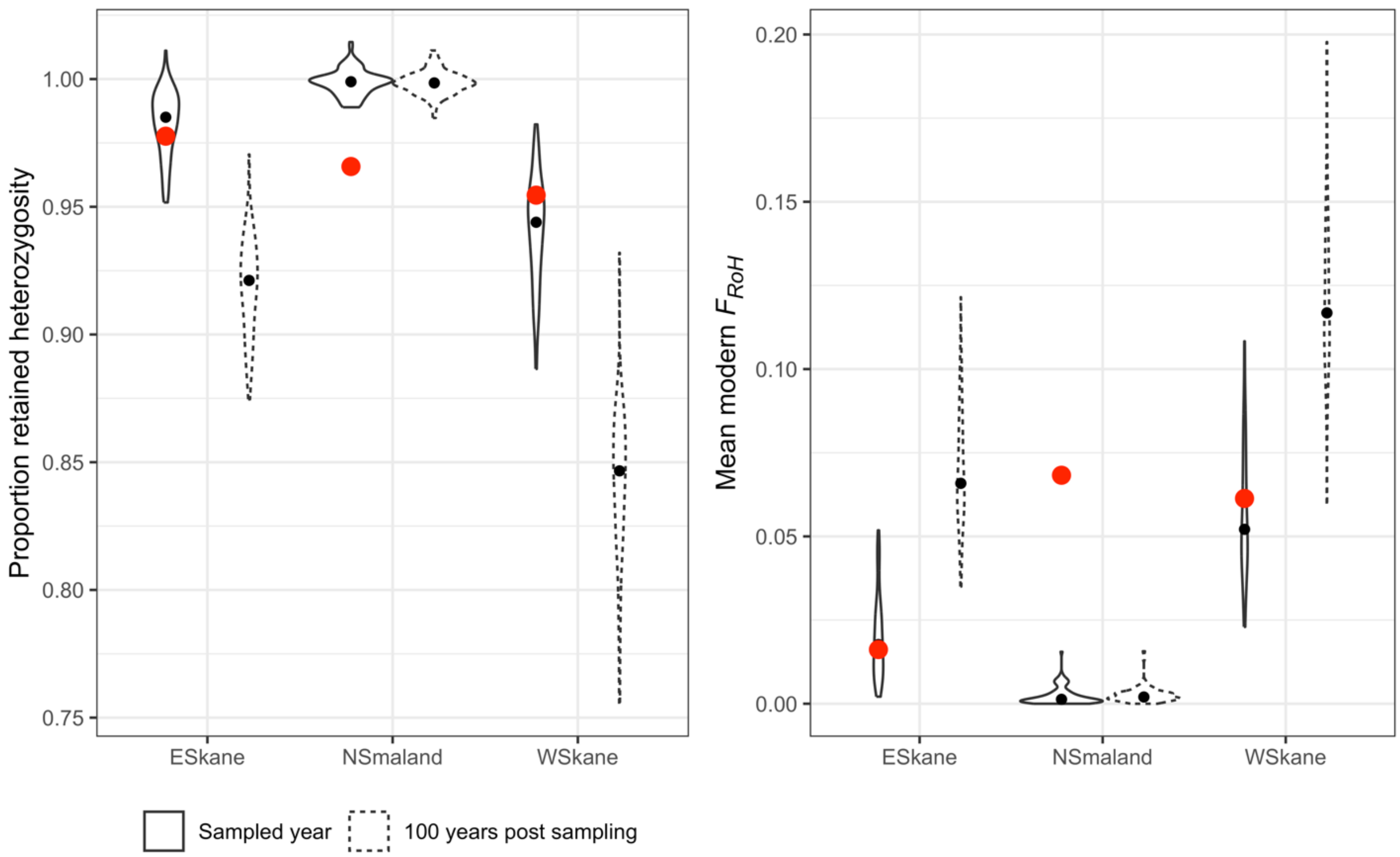

Figure S9. Simulated expectations for retained heterozygosity and modern F_{RoH} in *Cy. semiargus*. To assess if the patterns of reduction in heterozygosity and increase in inbreeding coefficients in *Cy. semiargus* we estimated empirically from historical and modern genomes were reasonable under population genetic expectations, we performed coalescent simulations that included the period between historical and modern sample collection as well as a period 100 years after modern sample collection. The period between historical and modern sampling is modeled using the effective population size trajectories inferred from the modern samples with GONE, with the 100 years after modern sampling set to maintain the most recent inferred effective population size. Depicted here are the distributions of heterozygosity retained (relative to the historical collection year, left) and mean F_{ROH} (right) at the modern collection time point (solid violins) and 100 years later (dashed violins) from 100 simulation replicates per population. Black points refer to the median prediction across simulations and the red point represents the empirical estimates of the metrics made from the comparison of historical and modern genomes. For E Skåne and W Skåne, empirical estimates fall within expected ranges for both heterozygosity and F_{RoH}. We also observe a continued reduction in heterozygosity and increase in F_{RoH} occurs in these populations over the 100 years after sampling, despite the population maintaining a stable size over the period, illustrating the lag in genetic diversity loss compared to demographic decline. In Småland *C. semiargus*, a stable demographic history was inferred with GONE (Figure S5) over which the simulations predict stable heterozygosity and F_{RoH}, while we estimate a reduction in heterozygosity from our data. Such a disparity could be caused by artifacts in empirical estimates of heterozygosity and F_{RoH} or the inferred demographic history from GONE. As our empirical estimates match patterns in the other populations, i.e. the reduction in heterozygosity between historical and modern samples is largely explained by the magnitude of increase in F_{RoH}, we suggest a comparable demographic decline has occurred in this population that was not able to be detected in the GONE analysis.

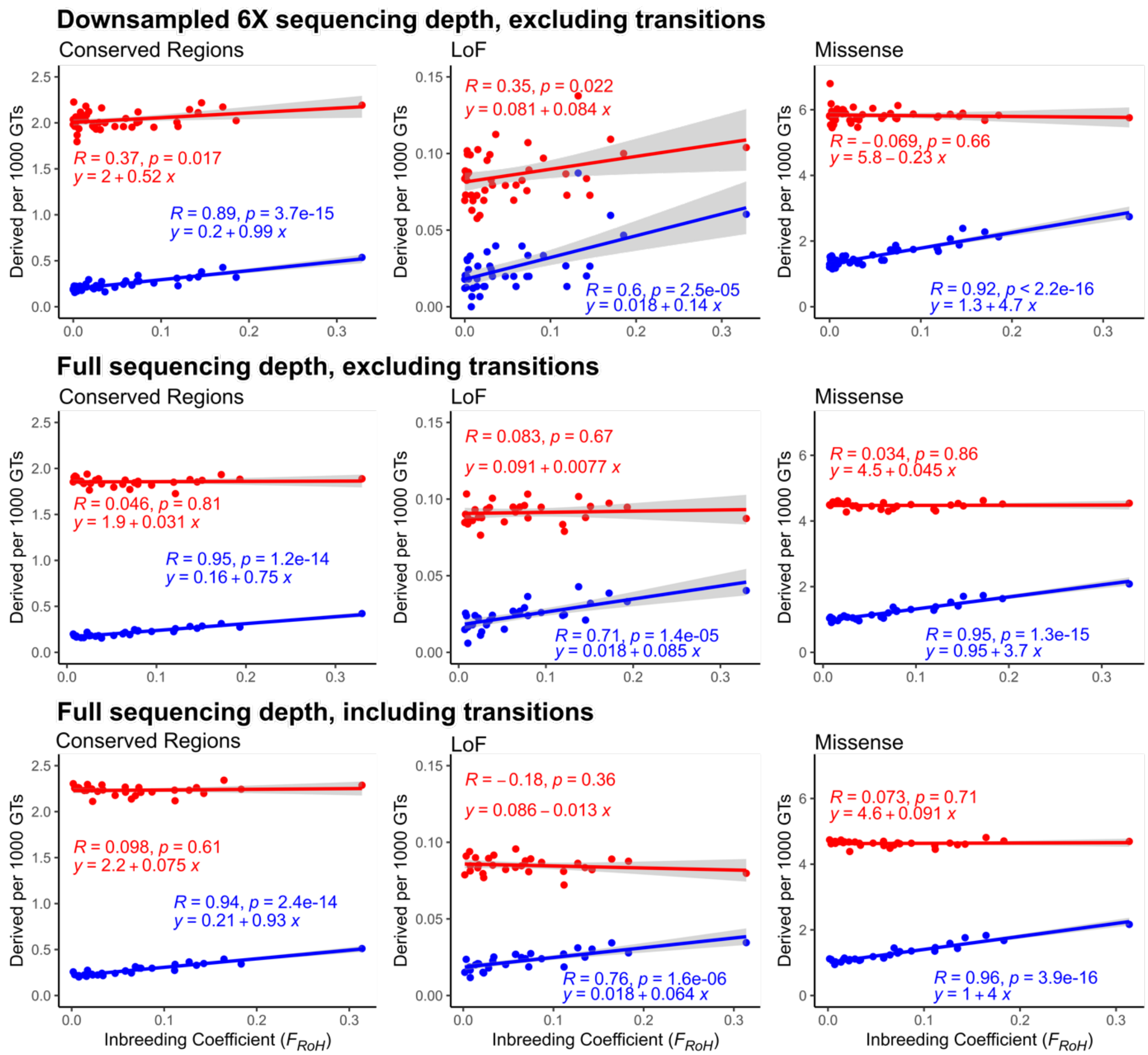

Figure S10. Linear models examining the relationship between individual inbreeding coefficient and deleterious burden in modern *Cy. semiargus* across putatively deleterious mutation categories. Red points and lines depict total estimates of deleterious burden, measured as counts of derived alleles for the given putatively deleterious category per 1000 genotyped positions. Blue points and lines depict homozygous deleterious burden, measured in the same way, but only counting homozygous derived alleles in the numerator. For each linear model, the *R*- and *p*-value along with the equation of the regression line are depicted on the plot in the same color. The analysis was performed with variants filtered with three approaches: individuals downsampled to 6x depth with transitions removed (top, methodology of main manuscript), at full depth excluding transition variants (middle), and at full depth, including transition variants (lower). The three filter sets estimate similar derived counts across all mutation categories, as well as similar relationships between derived counts and inbreeding coefficients. Total deleterious burden has a weak, but significant relationship with inbreeding coefficients for conserved and loss of function variants in the downsampled 6x data, but this relationship is not present for either full depth set. Homozygous deleterious burden is positively related to inbreeding coefficient across all variant categories and filtering sets, with the relationship between missense and conserved variants with inbreeding coefficients being stronger than that of loss of function variants. Inclusion or exclusion of transition variants had little impact on the estimated derived count at missense positions, despite transversions being more often non-synonymous. This is due to the counts being scaled by the genotyping rate and by a transition biases in this species (exon ts/tv = 2.24; intergenic ts/tv = 1.08) resulting in close to even abundance of transitions and transversions at missense positions (missense ts/tv = 1.17).

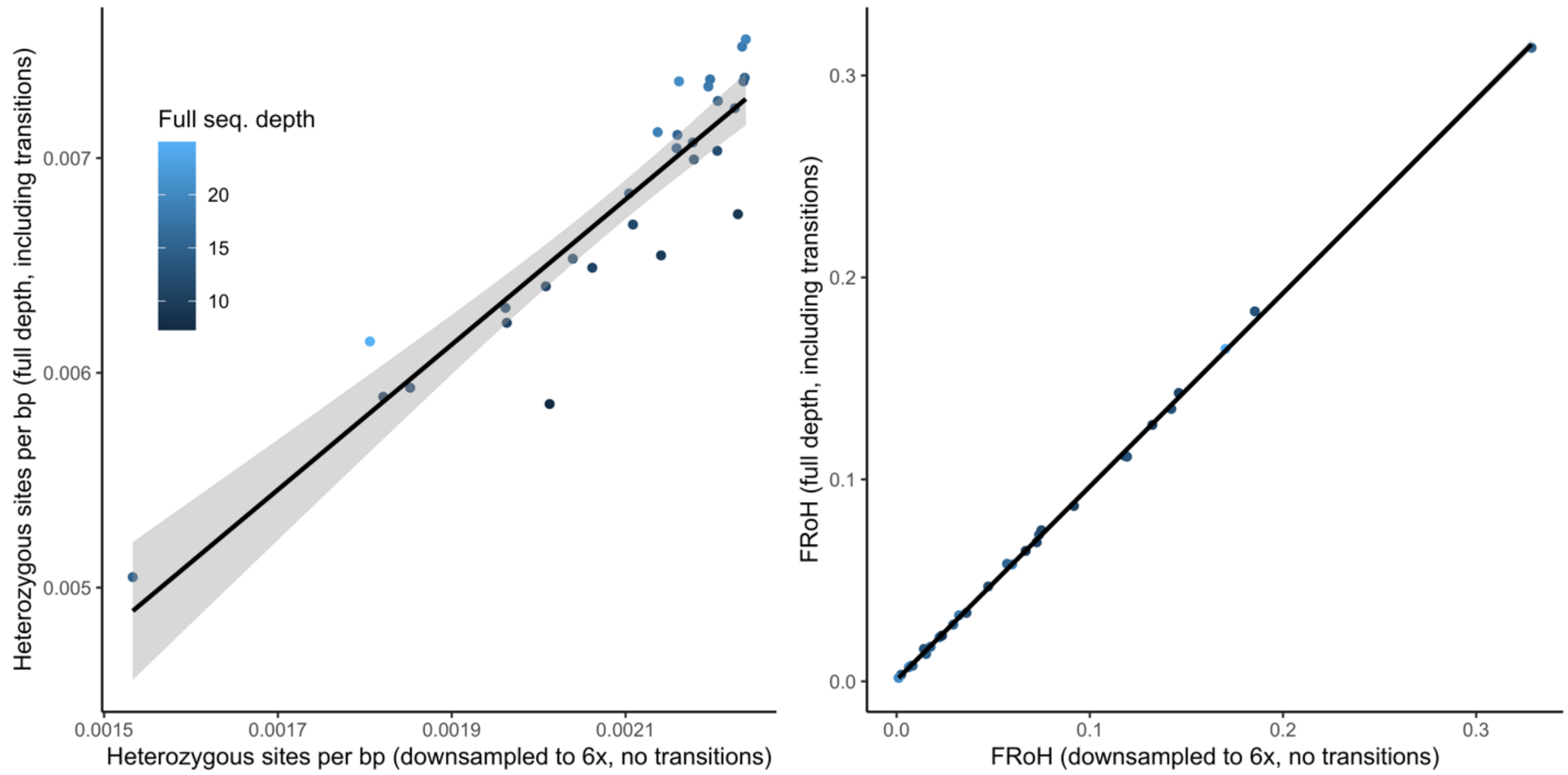

Figure S11. Comparison of estimates of individual heterozygosity (left) and F_{RoH} (right) for modern *Cy. semiargus* samples subsampled to 6x depth with transitions removed (x-axis) and at full depth, including transitions (y-axis). The full depth of the sample is indicated by the color of the point. Lines in each plot represent a linear model with the focal metric at full depth as a response to the metric at subsampled depth, with the standard error depicted as a grey area surrounding the line. X- and y-axis scales for heterozygosity differ, as the exclusion of transitions and reduced power to call heterozygous sites at low depth reduce the absolute value estimated. However, for both heterozygosity and F_{RoH}, the relationship between individual estimates of heterozygosity and F_{RoH} are strongly related, indicating that the estimates at 6x depth without transitions are suitable for comparisons between individuals as performed in this study. Notably, the model-based inference of F_{RoH} appears highly robust to variation in sequencing depth and inclusion/exclusion of transition variants.

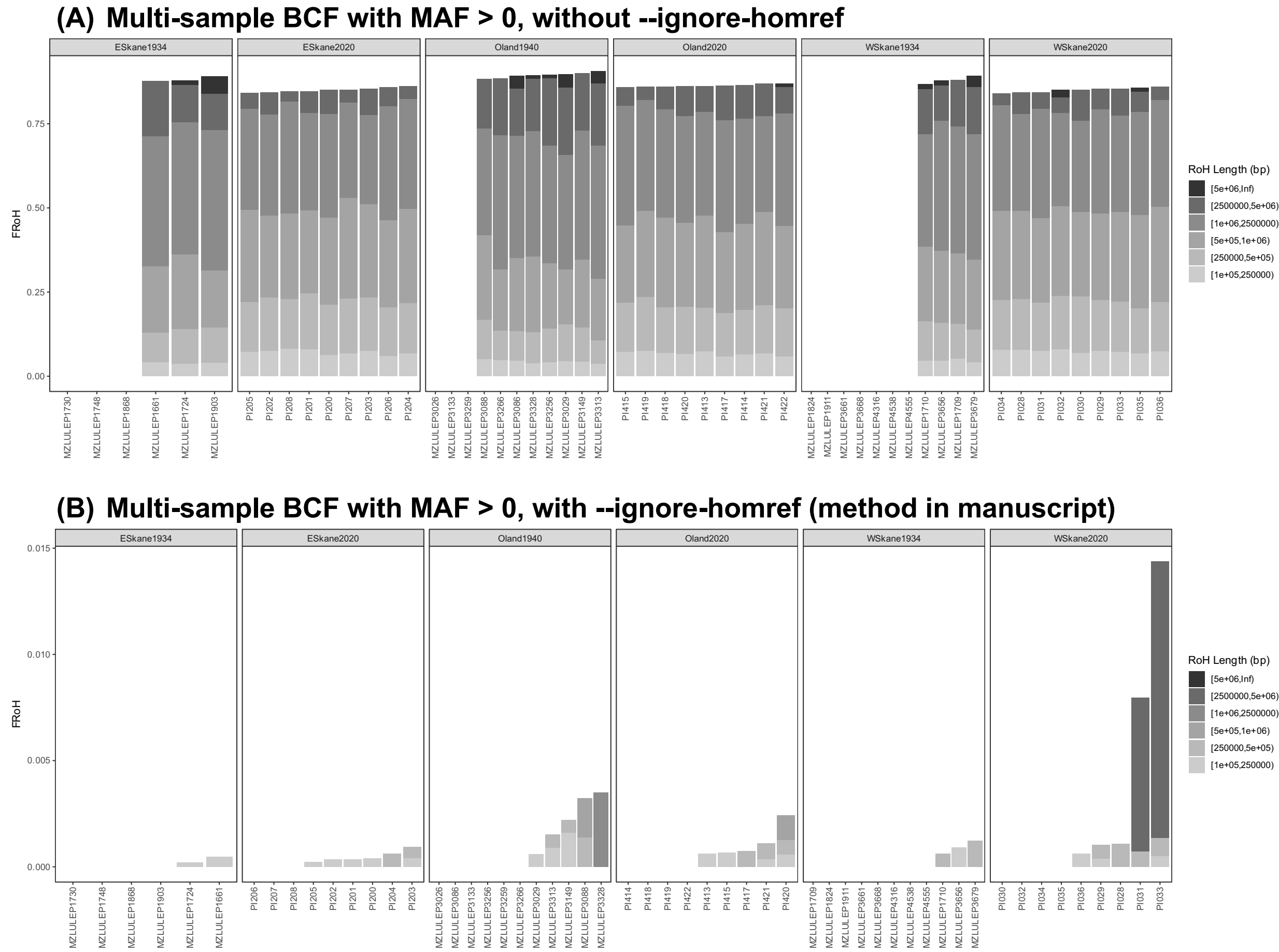

Figure S12. Impacts of including homozygous reference genotypes when estimating runs of homozygosity. BCFtools/RoH utilizes deviations from Hardy-Weinberg to detect runs of homozygosity, requiring an allele frequency for each variant to estimate this. In datasets where allele frequencies are unknown, a default can be used, commonly a minor allele frequency of 0.4. However, singleton and other low frequency variants deviate considerably from this default, and can heavily inflate the estimated runs of homozygosity, as in (A) where nearly all individuals of *Po. icarus* in our dataset are described as having almost the entire genome in runs of homozygosity. Two potential mechanisms to address this would be to estimate allele frequencies for the populations and supply them (as we previously did for the modern samples of *Po. icarus* in (Nolen et al. 2024)), or filter out low frequency variants with a minor allele frequency filter. We have considerable variation in sample sizes across our species datasets, making a consistent MAF filter or comparable allele frequency estimates challenging, so we did not use either of these approaches. Instead, we utilized the option to ignore homozygous reference variants, with the results shown in (B). This reduces the effect, as the inflation largely comes from homozygous reference variants present in the BCF due to rare, alternate variants present in other individuals. When performed in this way, the analysis is similar to that done for variants called from a single sample the single sample as described in BCFtools online guide (https://samtools.github.io/bcftools/howtos/roh-calling.html). Using this approach, the estimates we get for all individuals show less than 1.5% of the genome in runs of homozygosity for all individuals, in line with the estimates we generated using population specific allele frequencies in (2024). One main difference to performing the analysis on a single sample is that by using a multi-sample BCF, we filter out alternate variants that are fixed in the study individuals, as the reference allele is unlikely segregating, or segregating in low frequency, in the study populations, and would deviate from our relatively high default allele frequency.

Table S1. Sample collection and sequencing information. Each sample utilized in this study is listed here, providing its sample ID as referenced in the manuscript text/figures/code. Most specimens were registered into the entomological collections of the Biological Museum at Lund University, and has its museum identifier provided (MZLU ID). Each specimen is assigned a Time Period, which corresponds to whether it is being treated in the Historical or Modern sample grouping. As some samples in the Modern category were pinned specimens, the origin of the tissue used for DNA extraction is provided (Tissue Source). Study Region refers to the population grouping for population level analyses, grouping samples in the same study region and time period together. Sampling Locality refers to the name of the locality in which the sample was collected, either using the authors' designation for a sampling locality for fresh specimens or using the locality listed on the label for pinned specimens. For both, latitude and longitude of the locality is provided. For fresh specimens, these values correspond to the specific location at which butterflies were sampled, for pinned specimens these coordinates are inferred from the locality listed on the label, which is usually the nearest parish from the actual sampled grassland patch. For both fresh and pinned samples, the collection date and collector are provided, with collection dates that could not be fully inferred from the label having missing information replaced with 'xx'. For each sample, we report the total percent of sequencing reads mapping to the reference genome (% Mapped Reads) both when mapped with bwa mem/aln and vg, as well as the mean sequencing depth after filtering for each aligner.

Sample ID	MZLU ID	Species	Time Period	Tissue Source	Sex	Collect Date	Study Region	Sampling Locality	Latitude	Longitude	Collector	% Mapped Reads (bwa)	% Mapped Reads (vg)	Filtered Depth (bwa aln/mem)	Filtered Depth (vg)
MZLULEP1724	MZLULEP1724	*Polyommatus icarus*	Historical	Pinned Specimen (abdomen)	M	1934-06-03	E Skåne	Kivik	55.685	14.2266	P. Benander	90.25	100	5.3542	5.8738
MZLULEP1868	MZLULEP1868	*Polyommatus icarus*	Historical	Pinned Specimen (abdomen)	M	1934-06-03	E Skåne	Kivik	55.685	14.2266	P. Benander	89.81	100	2.0647	2.2375
MZLULEP1730	MZLULEP1730	*Polyommatus icarus*	Historical	Pinned Specimen (abdomen)	M	1934-06-06	E Skåne	Kivik	55.685	14.2266	P. Benander	90.26	100	3.2782	3.5842
MZLULEP1748	MZLULEP1748	*Polyommatus icarus*	Historical	Pinned Specimen (abdomen)	M	1934-06-06	E Skåne	Kivik	55.685	14.2266	P. Benander	89.23	100	3.2075	3.5499
MZLULEP1661	MZLULEP1661	*Polyommatus icarus*	Historical	Pinned Specimen (abdomen)	M	1934-07-25	E Skåne	Kivik	55.685	14.2266	P. Benander	87.85	100	6.0072	6.6505
MZLULEP1903	MZLULEP1903	*Polyommatus icarus*	Historical	Pinned Specimen (abdomen)	M	1934-07-25	E Skåne	Kivik	55.685	14.2266	P. Benander	89.81	100	5.6560	6.2317
MZLULEP3086	MZLULEP3086	*Polyommatus icarus*	Historical	Pinned Specimen (abdomen)	M	1940-07-28	Öland	Vickleby	56.5766	16.4604	Winblad, Gustaf	89	100	6.7512	7.5855
MZLULEP3149	MZLULEP3149	*Polyommatus icarus*	Historical	Pinned Specimen (abdomen)	M	1940-07-28	Öland	Vickleby	56.5766	16.4604	Winblad, Gustaf	89.19	100	10.6416	11.7809
MZLULEP3328	MZLULEP3328	*Polyommatus icarus*	Historical	Pinned Specimen (abdomen)	M	1940-07-28	Öland	Vickleby	56.5766	16.4604	Winblad, Gustaf	87.93	100	8.5818	9.6501
MZLULEP3026	MZLULEP3026	*Polyommatus icarus*	Historical	Pinned Specimen (abdomen)	M	1940-07-30	Öland	Vickleby	56.5766	16.4604	Winblad, Gustaf	88	100	3.9545	4.4624
MZLULEP3029	MZLULEP3029	*Polyommatus icarus*	Historical	Pinned Specimen (abdomen)	M	1940-07-30	Öland	Vickleby	56.5766	16.4604	Winblad, Gustaf	86.41	100	14.8130	16.7284
MZLULEP3133	MZLULEP3133	*Polyommatus icarus*	Historical	Pinned Specimen (abdomen)	M	1940-07-30	Öland	Vickleby	56.5766	16.4604	Winblad, Gustaf	87.84	100	4.2509	4.8654
MZLULEP3256	MZLULEP3256	*Polyommatus icarus*	Historical	Pinned Specimen (abdomen)	M	1940-07-30	Öland	Vickleby	56.5766	16.4604	Winblad, Gustaf	90.44	100	6.9215	7.5286
MZLULEP3259	MZLULEP3259	*Polyommatus icarus*	Historical	Pinned Specimen (abdomen)	M	1940-07-30	Öland	Vickleby	56.5766	16.4604	Winblad, Gustaf	86.78	100	4.2605	4.8570
MZLULEP3266	MZLULEP3266	*Polyommatus icarus*	Historical	Pinned Specimen (abdomen)	M	1940-07-30	Öland	Vickleby	56.5766	16.4604	Winblad, Gustaf	88.75	100	6.8630	7.7007
MZLULEP3313	MZLULEP3313	*Polyommatus icarus*	Historical	Pinned Specimen (abdomen)	M	1940-07-30	Öland	Vickleby	56.5766	16.4604	Winblad, Gustaf	86.47	100	4.9087	5.5662
MZLULEP3088	MZLULEP3088	*Polyommatus icarus*	Historical	Pinned Specimen (abdomen)	M	1940-08-01	Öland	Vickleby	56.5766	16.4604	Winblad, Gustaf	88.21	100	6.1489	6.9585
MZLULEP4555	MZLULEP4555	*Polyommatus icarus*	Historical	Pinned Specimen (abdomen)	M	1934-06-08	W Skåne	Södra sandby	55.7169	13.3472	Ander, Kjell	87.5	100	3.0313	3.3609
MZLULEP3679	MZLULEP3679	*Polyommatus icarus*	Historical	Pinned Specimen (abdomen)	M	1934-06-12	W Skåne	Billebjer	55.6883	13.3172	Ander, Kjell	90.1	100	8.6651	9.4829
MZLULEP4538	MZLULEP4538	*Polyommatus icarus*	Historical	Pinned Specimen (abdomen)	M	1934-06-24	W Skåne	Kungsmarken	55.7138	13.2753	Ander, Kjell	89.13	100	4.8134	5.2952
MZLULEP3661	MZLULEP3661	*Polyommatus icarus*	Historical	Pinned Specimen (abdomen)	M	1934-07-06	W Skåne	Kungsmarken	55.7138	13.2753	Ander, Kjell	88.41	100	3.5164	3.9514
MZLULEP1710	MZLULEP1710	*Polyommatus icarus*	Historical	Pinned Specimen (abdomen)	M	1934-08-02	W Skåne	Södra sandby	55.7169	13.3472	Nordström, FGD	86.05	100	5.0558	5.5778
MZLULEP1824	MZLULEP1824	*Polyommatus icarus*	Historical	Pinned Specimen (abdomen)	M	1934-08-02	W Skåne	Södra sandby	55.7169	13.3472	Nordström, FGD	90.33	100	3.3288	3.6008
MZLULEP1709	MZLULEP1709	*Polyommatus icarus*	Historical	Pinned Specimen (abdomen)	M	1934-08-20	W Skåne	Södra sandby	55.7169	13.3472	Nordström, FGD	86.77	99.99	5.6361	6.2998
MZLULEP1911	MZLULEP1911	*Polyommatus icarus*	Historical	Pinned Specimen (abdomen)	M	1934-08-20	W Skåne	Dalby	55.6648	13.3476	Nordström, FGD	64.56	99.93	3.6188	3.9413
MZLULEP3656	MZLULEP3656	*Polyommatus icarus*	Historical	Pinned Specimen (abdomen)	M	1934-08-31	W Skåne	Hällestad	55.6667	13.4167	Ander, Kjell	79.12	99.99	12.8158	14.2787
MZLULEP3668	MZLULEP3668	*Polyommatus icarus*	Historical	Pinned Specimen (abdomen)	M	1934-08-31	W Skåne	Hällestad	55.6667	13.4167	Ander, Kjell	88.73	99.99	4.0034	4.3815
MZLULEP4316	MZLULEP4316	*Polyommatus icarus*	Historical	Pinned Specimen (abdomen)	M	1934-08-31	W Skåne	Torna Hällestad	55.6781	13.4209	Ander, Kjell	63.95	99.92	3.1556	3.5317
PI200	MZLU00126549	*Polyommatus icarus*	Modern	Field Caught	M	2020-07-17	E Skåne	Haväng	55.7223	14.188	ZJ Nolen	99.45	96.54	25.8857	26.3887
PI201	MZLU00126550	*Polyommatus icarus*	Modern	Field Caught	M	2020-07-17	E Skåne	Haväng	55.7223	14.188	ZJ Nolen	99.48	96.61	25.6353	26.1393
PI202	MZLU00126551	*Polyommatus icarus*	Modern	Field Caught	M	2020-07-17	E Skåne	Haväng	55.7223	14.188	ZJ Nolen	99.42	96.51	26.5962	27.1144
PI203	MZLU00126552	*Polyommatus icarus*	Modern	Field Caught	M	2020-07-17	E Skåne	Haväng	55.7223	14.188	ZJ Nolen	99.48	96.62	23.8196	24.2916
PI204	MZLU00126553	*Polyommatus icarus*	Modern	Field Caught	M	2020-07-17	E Skåne	Haväng	55.7223	14.188	ZJ Nolen	99.46	96.58	30.1519	30.7385
PI205	MZLU00126554	*Polyommatus icarus*	Modern	Field Caught	M	2020-07-17	E Skåne	Haväng	55.7223	14.188	ZJ Nolen	98.8	96.08	21.0982	21.5142
PI206	MZLU00126555	*Polyommatus icarus*	Modern	Field Caught	M	2020-07-19	E Skåne	Haväng	55.7223	14.188	ZJ Nolen	96.93	94.69	25.7221	26.2343
PI207	MZLU00126556	*Polyommatus icarus*	Modern	Field Caught	M	2020-07-19	E Skåne	Haväng	55.7223	14.188	ZJ Nolen	99.03	96.35	26.4757	26.9919
PI208	MZLU00126557	*Polyommatus icarus*	Modern	Field Caught	M	2020-07-19	E Skåne	Haväng	55.7223	14.188	ZJ Nolen	98.83	96.05	26.9350	27.4674
PI413	MZLU00126558	*Polyommatus icarus*	Modern	Field Caught	M	2020-08-01	Öland	Sandby Borg	56.5532	16.6396	ZJ Nolen	99.4	96.02	44.1365	45.0132
PI414	MZLU00126559	*Polyommatus icarus*	Modern	Field Caught	M	2020-08-01	Öland	Sandby Borg	56.5532	16.6396	ZJ Nolen	99.41	95.96	47.9536	48.8774
PI415	MZLU00126560	*Polyommatus icarus*	Modern	Field Caught	M	2020-08-01	Öland	Sandby Borg	56.5532	16.6396	ZJ Nolen	99.38	95.99	55.6663	56.7365
PI417	MZLU00126561	*Polyommatus icarus*	Modern	Field Caught	M	2020-08-01	Öland	Sandby Borg	56.5532	16.6396	ZJ Nolen	99.39	96.05	49.5689	50.5379
PI418	MZLU00126562	*Polyommatus icarus*	Modern	Field Caught	M	2020-08-01	Öland	Sandby Borg	56.5532	16.6396	ZJ Nolen	99.07	95.61	45.7502	46.6659
PI419	MZLU00126563	*Polyommatus icarus*	Modern	Field Caught	M	2020-08-01	Öland	Sandby Borg	56.5532	16.6396	ZJ Nolen	99.4	96.14	43.7582	44.5946
PI420	MZLU00126564	*Polyommatus icarus*	Modern	Field Caught	M	2020-08-01	Öland	Sandby Borg	56.5532	16.6396	ZJ Nolen	99.39	96.02	38.3690	39.1139
PI421	MZLU00126565	*Polyommatus icarus*	Modern	Field Caught	M	2020-08-01	Öland	Sandby Borg	56.5532	16.6396	ZJ Nolen	99.41	95.96	45.4122	46.3059

PI422	MZLU00126566	*Polyommatus icarus*	Modern	Field Caught	M	2020-08-01	Öland	Sandby Borg	56.5532	16.6396	ZJ Nolen	99.4	95.99	52.7506	53.7786
PI028	MZLU00126531	*Polyommatus icarus*	Modern	Field Caught	M	2020-07-22	W Skåne	Tvedöra	55.6951	13.4316	ZJ Nolen	98.65	95.95	21.4433	21.8670
PI029	MZLU00126532	*Polyommatus icarus*	Modern	Field Caught	M	2020-07-22	W Skåne	Tvedöra	55.6951	13.4316	ZJ Nolen	99.45	96.42	16.6019	16.9345
PI030	MZLU00126533	*Polyommatus icarus*	Modern	Field Caught	M	2020-07-22	W Skåne	Tvedöra	55.6951	13.4316	ZJ Nolen	99.44	96.58	27.8268	28.3772
PI031	MZLU00126534	*Polyommatus icarus*	Modern	Field Caught	M	2020-07-22	W Skåne	Tvedöra	55.6951	13.4316	ZJ Nolen	99.36	96.53	24.9953	25.4878
PI032	MZLU00126535	*Polyommatus icarus*	Modern	Field Caught	M	2020-07-22	W Skåne	Tvedöra	55.6951	13.4316	ZJ Nolen	99.46	96.72	29.3019	29.8777
PI033	MZLU00126536	*Polyommatus icarus*	Modern	Field Caught	M	2020-07-22	W Skåne	Tvedöra	55.6951	13.4316	ZJ Nolen	99.46	96.53	23.5182	23.9842
PI034	MZLU00126537	*Polyommatus icarus*	Modern	Field Caught	M	2020-07-22	W Skåne	Tvedöra	55.6951	13.4316	ZJ Nolen	99.43	96.65	24.1715	24.6394
PI035	MZLU00126538	*Polyommatus icarus*	Modern	Field Caught	M	2020-07-22	W Skåne	Tvedöra	55.6951	13.4316	ZJ Nolen	99.27	96.45	26.6241	27.1400
PI036	MZLU00126539	*Polyommatus icarus*	Modern	Field Caught	M	2020-07-22	W Skåne	Tvedöra	55.6951	13.4316	ZJ Nolen	99.44	96.5	21.7631	22.1901
MZLU153003	MZLU153003	*Plebejus argus*	Historical	Pinned Specimen (abdomen)	M	1917-06-18	E Skåne	Åhus	55.9231	14.2942	Wahlgren, Einar	86.18	99.99	5.9404	6.5176
MZLU153166	MZLU153166	*Plebejus argus*	Historical	Pinned Specimen (abdomen)	M	1951-07-26	Öland	Gärdslösa	56.7842	16.7392	H. Rambring	88.25	100	6.7504	7.4776
MZLU153167	MZLU153167	*Plebejus argus*	Historical	Pinned Specimen (abdomen)	M	1951-07-26	Öland	Gärdslösa	56.7842	16.7392	H. Rambring	89.11	99.99	5.1508	5.6644
MZLU153168	MZLU153168	*Plebejus argus*	Historical	Pinned Specimen (abdomen)	M	1951-07-26	Öland	Gärdslösa	56.7842	16.7392	H. Rambring	87.21	100	5.5830	6.3410
MZLU153169	MZLU153169	*Plebejus argus*	Historical	Pinned Specimen (abdomen)	M	1951-07-26	Öland	Gärdslösa	56.7842	16.7392	H. Rambring	88.84	99.99	7.1448	7.7978
MZLU153170	MZLU153170	*Plebejus argus*	Historical	Pinned Specimen (abdomen)	M	1951-07-26	Öland	Gärdslösa	56.7842	16.7392	H. Rambring	87.86	99.99	7.6939	8.5635
MZLU153046	MZLU153046	*Plebejus argus*	Historical	Pinned Specimen (abdomen)	M	1936-06-27	Småland	Hjelmseryd	57.3112	14.5131	Carlgren G.	88.52	99.99	5.7848	6.5124
MZLU153040	MZLU153040	*Plebejus argus*	Historical	Pinned Specimen (abdomen)	M	1936-07-01	Småland	Hjelmseryd	57.3112	14.5131	Carlgren G.	88.43	99.99	4.3350	4.7956
MZLU153045	MZLU153045	*Plebejus argus*	Historical	Pinned Specimen (abdomen)	M	1936-07-06	Småland	Hjelmseryd	57.3112	14.5131	Carlgren G.	81.7	99.94	3.4038	3.7513
MZLU153042	MZLU153042	*Plebejus argus*	Historical	Pinned Specimen (abdomen)	M	1936-07-07	Småland	Hjelmseryd	57.3112	14.5131	Carlgren G.	88.85	100	4.9652	5.5416
MZLU153048	MZLU153048	*Plebejus argus*	Historical	Pinned Specimen (abdomen)	M	1936-07-12	Småland	Hjelmseryd	57.3112	14.5131	Carlgren G.	88.98	99.99	6.4863	7.1991
MZLU152940	MZLU152940	*Plebejus argus*	Historical	Pinned Specimen (abdomen)	M	1943-07-03	W Skåne	Hällestad	55.6765	13.4192	Ander, Kjell	89.71	100	5.4304	6.0053
PLAR0200	MZLU00126622	*Plebejus argus*	Modern	Field Caught	M	2022-07-06	E Skåne	Drakamöllan	55.7551	14.1252	Melina Eberhagen	99.24	96.28	16.3539	16.5749
PLAR0202	MZLU00126623	*Plebejus argus*	Modern	Field Caught	M	2022-07-06	E Skåne	Drakamöllan	55.7551	14.1252	Melina Eberhagen	99.23	96.33	11.5077	11.6594
PLAR0203	MZLU00126624	*Plebejus argus*	Modern	Field Caught	M	2022-07-06	E Skåne	Drakamöllan	55.7551	14.1252	Melina Eberhagen	99.23	96.15	10.1628	10.3002
PLAR0208	MZLU00126625	*Plebejus argus*	Modern	Field Caught	M	2022-07-06	E Skåne	Drakamöllan	55.7551	14.1252	Melina Eberhagen	99.2	96.06	8.8572	8.9760
PLAR0211	MZLU00126626	*Plebejus argus*	Modern	Field Caught	M	2022-07-06	E Skåne	Drakamöllan	55.7551	14.1252	Melina Eberhagen	99.21	96.31	10.1285	10.2634
PLAR0217	MZLU00126627	*Plebejus argus*	Modern	Field Caught	M	2022-07-06	E Skåne	Drakamöllan	55.7551	14.1252	Melina Eberhagen	99.22	96.2	11.8572	12.0063
PLAR0222	MZLU00126628	*Plebejus argus*	Modern	Field Caught	M	2022-07-06	E Skåne	Drakamöllan	55.7551	14.1252	Melina Eberhagen	99.21	96.14	10.6144	10.7523
PLAR0216	MZLU00126629	*Plebejus argus*	Modern	Field Caught	M	2022-07-06	E Skåne	Drakamöllan	55.7551	14.1252	Melina Eberhagen	99.23	99.23	11.1085	11.1087
MZLU107434	MZLU107434	*Plebejus argus*	Modern	Field Caught	M	2021-06-24	Småland	Götafors	57.492	14.1134	ZJ Nolen	99.26	95.36	18.5898	18.9344
MZLU107438	MZLU107438	*Plebejus argus*	Modern	Field Caught	M	2021-06-24	Småland	Götafors	57.492	14.1134	ZJ Nolen	99.24	94.79	24.3515	24.8322
MZLU107435	MZLU107435	*Plebejus argus*	Modern	Field Caught	M	2021-07-07	Småland	Götafors	57.492	14.1134	ZJ Nolen	99.24	94.76	27.5812	28.1041
MZLU107436	MZLU107436	*Plebejus argus*	Modern	Field Caught	M	2021-07-07	Småland	Götafors	57.492	14.1134	ZJ Nolen	99.26	95.16	16.6838	17.0067
MZLU107470	MZLU107470	*Plebejus argus*	Modern	Field Caught	M	2021-06-24	Småland	Götafors	57.492	14.1134	ZJ Nolen	99.26	95.08	20.6732	21.0737
MZLU107486	MZLU107486	*Plebejus argus*	Modern	Field Caught	M	2021-06-24	Småland	Götafors	57.492	14.1134	ZJ Nolen	99.25	94.65	10.6846	10.9039
MZLU107469	MZLU107469	*Plebejus argus*	Modern	Field Caught	M	2021-07-07	Småland	Götafors	57.492	14.1134	ZJ Nolen	99.26	94.79	18.7570	19.1297
MZLU107483	MZLU107483	*Plebejus argus*	Modern	Field Caught	M	2021-07-07	Småland	Götafors	57.492	14.1134	ZJ Nolen	98.95	94.41	6.9052	7.0452
MZLU107445	MZLU107445	*Plebejus argus*	Modern	Field Caught	M	2021-07-17	Öland	Jordtorpåsen	56.6783	16.5511	ZJ Nolen	99.08	94.52	30.0482	30.6303
MZLU107449	MZLU107449	*Plebejus argus*	Modern	Field Caught	M	2021-07-17	Öland	Jordtorpåsen	56.6783	16.5511	ZJ Nolen	99.28	95.25	20.3436	20.7343
MZLU107451	MZLU107451	*Plebejus argus*	Modern	Field Caught	M	2021-07-17	Öland	Jordtorpåsen	56.6783	16.5511	ZJ Nolen	99.19	95.05	19.6817	20.0461
MZLU107471	MZLU107471	*Plebejus argus*	Modern	Field Caught	M	2021-07-17	Öland	Jordtorpåsen	56.6783	16.5511	ZJ Nolen	99.27	94.9	24.9822	25.4670
MZLU107477	MZLU107477	*Plebejus argus*	Modern	Field Caught	M	2021-07-17	Öland	Jordtorpåsen	56.6783	16.5511	ZJ Nolen	99.15	94.4	12.7584	13.0254
MZLU107487	MZLU107487	*Plebejus argus*	Modern	Field Caught	M	2021-07-17	Öland	Jordtorpåsen	56.6783	16.5511	ZJ Nolen	99.25	94.85	11.9836	12.2250
MZLU107426	MZLU107426	*Plebejus argus*	Modern	Field Caught	M	2021-07-12	SW Skåne	Falsterbo	55.4025	12.8853	ZJ Nolen	99.26	95.29	18.9762	19.3367
MZLU107439	MZLU107439	*Plebejus argus*	Modern	Field Caught	M	2021-07-12	SW Skåne	Falsterbo	55.4025	12.8853	ZJ Nolen	99.21	94.96	19.4143	19.7905
MZLU107441	MZLU107441	*Plebejus argus*	Modern	Field Caught	M	2021-07-12	SW Skåne	Falsterbo	55.4025	12.8853	ZJ Nolen	99.25	95.11	16.0225	16.3322
MZLU107443	MZLU107443	*Plebejus argus*	Modern	Field Caught	M	2021-07-12	SW Skåne	Falsterbo	55.4025	12.8853	ZJ Nolen	99.26	95.07	30.3852	30.9579
MZLU107468	MZLU107468	*Plebejus argus*	Modern	Field Caught	M	2021-07-12	SW Skåne	Falsterbo	55.4025	12.8853	ZJ Nolen	99.25	95.23	15.0256	15.3127
MZLU107475	MZLU107475	*Plebejus argus*	Modern	Field Caught	M	2021-07-12	SW Skåne	Falsterbo	55.4025	12.8853	ZJ Nolen	99.09	95.08	32.3835	32.9918
MZLU107480	MZLU107480	*Plebejus argus*	Modern	Field Caught	M	2021-07-12	SW Skåne	Falsterbo	55.4025	12.8853	ZJ Nolen	99.24	94.45	10.2806	10.4898
MZLU153246	MZLU153246	*Cyaniris semiargus*	Historical	Pinned Specimen (abdomen)	M	1956-07-21	E Skåne	Degeberga	55.838	14.0901	H. Rambring	87.79	99.98	7.5046	7.9044
MZLU153247	MZLU153247	*Cyaniris semiargus*	Historical	Pinned Specimen (abdomen)	M	1956-07-21	E Skåne	Degeberga	55.838	14.0901	H. Rambring	91.55	100	8.3716	8.9663
MZLU153248	MZLU153248	*Cyaniris semiargus*	Historical	Pinned Specimen (abdomen)	M	1956-07-21	E Skåne	Degeberga	55.838	14.0901	H. Rambring	88.75	99.99	7.0833	7.4967

MZLU153251	MZLU153251	*Cyaniris semiargus*	Historical	Pinned Specimen (abdomen)	M	1956-07-21	E Skåne	Degeberga	55.838	14.0901	H. Rambring	87.62	99.99	8.2955	8.8308
MZLU153250	MZLU153250	*Cyaniris semiargus*	Historical	Pinned Specimen (abdomen)	M	1957-07-21	E Skåne	Degeberga	55.838	14.0901	H. Rambring	92.47	99.99	9.1029	9.5331
MZLU152770	MZLU152770	*Cyaniris semiargus*	Historical	Pinned Specimen (abdomen)	M	1936-06-27	Småland	Hjelmseryd	57.3112	14.5131	Carlgren G.	90.47	99.99	6.0121	6.4092
MZLU152769	MZLU152769	*Cyaniris semiargus*	Historical	Pinned Specimen (abdomen)	M	1936-06-29	Småland	Hjelmseryd	57.3112	14.5131	Carlgren G.	90.34	100	11.1979	12.4081
MZLU152765	MZLU152765	*Cyaniris semiargus*	Historical	Pinned Specimen (abdomen)	M	1936-07-01	Småland	Hjelmseryd	57.3112	14.5131	Carlgren G.	92.27	99.99	3.2783	3.4713
MZLU152766	MZLU152766	*Cyaniris semiargus*	Historical	Pinned Specimen (abdomen)	M	1936-07-07	Småland	Hjelmseryd	57.3112	14.5131	Carlgren G.	92.17	99.99	4.9600	5.2615
MZLU153204	MZLU153204	*Cyaniris semiargus*	Historical	Pinned Specimen (abdomen)	M	1951-06-19	W Skåne	Torna Hällestad	55.6781	13.4214	H. Rambring	92.39	100	7.6933	8.0408
MZLU153205	MZLU153205	*Cyaniris semiargus*	Historical	Pinned Specimen (abdomen)	M	1951-06-19	W Skåne	Torna Hällestad	55.6781	13.4214	H. Rambring	89.85	99.99	7.4680	7.8478
MZLU153206	MZLU153206	*Cyaniris semiargus*	Historical	Pinned Specimen (abdomen)	M	1951-06-19	W Skåne	Torna Hällestad	55.6781	13.4214	H. Rambring	92.53	99.99	6.8859	7.2077
MZLU153216	MZLU153216	*Cyaniris semiargus*	Historical	Pinned Specimen (abdomen)	M	1951-06-19	W Skåne	Torna Hällestad	55.6781	13.4214	H. Rambring	87.08	99.97	5.8776	6.1767
MZLU153221	MZLU153221	*Cyaniris semiargus*	Historical	Pinned Specimen (abdomen)	M	1951-06-19	W Skåne	Torna Hällestad	55.6781	13.4214	H. Rambring	93.52	100	5.2265	5.4991
MZLU107454	MZLU107454	*Cyaniris semiargus*	Modern	Field Caught	M	2021-07-03	E Skåne	Fästan	55.776	14.1722	ZJ Nolen; P Jamelska	99.61	97.68	18.1545	18.3237
MZLU107458	MZLU107458	*Cyaniris semiargus*	Modern	Field Caught	M	2021-07-03	E Skåne	Fästan	55.776	14.1722	ZJ Nolen; P Jamelska	99.64	97.53	17.6309	17.7944
MZLU107463	MZLU107463	*Cyaniris semiargus*	Modern	Field Caught	M	2021-07-03	E Skåne	Fästan	55.776	14.1722	ZJ Nolen; P Jamelska	99.58	97.6	18.1059	18.2741
MZLU107466	MZLU107466	*Cyaniris semiargus*	Modern	Field Caught	M	2021-07-03	E Skåne	Fästan	55.776	14.1722	ZJ Nolen; P Jamelska	99.64	97.58	19.5090	19.6931
MZLU107503	MZLU107503	*Cyaniris semiargus*	Modern	Field Caught	M	2021-07-03	E Skåne	Fästan	55.776	14.1722	ZJ Nolen; P Jamelska	99.51	97.64	9.0339	9.1190
MZLU107504	MZLU107504	*Cyaniris semiargus*	Modern	Field Caught	M	2021-07-03	E Skåne	Fästan	55.776	14.1722	ZJ Nolen; P Jamelska	99.66	97.73	14.3537	14.4856
MZLU107506	MZLU107506	*Cyaniris semiargus*	Modern	Field Caught	M	2021-07-03	E Skåne	Fästan	55.776	14.1722	ZJ Nolen; P Jamelska	99.53	97.55	13.2708	13.3974
MZLU107507	MZLU107507	*Cyaniris semiargus*	Modern	Field Caught	M	2021-07-03	E Skåne	Fästan	55.776	14.1722	ZJ Nolen; P Jamelska	99.62	97.62	14.4395	14.5770
CYSE0078	MZLU00107520	*Cyaniris semiargus*	Modern	Field Caught	M	2022-06-23	SE Skåne	SE Skåne	55.3864	14.1014	Melina Eberhagen	99.64	98.09	15.2662	15.3476
CYSE0094	MZLU00107522	*Cyaniris semiargus*	Modern	Field Caught	M	2022-06-27	SE Skåne	SE Skåne	55.3864	14.1014	Melina Eberhagen	97.84	96.82	12.8535	12.9280
CYSE0096	MZLU00107523	*Cyaniris semiargus*	Modern	Field Caught	M	2022-06-27	SE Skåne	SE Skåne	55.3864	14.1014	Melina Eberhagen	99.22	97.76	7.3142	7.3457
CYSE0098	MZLU00107524	*Cyaniris semiargus*	Modern	Field Caught	M	2022-06-27	SE Skåne	SE Skåne	55.3864	14.1014	Melina Eberhagen	99.64	97.99	12.7827	12.8535
CYSE0116	MZLU00107525	*Cyaniris semiargus*	Modern	Field Caught	M	2022-06-28	SE Skåne	SE Skåne	55.3864	14.1014	Melina Eberhagen	99.62	98.14	10.8479	10.9059
CYSE0198	MZLU00107526	*Cyaniris semiargus*	Modern	Field Caught	F	2022-07-01	SE Skåne	SE Skåne	55.3864	14.1014	Melina Eberhagen	99.48	97.78	13.4596	13.5280
CYSE0199	MZLU00107527	*Cyaniris semiargus*	Modern	Field Caught	F	2022-07-01	SE Skåne	SE Skåne	55.3864	14.1014	Melina Eberhagen	99.44	97.81	10.7599	10.8094
MZLU107461	MZLU107461	*Cyaniris semiargus*	Modern	Field Caught	F	2021-06-24	Småland	Götafors	57.492	14.1134	ZJ Nolen; P Jamelska	99.5	97.27	15.5624	15.6980
MZLU107462	MZLU107462	*Cyaniris semiargus*	Modern	Field Caught	F	2021-06-24	Småland	Götafors	57.492	14.1134	ZJ Nolen; P Jamelska	99.55	97.55	20.3960	20.5832
MZLU107465	MZLU107465	*Cyaniris semiargus*	Modern	Field Caught	M	2021-06-24	Småland	Götafors	57.492	14.1134	ZJ Nolen; P Jamelska	99.67	97.75	24.9321	25.1610
MZLU107455	MZLU107455	*Cyaniris semiargus*	Modern	Field Caught	M	2021-07-07	Småland	Götafors	57.492	14.1134	ZJ Nolen; P Jamelska	99.66	97.61	13.2588	13.3896
MZLU107456	MZLU107456	*Cyaniris semiargus*	Modern	Field Caught	M	2021-07-07	Småland	Götafors	57.492	14.1134	ZJ Nolen; P Jamelska	99.64	97.58	11.1263	11.2295
MZLU107501	MZLU107501	*Cyaniris semiargus*	Modern	Field Caught	F	2021-07-07	Småland	Götafors	57.492	14.1134	ZJ Nolen; P Jamelska	99.52	97.26	10.9493	11.0538
MZLU107457	MZLU107457	*Cyaniris semiargus*	Modern	Field Caught	M	2021-07-04	W Skåne	Silvåkra	55.6695	13.4924	ZJ Nolen; P Jamelska	99.66	97.71	19.0891	19.2644
MZLU107460	MZLU107460	*Cyaniris semiargus*	Modern	Field Caught	M	2021-07-04	W Skåne	Silvåkra	55.6695	13.4924	ZJ Nolen; P Jamelska	99.64	97.71	15.4684	15.6090
MZLU107464	MZLU107464	*Cyaniris semiargus*	Modern	Field Caught	M	2021-07-04	W Skåne	Silvåkra	55.6695	13.4924	ZJ Nolen; P Jamelska	99.55	97.62	15.2425	15.3885
MZLU107497	MZLU107497	*Cyaniris semiargus*	Modern	Field Caught	M	2021-07-04	W Skåne	Silvåkra	55.6695	13.4924	ZJ Nolen; P Jamelska	94.21	93.41	11.5859	11.6982
MZLU107498	MZLU107498	*Cyaniris semiargus*	Modern	Field Caught	M	2021-07-04	W Skåne	Silvåkra	55.6695	13.4924	ZJ Nolen; P Jamelska	99.37	97.37	12.1112	12.2275
MZLU107499	MZLU107499	*Cyaniris semiargus*	Modern	Field Caught	M	2021-07-04	W Skåne	Silvåkra	55.6695	13.4924	ZJ Nolen; P Jamelska	99.66	97.8	12.8861	13.0066
MZLU107500	MZLU107500	*Cyaniris semiargus*	Modern	Field Caught	M	2021-07-04	W Skåne	Silvåkra	55.6695	13.4924	ZJ Nolen; P Jamelska	99.56	97.31	9.5916	9.6883
MZLU107505	MZLU107505	*Cyaniris semiargus*	Modern	Field Caught	M	2021-07-04	W Skåne	Silvåkra	55.6695	13.4924	ZJ Nolen; P Jamelska	99.13	97.1	12.6073	12.7307
MZLU166853	MZLU166853	*Agriades optilete*	Historical	Pinned Specimen (abdomen)	M	1961-06-29	Småland	Agunnaryd	56.7318	14.2439	Nils Burrau	NA	100	NA	7.7865
MZLU107492	MZLU107492	*Agriades optilete*	Modern	Field Caught	M	2021-07-05	Småland	Agunnaryd	56.7318	14.2439	ZJ Nolen	NA	98.23	NA	12.211
MZLU166851	MZLU166851	*Aricia agestis*	Historical	Pinned Specimen (abdomen)	M	1939-xx-xx	W Skåne	Vomb	55.6731	13.5503	Nils Burrau	NA	100	NA	5.8508
MZLU107489	MZLU107489	*Aricia agestis*	Modern	Field Caught	M	2020-08-03	W Skåne	Tvedöra	55.6959	13.431	ZJ Nolen	NA	94.13	NA	7.5821
MZLU166852	MZLU166852	*Cupido minimus*	Historical	Pinned Specimen (abdomen)	M	1943-05-30	W Skåne	Torna Hällestad	55.6781	13.4214	Nils Burrau	NA	99.99	NA	6.2958
CUMI0012	NA	*Cupido minimus*	Modern	Field Caught	M	2022-06-14	W Skåne	Tvedöra	55.6959	13.431	ZJ Nolen	NA	96.21	NA	7.7405
EK001	MZLU161410	*Plebejus argyrognomon*	Historical	Pinned Specimen (abdomen)	M	1967-07-18	W Skåne	Dalhem	56.0675	12.7367	Harry Rydén	NA	99.97	NA	9.6382
EK006	private collection	*Plebejus argyrognomon*	Modern	Pinned Specimen (legs)	M	2003-07-18	W Skåne	Dalhem	56.0675	12.7367	Göran Engqvist	NA	99.99	NA	13.8387
MZLU174142	MZLU174142	*Scolitantides orion*	Historical	Pinned Specimen (abdomen)	M	1944-xx-08	Södermanland	Stockholm	59.3294	18.0686	E. Norstrand	NA	100	NA	7.779
MZLU174140	MZLU174140	*Scolitantides orion*	Modern	Pinned Specimen (abdomen)	M	2016-xx-xx	Södermanland	Långaedebergen	59.2154	17.1414	Håkan Elmqvist	NA	100	NA	8.4804

Table S2. Individual estimates of heterozygosity and inbreeding (Froh). Individual heterozygosity was estimated from the filtered genotypes, called with data subsampled to a uniform 6x coverage. We estimate heterozygosity as a count of heterozygous genotypes per 1000 genotypes, as well as a count per alternate allele as a potential correction for reference biases. These two estimates largely agreed with each other. Froh is the inbreeding coefficient from runs of homozygosity, estimated as the proportion of the autosomal genome in runs of homozygosity longer than 200kb. This analysis was performed using BCFtools RoH on called genotypes. For both analyses, transition sites were removed. For heterozygosity this means that the values are lower than true heterozygosity in the samples, instead representing only transversion heterozygous sites per 1000bp. Froh values should not be affected by transition removal in this same way, as it is based on ranges across multiple variants, not variant counts. Finally, we include an estimate of heterozygosity adjusted by Froh to approximate heterozygosity outside of runs of homozygosity. Much of the variation in heterozygosity between individuals is explained by variation in Froh, as can be seen by the reduced variance in this statistic.

Sample ID	Species	Time Period	Study Region	Heterozygous sites / 1000bp (excluding transitions; 6x depth)	Heterozygous sites / called alternate allele (excluding transitions; 6x depth)	Froh > 200kb (6x depth)	Heterozygosity / (1-Froh)
MZLULEP1724	*Polyommatus icarus*	Historical	E Skåne	3.733	0.467	0.000	3.733
MZLULEP1661	*Polyommatus icarus*	Historical	E Skåne	3.732	0.469	0.001	3.734
MZLULEP1903	*Polyommatus icarus*	Historical	E Skåne	3.731	0.469	0.000	3.731
MZLULEP3086	*Polyommatus icarus*	Historical	Öland	3.728	0.464	0.000	3.728
MZLULEP3149	*Polyommatus icarus*	Historical	Öland	3.702	0.464	0.001	3.706
MZLULEP3328	*Polyommatus icarus*	Historical	Öland	3.744	0.465	0.004	3.757
MZLULEP3029	*Polyommatus icarus*	Historical	Öland	3.728	0.465	0.001	3.732
MZLULEP3256	*Polyommatus icarus*	Historical	Öland	3.656	0.463	0.000	3.656
MZLULEP3266	*Polyommatus icarus*	Historical	Öland	3.819	0.469	0.000	3.819
MZLULEP3313	*Polyommatus icarus*	Historical	Öland	3.632	0.458	0.001	3.637
MZLULEP3088	*Polyommatus icarus*	Historical	Öland	3.973	0.471	0.003	3.986
MZLULEP3679	*Polyommatus icarus*	Historical	W Skåne	3.690	0.465	0.002	3.698
MZLULEP4538	*Polyommatus icarus*	Historical	W Skåne	3.602	0.459	0.000	3.602
MZLULEP1710	*Polyommatus icarus*	Historical	W Skåne	3.938	0.471	0.001	3.940
MZLULEP1709	*Polyommatus icarus*	Historical	W Skåne	3.873	0.472	0.000	3.873
MZLULEP3656	*Polyommatus icarus*	Historical	W Skåne	3.815	0.471	0.001	3.819
PI200	*Polyommatus icarus*	Modern	E Skåne	3.781	0.470	0.000	3.781
PI201	*Polyommatus icarus*	Modern	E Skåne	3.808	0.472	0.000	3.808
PI202	*Polyommatus icarus*	Modern	E Skåne	3.782	0.471	0.000	3.784
PI203	*Polyommatus icarus*	Modern	E Skåne	3.764	0.469	0.001	3.767
PI204	*Polyommatus icarus*	Modern	E Skåne	3.775	0.471	0.001	3.778
PI205	*Polyommatus icarus*	Modern	E Skåne	3.782	0.471	0.000	3.782
PI206	*Polyommatus icarus*	Modern	E Skåne	3.768	0.470	0.000	3.768
PI207	*Polyommatus icarus*	Modern	E Skåne	3.789	0.470	0.000	3.789
PI208	*Polyommatus icarus*	Modern	E Skåne	3.777	0.470	0.000	3.777
PI413	*Polyommatus icarus*	Modern	Öland	3.741	0.465	0.000	3.741
PI414	*Polyommatus icarus*	Modern	Öland	3.718	0.463	0.000	3.718
PI415	*Polyommatus icarus*	Modern	Öland	3.753	0.466	0.000	3.753
PI417	*Polyommatus icarus*	Modern	Öland	3.729	0.465	0.001	3.734
PI418	*Polyommatus icarus*	Modern	Öland	3.728	0.465	0.000	3.728
PI419	*Polyommatus icarus*	Modern	Öland	3.697	0.462	0.000	3.697
PI420	*Polyommatus icarus*	Modern	Öland	3.707	0.463	0.001	3.712
PI421	*Polyommatus icarus*	Modern	Öland	3.728	0.463	0.001	3.731
PI422	*Polyommatus icarus*	Modern	Öland	3.722	0.465	0.000	3.722
PI028	*Polyommatus icarus*	Modern	W Skåne	3.783	0.471	0.000	3.784
PI029	*Polyommatus icarus*	Modern	W Skåne	3.765	0.469	0.001	3.768
PI030	*Polyommatus icarus*	Modern	W Skåne	3.776	0.472	0.000	3.776
PI031	*Polyommatus icarus*	Modern	W Skåne	3.815	0.472	0.008	3.846
PI032	*Polyommatus icarus*	Modern	W Skåne	3.755	0.469	0.000	3.755
PI033	*Polyommatus icarus*	Modern	W Skåne	3.777	0.469	0.014	3.832
PI034	*Polyommatus icarus*	Modern	W Skåne	3.774	0.470	0.000	3.774
PI035	*Polyommatus icarus*	Modern	W Skåne	3.783	0.471	0.000	3.783
PI036	*Polyommatus icarus*	Modern	W Skåne	3.758	0.468	0.000	3.758
MZLU153003	*Plebejus argus*	Historical	E Skåne	2.623	0.448	0.001	2.627
MZLU153166	*Plebejus argus*	Historical	Öland	2.606	0.445	0.003	2.613
MZLU153167	*Plebejus argus*	Historical	Öland	2.566	0.442	0.004	2.576
MZLU153168	*Plebejus argus*	Historical	Öland	2.590	0.441	0.006	2.606
MZLU153169	*Plebejus argus*	Historical	Öland	2.591	0.445	0.003	2.597
MZLU153170	*Plebejus argus*	Historical	Öland	2.585	0.443	0.008	2.604

MZLU153046	*Plebejus argus*	Historical	Småland	2.593	0.447	0.000	2.593
MZLU153042	*Plebejus argus*	Historical	Småland	2.552	0.436	0.006	2.568
MZLU153048	*Plebejus argus*	Historical	Småland	2.522	0.431	0.030	2.599
MZLU152940	*Plebejus argus*	Historical	W Skåne	2.564	0.440	0.016	2.605
PLAR0200	*Plebejus argus*	Modern	E Skåne	2.619	0.444	0.005	2.631
PLAR0202	*Plebejus argus*	Modern	E Skåne	2.629	0.447	0.000	2.629
PLAR0203	*Plebejus argus*	Modern	E Skåne	2.623	0.444	0.001	2.624
PLAR0208	*Plebejus argus*	Modern	E Skåne	2.626	0.445	0.002	2.630
PLAR0211	*Plebejus argus*	Modern	E Skåne	2.580	0.439	0.012	2.611
PLAR0217	*Plebejus argus*	Modern	E Skåne	2.625	0.445	0.001	2.629
PLAR0222	*Plebejus argus*	Modern	E Skåne	2.612	0.443	0.006	2.628
PLAR0226	*Plebejus argus*	Modern	E Skåne	2.586	0.446	0.002	2.590
MZLU107434	*Plebejus argus*	Modern	Öland	2.577	0.439	0.009	2.599
MZLU107438	*Plebejus argus*	Modern	Öland	2.572	0.437	0.012	2.604
MZLU107435	*Plebejus argus*	Modern	Öland	2.593	0.440	0.007	2.612
MZLU107436	*Plebejus argus*	Modern	Öland	2.491	0.423	0.040	2.596
MZLU107445	*Plebejus argus*	Modern	Öland	2.504	0.429	0.028	2.577
MZLU107449	*Plebejus argus*	Modern	Öland	2.558	0.435	0.012	2.590
MZLU107451	*Plebejus argus*	Modern	Öland	2.590	0.442	0.001	2.591
MZLU107471	*Plebejus argus*	Modern	Öland	2.558	0.435	0.012	2.589
MZLU107477	*Plebejus argus*	Modern	Öland	2.574	0.438	0.002	2.580
MZLU107487	*Plebejus argus*	Modern	Öland	2.497	0.425	0.029	2.572
MZLU107470	*Plebejus argus*	Modern	Småland	2.594	0.441	0.001	2.597
MZLU107486	*Plebejus argus*	Modern	Småland	2.558	0.435	0.019	2.608
MZLU107469	*Plebejus argus*	Modern	Småland	2.582	0.440	0.000	2.582
MZLU107483	*Plebejus argus*	Modern	Småland	2.571	0.437	0.011	2.601
MZLU107426	*Plebejus argus*	Modern	SW Skåne	2.600	0.440	0.009	2.624
MZLU107439	*Plebejus argus*	Modern	SW Skåne	2.584	0.438	0.020	2.638
MZLU107441	*Plebejus argus*	Modern	SW Skåne	2.565	0.435	0.017	2.609
MZLU107443	*Plebejus argus*	Modern	SW Skåne	2.584	0.438	0.009	2.608
MZLU107468	*Plebejus argus*	Modern	SW Skåne	2.606	0.440	0.007	2.624
MZLU107475	*Plebejus argus*	Modern	SW Skåne	2.616	0.444	0.000	2.616
MZLU107480	*Plebejus argus*	Modern	SW Skåne	2.602	0.441	0.007	2.619
MZLU153246	*Cyaniris semiargus*	Historical	E Skåne	2.283	0.434	0.004	2.293
MZLU153247	*Cyaniris semiargus*	Historical	E Skåne	2.299	0.432	0.003	2.305
MZLU153248	*Cyaniris semiargus*	Historical	E Skåne	2.282	0.432	0.004	2.292
MZLU153251	*Cyaniris semiargus*	Historical	E Skåne	2.221	0.417	0.031	2.291
MZLU153250	*Cyaniris semiargus*	Historical	E Skåne	2.291	0.435	0.000	2.291
MZLU152770	*Cyaniris semiargus*	Historical	Småland	2.067	0.398	0.014	2.097
MZLU152769	*Cyaniris semiargus*	Historical	Småland	2.150	0.409	0.003	2.156
MZLU152766	*Cyaniris semiargus*	Historical	Småland	2.065	0.406	0.001	2.066
MZLU153204	*Cyaniris semiargus*	Historical	W Skåne	2.208	0.421	0.026	2.267
MZLU153205	*Cyaniris semiargus*	Historical	W Skåne	2.283	0.434	0.003	2.289
MZLU153206	*Cyaniris semiargus*	Historical	W Skåne	2.248	0.430	0.009	2.270
MZLU153216	*Cyaniris semiargus*	Historical	W Skåne	2.282	0.434	0.000	2.282
MZLU153221	*Cyaniris semiargus*	Historical	W Skåne	2.224	0.429	0.002	2.228
MZLU107454	*Cyaniris semiargus*	Modern	E Skåne	2.234	0.425	0.008	2.251
MZLU107458	*Cyaniris semiargus*	Modern	E Skåne	2.197	0.418	0.022	2.247
MZLU107463	*Cyaniris semiargus*	Modern	E Skåne	2.195	0.417	0.032	2.269
MZLU107466	*Cyaniris semiargus*	Modern	E Skåne	2.238	0.426	0.006	2.253
MZLU107503	*Cyaniris semiargus*	Modern	E Skåne	2.229	0.423	0.023	2.283
MZLU107504	*Cyaniris semiargus*	Modern	E Skåne	2.236	0.426	0.015	2.270
MZLU107506	*Cyaniris semiargus*	Modern	E Skåne	2.226	0.424	0.014	2.258
MZLU107507	*Cyaniris semiargus*	Modern	E Skåne	2.237	0.425	0.008	2.256
CYSE0078	*Cyaniris semiargus*	Modern	SE Skåne	1.533	0.284	0.329	2.283
CYSE0094	*Cyaniris semiargus*	Modern	SE Skåne	1.852	0.347	0.185	2.274
CYSE0096	*Cyaniris semiargus*	Modern	SE Skåne	2.013	0.378	0.118	2.282
CYSE0098	*Cyaniris semiargus*	Modern	SE Skåne	1.962	0.367	0.142	2.287
CYSE0116	*Cyaniris semiargus*	Modern	SE Skåne	2.062	0.389	0.092	2.270
CYSE0198	*Cyaniris semiargus*	Modern	SE Skåne	2.104	0.396	0.074	2.272
CYSE0199	*Cyaniris semiargus*	Modern	SE Skåne	1.963	0.368	0.132	2.263
MZLU107461	*Cyaniris semiargus*	Modern	Småland	2.160	0.408	0.018	2.198

MZLU107462	*Cyaniris semiargus*	Modern	Småland	2.162	0.412	0.001	2.164
MZLU107465	*Cyaniris semiargus*	Modern	Småland	1.806	0.339	0.170	2.176
MZLU107455	*Cyaniris semiargus*	Modern	Småland	2.177	0.412	0.002	2.183
MZLU107456	*Cyaniris semiargus*	Modern	Småland	1.821	0.344	0.146	2.133
MZLU107501	*Cyaniris semiargus*	Modern	Småland	2.008	0.378	0.072	2.165
MZLU107457	*Cyaniris semiargus*	Modern	W Skåne	2.137	0.403	0.060	2.273
MZLU107460	*Cyaniris semiargus*	Modern	W Skåne	2.159	0.408	0.057	2.290
MZLU107464	*Cyaniris semiargus*	Modern	W Skåne	2.206	0.417	0.029	2.273
MZLU107497	*Cyaniris semiargus*	Modern	W Skåne	2.109	0.397	0.075	2.279
MZLU107498	*Cyaniris semiargus*	Modern	W Skåne	2.206	0.417	0.036	2.288
MZLU107499	*Cyaniris semiargus*	Modern	W Skåne	2.039	0.382	0.119	2.315
MZLU107500	*Cyaniris semiargus*	Modern	W Skåne	2.141	0.402	0.067	2.294
MZLU107505	*Cyaniris semiargus*	Modern	W Skåne	2.179	0.412	0.047	2.287
MZLU166853	*Agriades optilete*	Historical	Småland	0.790	0.342	0.005	0.794
MZLU107492	*Agriades optilete*	Modern	Småland	0.796	0.343	0.002	0.797
MZLU166851	*Aricia agestis*	Historical	W Skåne	2.507	0.331	0.089	2.751
MZLU107489	*Aricia agestis*	Modern	W Skåne	2.691	0.348	0.061	2.865
MZLU166852	*Cupido minimus*	Historical	W Skåne	2.225	0.354	0.065	2.380
CUMI0012	*Cupido minimus*	Modern	W Skåne	2.324	0.360	0.087	2.544
EK001	*Plebejus argyrognomon*	Historical	W Skåne	1.559	0.057	0.038	1.621
EK006	*Plebejus argyrognomon*	Modern	W Skåne	1.461	0.053	0.078	1.585
MZLU174142	*Scolitantides orion*	Historical	Södermanland	0.275	0.053	0.136	0.318
MZLU174140	*Scolitantides orion*	Modern	Södermanland	0.176	0.034	0.294	0.249

Table S3. Statistical testing of differences in heterozygosity between modern and historical samples. We statistically assessed differences in heterozygosity between modern and historical samples for each species using a linear mixed model with heterozygosity as a response to the fixed effect of time period and the random effect of sampling region. Outputs from the models are summarized here.

Species	Mean heterozyg. (hist)	Mean heterozyg. (hist, jackknife CI)	Mean heterozyg. (mod)	Mean heterozyg. (mod, jackknife CI)	Heterozyg. mod/hist	Difference est.	Std.Error	t-statistic	95% CI (lower)	95% CI (upper)	p-value
Po. icarus	3.756	[3.74242, 3.76553]	3.7606	[3.75868, 3.76279]	1.00123	0.0019	0.0209	0.0907	-0.04038	0.04417	0.9282
Pl. argus	2.57904	[2.57458, 2.58464]	2.58191	[2.58031, 2.58501]	1.00111	-0.0083	0.0109	-0.76148	-0.03045	0.01385	0.45162
Cy. semiargus	2.22325	[2.21716, 2.23636]	2.08933	[2.08404, 2.10238]	0.93976	-0.13302	0.04753	-2.79842	-0.22925	-0.03679	**0.00802**

Table S4. Estimates of pairwise nucleotide diversity for populations of focal species. For each focal species population sample, we estimated nucleotide diversity (pi) in 50kb non-overlapping sliding windows using the method implemented in Pixy. We estimated mean nucleotide diversity as an average of all windows with confidence intervals estimated by performing 10 000 bootstraps, resampling windows with replacement.

Species	Region	Time Period	Year	Mean Nucleotide Diversity	95% CI (Lower)	95% CI (Upper)
Po. icarus	E Skåne	Historical	1934	0.003891	0.003868	0.003915
Po. icarus	E Skåne	Modern	2020	0.003960	0.003939	0.003982
Po. icarus	Öland	Historical	1940	0.003947	0.003925	0.003969
Po. icarus	Öland	Modern	2020	0.003923	0.003902	0.003945
Po. icarus	W Skåne	Historical	1934	0.003999	0.003976	0.004021
Po. icarus	W Skåne	Modern	2020	0.003953	0.003930	0.003976
Pl. argus	E Skåne	Modern	2022	0.002771	0.002750	0.002793
Pl. argus	Öland	Historical	1951	0.002758	0.002735	0.002781
Pl. argus	Öland	Modern	2021	0.002716	0.002694	0.002738
Pl. argus	Småland	Historical	1936	0.002750	0.002727	0.002773
Pl. argus	Småland	Modern	2021	0.002711	0.002688	0.002733
Pl. argus	SW Skåne	Modern	2021	0.002765	0.002742	0.002787
Cy. semiargus	E Skåne	Historical	1956	0.002389	0.002373	0.002405
Cy. semiargus	E Skåne	Modern	2021	0.002345	0.002322	0.002377
Cy. semiargus	SE Skåne	Modern	2022	0.002091	0.002069	0.002118
Cy. semiargus	Småland	Historical	1936	0.002191	0.002175	0.002208
Cy. semiargus	Småland	Modern	2021	0.002175	0.002149	0.002209
Cy. semiargus	W Skåne	Historical	1951	0.002300	0.002280	0.002324
Cy. semiargus	W Skåne	Modern	2021	0.002252	0.002236	0.002268

Table S5. Change in nucleotide diversity over the sampled time period and projected to 100 years. Only comparisons between geographically proximal (<40km) sampling sites across time periods are shown. Proportion nucleotide diversity retained represents the modern population nucleotide diversity over the historical population nucleotide diversity. This proportion is extrapolated to a yearly rate of change which is then standardized to 100 years and placed into a threat category using the indicators outlined by Andersson et al. (28).

Species	Modern Region	Modern Year	Historical Region	Historical Year	Proportion retained	Proportion retained / year	% retained per 100 years	Threat category
P. icarus	E Skåne	2020	E Skåne	1934	1.0177	1.0002	102.06	Acceptable
P. icarus	Öland	2020	Öland	1940	0.9940	0.9999	99.25	Acceptable
P. icarus	W Skåne	2020	W Skåne	1934	0.9885	0.9999	98.66	Acceptable
P. argus	Öland	2021	Öland	1951	0.9848	0.9998	97.84	Acceptable
P. argus	Småland	2021	Småland	1936	0.9858	0.9998	98.33	Acceptable
C. semiargus	E Skåne	2021	E Skåne	1956	0.9817	0.9997	97.20	Acceptable
C. semiargus	SE Skåne	2022	E Skåne	1956	0.8752	0.9980	81.71	Warning
C. semiargus	Småland	2021	Småland	1936	0.9924	0.9999	99.10	Acceptable
C. semiargus	W Skåne	2021	W Skåne	1951	0.9792	0.9997	97.04	Acceptable

Table S6. Predicted retention of heterozygosity over 100 years per species and estimates of contemporary effective population size. We estimated the predicted percent of heterozygosity retained over 100 years, following Andersson et al. (2022) for each of the study species. For the focal species where we had multiple samples per populations, we first estimated it using the mean population heterozygosity, inferring a yearly rate of heterozygosity retention per population and projecting it out to 100 years. As the additional species only included one sample per time period, we estimated this rate with all possible historical and modern individual pairs for a region. For supplementray species, this results in a single number, for the three focal species, we report the median and range values. We assigned a threat category to each of the median estimates, based on the thresholds described by Andersson et al. For the focal species, we estimated recent trajectories of effective population size with GONE, reporting the size at generation 5 here and assessing this value under the indicator from Andersson et al.

			Population means		*Pairwise between individuals*		*Contemporary Ne*	
**Species**	**Study Region**	**2020 Swedish Red List**	**% heterozygosity retained over 100 years**	**Threat Categorization**	**Median % heterozygosity retained over 100 years (range)**	**Threat Categorization**	**Effective population size**	**Threat Categorization**
Aricia agestis	W Skåne	LC	NA	NA	109.02	Acceptable	NA	NA
Agriades optilete	Småland	LC	NA	NA	101.22	Acceptable	NA	NA
Cupido minimus	W Skåne	NT	NA	NA	105.64	Acceptable	NA	NA
Cyaniris semiargus	E Skåne	LC	96.58	Acceptable	96.36 (93.17-101.22)	Acceptable	739.802	Acceptable
Cyaniris semiargus	E Skåne (SE)	LC	77.76	Warning	79.64 (54.13-92.15)	Warning	88.0539	Warning
Cyaniris semiargus	Småland	LC	95.98	Acceptable	98.65 (81.46-106.42)	Acceptable	44224.5	Acceptable
Cyaniris semiargus	W Skåne	LC	93.57	Warning	93.99 (85.13-99.87)	Warning	447.059	Warning
Plebejus argus	E Skåne	LC	99.61	Acceptable	99.91 (98.45-100.2)	Acceptable	3.46E+06	Acceptable
Plebejus argus	Öland	LC	97.76	Acceptable	98.21 (94.08-101.34)	Acceptable	1017.42	Acceptable
Plebejus argus	Småland	LC	100.54	Acceptable	100.89 (95.41-103.39)	Acceptable	545.272	Acceptable
Plebejus argus	W Skåne	LC	101.52	Acceptable	101.84 (100.08-102.65)	Acceptable	231331	Acceptable
Plebejus argyrognomon	W Skåne	CR	NA	NA	87.89	Warning	NA	NA
Polyommatus icarus	E Skåne	LC	101.52	Acceptable	101.52 (100.97-102.4)	Acceptable	2421.84	Acceptable
Polyommatus icarus	Öland	LC	99.23	Acceptable	99.99 (91.38-104.16)	Acceptable	2.53E+06	Acceptable
Polyommatus icarus	W Skåne	LC	99.78	Acceptable	98.8 (94.62-106.94)	Acceptable	213594	Acceptable
Scolitantides orion	Södermanland	EN	NA	NA	53.80	Alarm	NA	NA

Table S7. Genetic differentiation between populations of the focal species. We estimated genetic differentiation using pairwise *F*_ ST between populations within each time period, using the Hudson estimator as implemented in Pixy. We estimated *F*_ ST in 50kb non-overlapping windows, estimating the mean across all windows and 95% confidence intervals with 10 000 bootstraps, resampling windows with replacement.

Species	Region 1	Region 1 Year	Region 2	Region 2 Year	Time Period	Distance (km)	Mean Hudson Fst	95% CI (Lower)	95% CI (Upper)
P. icarus	E Skåne	1934	W Skåne	1934	Historical	50.01	0.01829	0.01776	0.01887
P. icarus	E Skåne	1934	Öland	1940	Historical	170.71	0.02103	0.02047	0.02161
P. icarus	Öland	1940	W Skåne	1934	Historical	212.33	0.01547	0.01503	0.01590
P. icarus	E Skåne	2020	W Skåne	2020	Modern	47.65	0.00818	0.00795	0.00843
P. icarus	E Skåne	2020	Öland	2020	Modern	178.28	0.01241	0.01207	0.01275
P. icarus	Öland	2020	W Skåne	2020	Modern	221.18	0.01366	0.01330	0.01403
P. argus	Småland	1936	Öland	1951	Historical	147.3	0.03497	0.03386	0.03612
P. argus	E Skåne	2022	SW Skåne	2021	Modern	87.5	0.02665	0.02589	0.02743
P. argus	Småland	2021	Öland	2021	Modern	173.36	0.03760	0.03659	0.03863
P. argus	E Skåne	2022	Öland	2021	Modern	182.25	0.03199	0.03113	0.03290
P. argus	E Skåne	2022	Småland	2021	Modern	193.41	0.03176	0.03088	0.03269
P. argus	Småland	2021	SW Skåne	2021	Modern	244.67	0.04090	0.03989	0.04193
P. argus	Öland	2021	SW Skåne	2021	Modern	268.99	0.04129	0.04027	0.04236
C. semiargus	E Skåne	1956	W Skåne	1951	Historical	45.61	0.02874	0.02802	0.02948
C. semiargus	E Skåne	1956	Småland	1936	Historical	166.1	0.04307	0.04187	0.04428
C. semiargus	Småland	1936	W Skåne	1951	Historical	193.88	0.05167	0.05040	0.05299
C. semiargus	E Skåne	2021	SE Skåne	2022	Modern	43.61	0.08680	0.08562	0.08800
C. semiargus	E Skåne	2021	W Skåne	2021	Modern	44.34	0.04940	0.04859	0.05022
C. semiargus	SE Skåne	2022	W Skåne	2021	Modern	49.72	0.10919	0.10788	0.11048
C. semiargus	E Skåne	2021	Småland	2021	Modern	191.11	0.06224	0.06124	0.06328
C. semiargus	Småland	2021	W Skåne	2021	Modern	206.5	0.08387	0.08267	0.08506
C. semiargus	Småland	2021	SE Skåne	2022	Modern	234.47	0.12170	0.12022	0.12318

Table S8. Convergence results for admixture analyses. For values of K 1-8, we performed up to 100 replicate admixture analyses per species to assess convergence, performing a minimum of 20 replicates. If the three highest likelihood replicates were within 2 log-likelihood units of each other, the replicates were considered converged and no further replicates were performed. We selected the highest value of K per species that converged as the 'best fit', which are highlighted here in bold, presented with the total number of replicates required to reach convergence, as well as the difference in log-likelihood between the top 3 replicates.

Species	K	Replicates to convergence (max 100)	Δ log-likelihood of top 3 replicates
Polyommatus icarus	**1**	**20**	**0.000**
Polyommatus icarus	2	100	85.270
Polyommatus icarus	3	100	1820.431
Polyommatus icarus	4	100	1820.550
Polyommatus icarus	5	100	4206.780
Polyommatus icarus	6	100	2362.288
Polyommatus icarus	7	100	2536.728
Polyommatus icarus	8	100	3810.643
Plebejus argus	**1**	**20**	**0.000**
Plebejus argus	2	100	88.839
Plebejus argus	3	100	502.764
Plebejus argus	4	100	999.728
Plebejus argus	5	100	646.737
Plebejus argus	6	100	7439.148
Plebejus argus	7	100	2735.900
Plebejus argus	8	100	6233.690
Cyaniris semiargus	1	20	0.000
Cyaniris semiargus	2	20	0.013
Cyaniris semiargus	3	20	0.007
Cyaniris semiargus	4	20	0.004
Cyaniris semiargus	**5**	**23**	**0.139**
Cyaniris semiargus	6	100	1287.189
Cyaniris semiargus	7	100	6647.493
Cyaniris semiargus	8	100	4275.050

Table S9. Statistical testing of differences in inbreeding between modern and historical samples. For each species, we tested for differences in the inbreeding coefficient Froh using a linear mixed model with Froh as a response to the fixed effect of time period and the random effect of sampling region. Outputs from the models are summarized here.

Species	Mean Froh (hist)	Mean Froh (hist, jackkife CI)	Mean Froh (mod)	Mean Froh (mod, jackkife CI)	Difference	Std.Error	t-statistic	95% CI (lower)	95% CI (upper)	p-value
Po. icarus	0.0009	[0.00096, 0.00107]	0.00107	[0.00071, 0.00111]	0.00019	0.0008	0.23486	-0.00143	0.0018	0.81555
Pl. argus	0.00767	[0.00556, 0.00848]	0.00969	[0.00969, 0.00888]	0.00266	0.00353	0.75277	-0.00452	0.00984	0.45677
Cy. semiargus	0.00771	[0.0059, 0.00835]	0.07248	[0.06691, 0.075]	0.06477	0.0205	3.15983	0.02327	0.10626	**0.00309**

Table S10. Predicted retention of heterozygosity, mean modern Froh, and heterozygosity outside of RoH from coalescent simulations. To assess if our empirical estimates of heterozygosity reduction aligned with expected patterns under inferred population declines, we simulated demographic histories for each population based on size changes inferred with GONE. Here, we document the minimum, quantile, and maximum estimates for three parameters from 100 replicate runs of each simulated history: the proportion of heterozygosity retained in the modern compared to the historical population, the mean estimate of Froh in the modern population, and the proportion of heterozygosity retained adjusted for Froh (i.e. heterozygosity / (1-Froh) estimated in each sampled simulated individual). We find that across all simulations, nearly all changes in heterozygosity over time should be reflected with equivalent changes in Froh.

		Proportion heterozygosity maintained					*Mean modern Froh*					*Proportion heterozygosity maintained outside RoH*				
Species	***Region***	***Min***	***25%***	***50%***	***75%***	***Max***	***Min***	***25%***	***50%***	***75%***	***Max***	***Min***	***25%***	***50%***	***75%***	***Max***
Cy. semiargus	ESkane	0.952	0.976	0.985	0.992	1.01	0.00211	0.0103	0.0177	0.0255	0.0518	0.986	0.995	0.998	1	1.02
Cy. semiargus	NSmaland	0.989	0.996	0.999	1	1.01	0	0.00059	0.0013	0.00295	0.0155	0.989	0.998	0.999	1	1.01
Cy. semiargus	WSkane	0.887	0.928	0.944	0.955	0.982	0.023	0.0428	0.0521	0.0702	0.108	0.981	0.992	0.996	0.998	1

Table S11. Statistical testing of differences in deleterious burden between modern and historical samples. We estimated deleterious burden for three categories of variants: conserved sites (those in the top 1% of GERP scores across the genome), high impact variants (e.g. loss of function, signifying strongly deleterious), and moderate impact variants (e.g. missense, signifying weakly deleteriou). We additionally estimated burden at low impact (i.e. genic synonymous) and intergenic variants, both presumed neutral. For each of these categories, we estimated total deleterious burden as the count of derived allelesof the focal category per 1000 called genotypes. We estimated homozygous burden using the count of homozygous derived alleles of the focal category per 1000 called genotypes. We statistically assessed differences in heterozygosity between modern and historical samples for each species using a linear mixed model with burden estimates as a response to the fixed effect of time period and the random effect of sampling region. Outputs from the models are summarized here.

Species	Mean derived count / 1000 GTs (Hist)	Mean derived count / 1000 GTs (Hist, jackkife CI)	Mean derived count / 1000 GTs (Mod)	Mean derived count / 1000 GTs (Mod, jackknife CI)	Mod/Hist	Difference	Std.Error	t-statistic	95% CI (lower)	95% CI (upper)	p-value	Mutation category
Po. icarus	0.09378	[0.09283, 0.09516]	0.09062	[0.09007, 0.09126]	0.96631	-0.00299	0.00306	-0.97664	-0.00918	0.0032	0.33477	Conserved Homozygous
Po. icarus	1.06868	[1.06531, 1.07287]	1.07351	[1.07154, 1.07582]	1.00452	0.00483	0.01097	0.44066	-0.01735	0.02702	0.66189	Conserved Total
Po. icarus	0.04043	[0.03986, 0.04149]	0.04106	[0.04075, 0.04148]	1.01557	0.00063	0.00198	0.31751	-0.00338	0.00464	0.75255	High Total
Po. icarus	0.00572	[0.00545, 0.00605]	0.00564	[0.00545, 0.0058]	0.98539	-8.00E-05	0.00082	-0.10237	-0.00174	0.00157	0.91899	High Homozygous
Po. icarus	27.94189	[27.90848, 27.99487]	27.81699	[27.80242, 27.83497]	0.99553	-0.11309	0.09335	-1.21144	-0.3019	0.07573	0.23302	Intergenic Total
Po. icarus	5.15418	[5.13338, 5.17225]	5.0749	[5.05985, 5.0855]	0.98462	-0.02557	0.04092	-0.62489	-0.10835	0.0572	0.53568	Intergenic Homozygous
Po. icarus	2.31903	[2.30707, 2.33323]	2.36566	[2.36078, 2.36915]	1.02011	0.04663	0.02664	1.75008	-0.00726	0.10052	0.08797	Low Total
Po. icarus	0.52338	[0.5191, 0.52728]	0.53562	[0.53282, 0.53773]	1.02338	0.01297	0.01183	1.09648	-0.01095	0.03689	0.27959	Low Homozygous
Po. icarus	2.55575	[2.54312, 2.57422]	2.50478	[2.49985, 2.5091]	0.98006	-0.05097	0.03091	-1.64873	-0.1135	0.01156	0.10724	Moderate Total
Po. icarus	0.53694	[0.53361, 0.54052]	0.53737	[0.53433, 0.53993]	1.0008	0.0043	0.01264	0.3402	-0.02127	0.02987	0.73553	Moderate Homozygous
Pl. argus	0.20199	[0.19882, 0.2052]	0.23058	[0.22878, 0.2322]	1.14158	0.0286	0.00984	2.90469	0.00859	0.0486	**0.00642**	Conserved Homozygous
Pl. argus	2.1471	[2.13324, 2.15733]	2.12135	[2.11377, 2.12881]	0.98801	-0.0189	0.03979	-0.47497	-0.09977	0.06197	0.63784	Conserved Total
Pl. argus	0.097	[0.09556, 0.09909]	0.09619	[0.09504, 0.0971]	0.99166	-0.00025	0.00581	-0.04346	-0.01205	0.01155	0.96559	High Total
Pl. argus	0.01984	[0.01878, 0.02068]	0.02051	[0.01978, 0.021]	1.03376	0.00072	0.00302	0.23704	-0.00542	0.00685	0.81405	High Homozygous
Pl. argus	39.8359	[39.75759, 39.912]	39.84086	[39.81737, 39.86539]	1.00012	-0.1089	0.13944	-0.781	-0.39227	0.17447	0.44021	Intergenic Total
Pl. argus	9.10907	[9.01262, 9.17277]	9.66486	[9.63512, 9.68801]	1.06101	0.61549	0.16653	3.69599	0.27706	0.95392	**0.00077**	Intergenic Homozygous
Pl. argus	6.62767	[6.58499, 6.66695]	6.6161	[6.60502, 6.63723]	0.99825	0.02418	0.08958	0.26991	-0.15788	0.20624	0.78886	Low Total
Pl. argus	1.93521	[1.91264, 1.95777]	1.97182	[1.96363, 1.98321]	1.01892	0.06686	0.05117	1.30673	-0.03712	0.17085	0.20008	Low Homozygous
Pl. argus	6.99366	[6.97011, 7.01974]	6.73786	[6.72622, 6.74883]	0.96342	-0.2398	0.06228	-3.85045	-0.36637	-0.11324	**5.00E-04**	Moderate Total
Pl. argus	1.81401	[1.78761, 1.82562]	1.83072	[1.821, 1.83804]	1.00922	0.02756	0.04659	0.59166	-0.06711	0.12224	0.55799	Moderate Homozygous
Cy. semiargus	0.20064	[0.19728, 0.2041]	0.26764	[0.26078, 0.27093]	1.33396	0.06612	0.02301	2.87343	0.01954	0.11271	**0.00661**	Conserved Homozygous
Cy. semiargus	2.00528	[1.98799, 2.02101]	2.04377	[2.03818, 2.04721]	1.01919	0.0382	0.02947	1.29633	-0.02146	0.09787	0.20268	Conserved Total
Cy. semiargus	0.08421	[0.0828, 0.08613]	0.08648	[0.08528, 0.08735]	1.02693	0.00196	0.00536	0.36607	-0.00889	0.01281	0.71634	High Total
Cy. semiargus	0.02213	[0.02112, 0.02297]	0.02681	[0.02532, 0.0276]	1.21112	0.00467	0.00536	0.87202	-0.00618	0.01552	0.38867	High Homozygous
Cy. semiargus	52.48658	[52.4083, 52.66116]	52.95155	[52.88969, 52.99469]	1.00886	0.44782	0.29089	1.53945	-0.14107	1.0367	0.13198	Intergenic Total
Cy. semiargus	11.10881	[10.95993, 11.20657]	14.329	[14.05913, 14.4549]	1.28988	3.22019	1.05601	3.04939	1.08241	5.35798	**0.00416**	Intergenic Homozygous
Cy. semiargus	5.14493	[5.07654, 5.18325]	5.1522	[5.1431, 5.16379]	1.00141	0.04006	0.0679	0.59001	-0.0974	0.17753	0.55867	Low Total
Cy. semiargus	1.24445	[1.21954, 1.26001]	1.53844	[1.5094, 1.55292]	1.23624	0.29481	0.09787	3.01218	0.09668	0.49294	**0.0046**	Low Homozygous
Cy. semiargus	5.85531	[5.79237, 5.88862]	5.81692	[5.80796, 5.82559]	0.99344	-0.0236	0.07358	-0.32076	-0.17256	0.12535	0.75015	Moderate Total
Cy. semiargus	1.32953	[1.31242, 1.34297]	1.66162	[1.63191, 1.67645]	1.24978	0.32898	0.10274	3.20196	0.12099	0.53698	**0.00276**	Moderate Homozygous

Table S12. Study species conservation status and reference genome information. For each study species, the conservation status in Sweden is presented based on the most recent assessment, the 2020 Swedish Red List. Sequencing data for each species was mapped to the closest available chromosome level reference genome. For the three focal species, and all but one of the additional species, this was a species specific genome. For *Plebejus argyrognomon* , the closest reference (*Pl. argus*) was used. For inbreeding and GONE analyses, we utilized a recombination rate based off the assumption of 25 centimorgans per chromosome, which were used to calculate the autosomal recombination rates for the reference genome species in Morgans per base pair per generation.

Study Species	Swedish Red List 2020 Status	Reference species	RefSeq Assembly Accession	Assembly Name	Autosomal Chromosomes	Autosomal Length (bp)	Recombination rate (M/bp)
Polyommatus icarus	LC (Livskraftig; Least Concern)	*Polyommatus icarus*	GCA_937595015.1	ilPolIcar1.1	22	472204672	1.16475E-08
Plebejus argus	LC (Livskraftig; Least Concern)	*Plebejus argus*	GCA_905404155.3	ilPleArgu1.3	22	360412310	1.52603E-08
Cyaniris semiargus	LC (Livskraftig; Least Concern)	*Cyaniris semiargus*	GCA_905187585.1	ilCyaSemi1.1	23	416757882	1.3797E-08
Agriades optilete	LC (Livskraftig; Least Concern)	*Agriades optilete*	GCA_964273475.1	ilAgrOpti3.hap1.1	23	607663052	9.46248E-09
Aricia agestis	LC (Livskraftig; Least Concern)	*Aricia agestis*	GCF_905147365.1	ilAriAges1.1	22	394696725	1.39347E-08
Cupido Minimus	NT (Nära hotad; Near Threatened)	*Cupido minimus*	GCA_965195375.1	ilCupMini1.1	23	448207675	1.28289E-08
Plebejus argyrognomon	CR (Akut hotad; Critically endangered)	***Plebejus argus****	GCA_905404155.3	ilPleArgu1.3	22	360412310	1.52603E-08
Scolitantides orion	EN (Starkt hotad; Endangered)	*Scolitantides orion*	GCA_964345685.1	ilScoOrio1.hap1.1	22	468110494	1.17494E-08

Table S13. Species dataset sequence data filtering. For each species dataset, we generated a 'filtered sites' file to limit analyses to. This filtered sites file is the intersection of several independent filters that remove scaffold smaller than 1Mb, non-autosomal contigs, repetitive regions, and regions in the top and bottom 1% of global sequencing depth for all sample, museum sample, and fresh sample groupings. Here, the number of base pairs and the proportion of the genome passing each filter is shown for each of the species datasets, as well as these parameters for the final combined filtered sites file. In some cases, only museum samples were utilized for a species, in which case the depth filters are only performed on one sample grouping, which is the same as the filtering across all samples. The reference genome used for each species is listed at the top of the table, and has a * when it is from a different species to the study species.

	Polyommatus icarus (ilPolIcar1.1)		*Plebejus argus* (ilPleArgu1.3)		*Cyaniris semiargus* (ilCyaSemi1.1)		*Aricia agestis* (ilAriAges2.1)	
**Filter**	**Filtered Length(bp)**	**Filtered percent**	**Filtered Length(bp)**	**Filtered percent**	**Filtered Length(bp)**	**Filtered percent**	**Length(bp)**	**Percent**
Total genome	511758028	100	382108302	100	441519306	100	437383100	100
Scaffolds<1000000bp	511184185	99.8879	379254911	99.2533	441467852	99.9883	437193358	99.9566
Autosomes	472204672	92.2711	363250311	95.0648	416794061	94.4	394871022	90.2804
Repeats	230539290	45.0485	207343925	54.2631	217090499	49.169	219807041	50.255
Depth (all)	428236719	83.6795	328918008	86.0798	388536918	88	335802494	76.7754
Depth (museum)	355099842	69.3882	328310056	85.9207	388068388	87.8939	329841456	75.4125
Depth (fresh)	427116071	83.4606	269759492	70.5977	347302061	78.6607	273127265	62.4458
**Combined**	201529665	39.3799	186409699	48.7845	195370411	44.2496	179516799	41.0434

	Agriades optilete (ilAgrOpti3.hap1.1)		*Cupido minimus* (ilCupMini1.1)		*Plebejus argyrognomon* (ilPleArgu1.3*)		*Scolitantides orion* (ilScoOrio1.hap1.1)	
**Filter**	**Length(bp)**	**Percent**	**Length(bp)**	**Percent**	**Length(bp)**	**Percent**	**Length(bp)**	**Percent**
Total genome	680738285	100	474403353	100	382108302	100	525334176	100
Scaffolds<1000000bp	677998561	99.5975	474293063	99.9768	379254911	99.2533	524519661	99.845
Autosomes	607663052	89.2653	448207675	94.4782	363250311	95.0648	468110494	89.1072
Repeats	231683966	34.0342	211975703	44.6826	207343925	54.2631	210604438	40.0896
Depth (all)	520387915	76.4446	345703778	72.8713	142493774	37.2915	381912722	72.699
Depth (museum)	517527179	76.0244	340141899	71.6989	142493774	37.2915	381912722	72.699
Depth (fresh)	377891541	55.512	279749861	58.9688	NA	NA	NA	NA
**Combined**	182033741	26.7406	174264760	36.7335	124475234	32.5759	176286414	33.557

Table S14. Removal of close-relatives from admixture/PCA. As close relatives can skew the results of principal component and admixture analyses, we removed third-degree and closer relatives from these analyses. We estimated relatedness using the IBSrelate method, identifying first degree (PO/FS; KING 0.25), second-degree (HS; KING 0.125), and third-degree (C1; KING 0.0625) relatives and removing one of each pair from the analyses. The removed individuals are highlight in bold and red text. These individuals were included in all other analyses.

Species	Sample 1	Sample 2	R0	R1	KING	Inferred Relationship
Po. icarus	**PI031**	PI033	0.2633	0.291742	0.0917	C1
Pl. argus	**MZLU107435**	MZLU107438	0.2510	0.2997	0.0979	C1
Cy. semiargus	**MZLU107455**	MZLU107461	0.025537	0.780051	0.291401	FS
Cy. semiargus	MZLU153216	**MZLU153221**	0.073815	0.605471	0.238239	FS
Cy. semiargus	CYSE0078	**CYSE0098**	0.212413	0.323411	0.117873	HS
Cy. semiargus	MZLU107456	**MZLU107501**	0.237071	0.346552	0.113124	HS
Cy. semiargus	CYSE0198	**CYSE0199**	0.271307	0.290711	0.088494	C1

Table S15. Reference genomes utilized for estimating GERP scores. We utilized the method implemented in the GenErode pipeline (Kutschera et al. 2022) to estimate GERP scores and ancestral states across the reference genomes for each species dataset. Below is the list of reference genomes, their accessions, and the reference species. We utilized all genomes from this list when estimating GERP scores and ancestral states along each of the focal species reference genomes, excluding the focal species.

RefSeq Assembly Accession	Assembly Name	Organism_Name	Genome Note Reference
GCA_964273475.1	ilAgrOpti3.hap1.1	Agriades optilete	NA
GCF_905147365.1	ilAriAges1.1	Aricia agestis	NA
GCA_965178315.1	ilCalAvis1.hap1.1	Callophrys avis	NA
GCA_905187575.2	ilCelArgi3.2	Celastrina argiolus	DToL Consortium, Hayward, et al. 2021
GCA_965195375.1	ilCupMini1.1	Cupido minimus	NA
GCA_905187585.1	ilCyaSemi1.1	Cyaniris semiargus	Lohse, Hayward, et al. 2023
GCA_964396525.1	ilAriEume1.hap1.1	Eumedonia eumedon	NA
GCA_905404095.1	ilGlaAlex1.1	Glaucopsyche alexis	DToL Consortium, Hinojosa Galisteo, et al. 2021
GCA_963853865.1	ilHelHell1.1	Helleia helle	NA
GCA_964662265.1	ilQueQuer1.hap2.1	Hypaurotis quercus	NA
GCA_965112825.1	illolDebi1.hap1.1	Iolana debilitata	NA
GCA_964264435.1	ilKreTrap1.hap1.1	Kretania trappi	NA
GCA_964417165.1	ilLaeRobo1.hap1.1	Laeosopis roboris	NA
GCA_964656195.1	ilLamBoet1.hap1.1	Lampides boeticus	NA
GCA_965112865.1	ilLepPiri1.hap1.1	Leptotes pirithous	NA
GCA_905333005.2	ilLycPhla1.2	Lycaena phlaeas	Lohse et al. 2021
GCA_905333045.1	ilLysBell1.1	Lysandra bellargus	DToL Consortium et al. 2022
GCA_963565745.1	ilPheArio1.1	Phengaris arion	NA
GCA_905404155.3	ilPleArgu1.3	Plebejus argus	Hayward et al. 2022
GCA_937595015.1	ilPolIcar1.1	Polyommatus icarus	Lohse , Wellcome Sanger Institute Tree of Life programme, et al. 2023
GCA_963422495.1	ilPolIphe1.1	Polyommatus iphigenia	NA
GCA_965178345.1	ilSatIlic2.hap1.1	Satyrium ilicis	NA
GCA_964345685.1	ilScoOrio1.hap1.1	Scolitantides orion	NA
GCA_965153325.1	ilTomBall1.hap1.1	Tomares ballus	NA

Supplementary References

Andersson A, Karlsson S, Ryman N, Laikre L. 2022. Monitoring genetic diversity with new indicators applied to an alpine freshwater top predator. Molecular Ecology. 31(24):6422–6439. https://doi.org/10.1111/mec.16710

Broad Institute. 2019. Picard toolkit. Broad Institute, GitHub Repository. [published online ahead of print] https://broadinstitute.github.io/picard/

Chen S, Zhou Y, Chen Y, Gu J. 2018. fastp: an ultra-fast all-in-one FASTQ preprocessor. Bioinformatics. 34(17):i884–i890. https://doi.org/10.1093/bioinformatics/bty560

Danecek P et al. 2021. Twelve years of SAMtools and BCFtools. GigaScience. 10(2) https://doi.org/10.1093/gigascience/giab008. https://doi.org/10.1093/gigascience/giab008

Darwin Tree of Life Consortium, Hayward A, et al. 2021. The genome sequence of the holly blue, *Celastrina argiolus* (Linnaeus, 1758) [version 1; peer review: 2 approved]. Wellcome Open Research. 6(340) https://wellcomeopenresearch.org/articles/6-340/v1. https://doi.org/10.12688/wellcomeopenres.17478.1

Darwin Tree of Life Consortium, Hinojosa Galisteo J, et al. 2021. The genome sequence of the green-underside blue, *Glaucopsyche alexis* (Poda, 1761) [version 1; peer review: 2 approved]. Wellcome Open Research. 6(274) https://wellcomeopenresearch.org/articles/6-274/v1. https://doi.org/10.12688/wellcomeopenres.17264.1

Darwin Tree of Life Consortium et al. 2022. The genome sequence of the Adonis blue, *Lysandra bellargus* (Rottemburg, 1775) [version 1; peer review: 2 approved, 2 approved with reservations]. Wellcome Open Research. 7(255) https://wellcomeopenresearch.org/articles/7-255/v1. https://doi.org/10.12688/wellcomeopenres.18330.1

Dolenz S et al. 2024. Unravelling reference bias in ancient DNA datasets. Bioinformatics. 40(7):btae436. https://doi.org/10.1093/bioinformatics/btae436

Flynn JM et al. 2020. RepeatModeler2 for automated genomic discovery of transposable element families. Proceedings of the National Academy of Sciences. 117(17):9451–9457. https://doi.org/10.1073/pnas.1921046117

Garcia-Erill G, Albrechtsen A. 2020. Evaluation of model fit of inferred admixture proportions. Molecular Ecology Resources. 20(4):936–949. https://doi.org/10.1111/1755-0998.13171

Garrison E et al. 2018. Variation graph toolkit improves read mapping by representing genetic variation in the reference. Nat Biotechnol. 36(9):875–879. https://doi.org/10.1038/nbt.4227

Hayward A et al. 2022. The genome sequence of the silver-studded blue, *Plebejus argus* (Linnaeus, 1758) [version 1; peer review: 2 approved]. Wellcome Open Research. 7(315) https://wellcomeopenresearch.org/articles/7-315/v1. https://doi.org/10.12688/wellcomeopenres.18607.1

Jun G, Wing MK, Abecasis GR, Kang HM. 2015. An efficient and scalable analysis framework for variant extraction and refinement from population-scale DNA sequence data. Genome Res. 25(6):918–925. https://doi.org/10.1101/gr.176552.114

Kircher M. 2012. Analysis of High-Throughput Ancient DNA Sequencing Data. In: Shapiro B, Hofreiter M, editors. Ancient DNA: Methods and Protocols. Humana Press; p 197–228 [accessed 2025 May 1]. https://doi.org/10.1007/978-1-61779-516-9_23. https://doi.org/10.1007/978-1-61779-516-9_23

Korneliussen TS, Albrechtsen A, Nielsen R. 2014. ANGSD: Analysis of Next Generation Sequencing Data. BMC Bioinformatics. 15(1):1–13. https://doi.org/10.1186/s12859-014-0356-4

Kutschera VE et al. 2022. GenErode: a bioinformatics pipeline to investigate genome erosion in endangered and extinct species. BMC Bioinformatics. 23(1):1–17. https://doi.org/10.1186/s12859-022-04757-0

Li H. 2013. Aligning sequence reads, clone sequences and assembly contigs with BWA-MEM. [accessed 2023 Apr 2]. http://arxiv.org/abs/1303.3997. https://doi.org/10.48550/arXiv.1303.3997

Li H, Durbin R. 2009. Fast and accurate short read alignment with Burrows–Wheeler transform. Bioinformatics. 25(14):1754–1760. https://doi.org/10.1093/bioinformatics/btp324

Lohse K et al. 2021. The genome sequence of the small copper, *Lycaena phlaeas* (Linnaeus, 1760) [version 1; peer review: 2 approved with reservations]. Wellcome Open Research. 6(294) https://wellcomeopenresearch.org/articles/6-294/v1. https://doi.org/10.12688/wellcomeopenres.17289.1

Lohse K, Wellcome Sanger Institute Tree of Life programme, et al. 2023. The genome sequence of the Common Blue, *Polyommatus icarus* (Rottemburg, 1775) [version 1; peer review: 1 approved]. Wellcome Open Research. 8(72). https://doi.org/10.12688/wellcomeopenres.18772.1

Lohse K, Hayward A, et al. 2023. The genome sequence of the Mazarine Blue, *Cyaniris semiargus* (Rottemburg, 1775) [version 1; peer review: 2 approved]. Wellcome Open Research. 8(181). https://doi.org/10.12688/wellcomeopenres.19362.1

Lou RN, Jacobs A, Wilder AP, Therkildsen NO. 2021. A beginner's guide to low-coverage whole genome sequencing for population genomics. Molecular Ecology. 30(23):5966–5993. https://doi.org/10.1111/mec.16077

Martiniano R et al. 2020. Removing reference bias and improving indel calling in ancient DNA data analysis by mapping to a sequence variation graph. Genome Biol. 21:250. https://doi.org/10.1186/s13059-020-02160-7

Neukamm J, Peltzer A, Nieselt K. 2021. DamageProfiler: fast damage pattern calculation for ancient DNA. Bioinformatics. 37(20):3652–3653. https://doi.org/10.1093/bioinformatics/btab190

Nolen ZJ, Rundlöf M, Runemark A. 2024. Species-specific erosion of genetic diversity in grassland butterflies depends on landscape land cover. Biological Conservation. 296:110694. https://doi.org/10.1016/j.biocon.2024.110694

Pečnerová P et al. 2021. High genetic diversity and low differentiation reflect the ecological versatility of the African leopard. Current Biology. 31(9):1862-1871.e5. https://doi.org/10.1016/j.cub.2021.01.064

Peltzer A et al. 2016. EAGER: efficient ancient genome reconstruction. Genome Biol. 17(1):1–14. https://doi.org/10.1186/s13059-016-0918-z

Santiago E et al. 2020. Recent Demographic History Inferred by High-Resolution Analysis of Linkage Disequilibrium. Molecular Biology and Evolution. 37(12):3642–3653. https://doi.org/10.1093/molbev/msaa169

Schubert M et al. 2012. Improving ancient DNA read mapping against modern reference genomes. BMC Genomics. 13(1):178. https://doi.org/10.1186/1471-2164-13-178

Smit A, Hubley R, Green P. 2013. RepeatMasker Open-4.0. http://www.repeatmasker.org

The Darwin Tree of Life Project Consortium. 2022. Sequence locally, think globally: The Darwin Tree of Life Project. Proceedings of the National Academy of Sciences. 119(4):e2115642118. https://doi.org/10.1073/pnas.2115642118

Van der Auwera GA, O'Connor BD. 2020. Genomics in the Cloud: Using Docker, GATK, and WDL in Terra. O'Reilly Media, Inc.

Webster MT, Beaurepaire A, Neumann P, Stolle E. 2023. Population Genomics for Insect Conservation. Annual Review of Animal Biosciences. 11(1):115–140. https://doi.org/10.1146/annurev-animal-122221-075025
